# Documenting Research with Transgender, Nonbinary, and Other Gender Diverse (Trans) Individuals and Communities: Introducing the Global Trans Research Evidence Map

**DOI:** 10.1089/trgh.2018.0020

**Published:** 2019-03-01

**Authors:** Zack Marshall, Vivian Welch, Alexa Minichiello, Michelle Swab, Fern Brunger, Chris Kaposy

**Affiliations:** ^1^ School of Social Work, Faculty of Arts, McGill University, Montreal, Canada.; ^2^ Division of Community Health and Humanities, Faculty of Medicine, Memorial University, St. John's, Canada.; ^3^ Bruyère Research Institute, Ottawa, Canada.; ^4^ School of Epidemiology, Public Health and Preventive Medicine, Faculty of Medicine, University of Ottawa, Ottawa, Canada.; ^5^ Health Sciences Library, Faculty of Medicine, Memorial University, St. John's, Canada.

**Keywords:** evidence map, gender diverse, knowledge synthesis, research ethics, social determinants of health, transgender

## Abstract

There is limited information about how transgender, nonbinary, and other gender diverse (trans) people have been studied and represented by researchers. The objectives of this study were to: (1) increase access to trans research; (2) map and describe trans research across subject fields; and (3) identify evidence gaps and opportunities for more responsible research. Eligibility criteria were established to include empirical research of any design, which included trans participants or their personal information and that was published in English in peer-reviewed journals. A search of 15 academic databases resulted in 25,230 references; data presented include 690 trans-focused articles that met the screening criteria and were published between 2010 and 2014. The 10 topics studied most frequently were: (1) therapeutics and surgeries; (2) gender identity and expression; (3) mental health; (4) biology and physiology; (5) discrimination and marginalization; (6) physical health; (7) sexual health, HIV, and sexually transmitted infections; (8) health and mental health services; (9) social support, relationships, and families; and (10) resilience, well-being, and quality of life. This map also highlights the relatively minor attention that has been paid to a number of study topics, including ethnicity, culture, race, and racialization; housing; income; employment; and space and place. Results of this review have the potential to increase awareness of existing trans research, to characterize evidence gaps, and to inform strategic research prioritization. With this information, it is more likely that trans communities and allies will be in a position to benefit from existing research and to hold researchers accountable.

## Introduction

Systematic review methodologies, including scoping reviews and evidence maps, provide an opportunity to study detailed aspects of knowledge production, including what topics are researched, who tends to be studied, what types of methods are used, and how people interact with the products of research. In this way, reviews turn the focus of attention toward the research process and researchers themselves, uncovering new information and increasing the visibility of diverse fields of study.

The aim of this review is to map and describe how transgender, nonbinary, and other gender diverse (trans) people have been studied and represented within and across research in the fields of social sciences, humanities, health, sciences, business, and education. The term “trans” refers to people who “do not conform to prevailing expectations about gender” (Terminology section, para. 1)^1^ and includes transgender, transsexual, and other gender diverse people of all ages. In contrast, the term cisgender refers to people who identify with the gender they were labeled at birth.^2^ While trans is a self-identification, it also relates to a psychiatric diagnosis.^3^ Transsexual and transgender people diagnosed with gender identity disorder or gender dysphoria have been the subjects of medical and psychiatric research and are described in clinical and social science literature. In this review of trans research, we have opted for a broad trans conceptualization^4^ that incorporates diverse gender identities and expressions across global contexts. This includes transgender and transsexual people as well as drag queens, butch femmes, Two-Spirit people, hijra, travesti, cross-dressers, and additional nonbinary and gender diverse identities and expressions.

Knowledge about the scope of research focusing on trans individuals and communities is incomplete. Because many people are unaware of the extent of research that has been carried out, this leads to miscommunication and misinterpretations. Such misunderstandings may be particularly troublesome if trans community members are not aware of existing research evidence related to the questions they have about their lives. Systematic research detailing the nature of studies that have been conducted provides new insights into the evidence that does exist and can aid in identifying opportunities for more responsible research^5^ with trans individuals and communities.

Multiple challenges constrain our ability to conduct reviews in the field of trans research. The first relates to the language used to describe transgender and nonbinary people and the ways this impacts search strategies. Terminology to describe gender diverse people differs across stakeholder communities, including language used within communities, medical diagnoses, and phrases distinct to linguistic or cross-cultural groups. As this language develops over time,^6^ it adds to the diversity of terms that should be incorporated into effective search strategies. A second barrier relates to indexed subject headings, both in terms of their inability to remain up to date, and the ways these headings reflect the spectrum of trans experience.^7^ These complications require searches that go beyond subject headings, a process that is made more convoluted because it is difficult to search terms such as “trans” or “gender identity” by themselves due to the lack of specificity of these terms and the consequent number of extraneous results this produces. Search strategies also need to include database-specific headings and independent search terms such as vaginoplasty or mastectomy that may be germane to both cisgender and transgender experiences. Once searches are complete, screening is impacted by problems identifying whether the study includes any trans participants, or whether the research is trans focused, due to incomplete and/or unclear information in the title and abstract. For example, these difficulties arise when reviewing references that include trans participants within larger studies with lesbian, gay, bisexual, trans, Two-Spirit, and queer (LGBT2Q) communities, and surgery-related case reports. In their recent systematic review of gaps and opportunities in primary care preventative health services for trans people, Edmiston et al.^8^ reported similar challenges.

Despite these circumstances, some researchers have attempted to increase awareness of the types of trans research available. One of the earliest examples was published by Denny as an annotated bibliography in 1994,^9^ including a classification of books, articles, and community reports. Since then, the number of systematic reviews has slowly increased. Primarily health focused,^10^ researchers have conducted trans-focused reviews related to mental health,^11^ gender dysphoria,^12^ learning disabilities,^13^ aging,^14^ cancer care,^15^ and HIV.^16^ More commonly, trans research is included as part of larger reviews centering men who have sex with men (MSM), LGBT2Q communities, or other marginalized populations.^17,18^

Combining a comprehensive search strategy, text mining, and evidence map, this investigation has the potential to enhance knowledge in several fields. There are currently no evidence maps of trans research. By documenting this broad field of study, this review will enhance awareness of existing trans research, highlight evidence gaps, and inform strategic research prioritization.^19^ Publishing the map online will also expand access to research for key stakeholders, including community members, policymakers, and health care providers.

## Materials and Methods

Evidence maps are an emerging research method^20^ to “collate, describe, and catalog” knowledge across a broad field of study.^21^ This information can then be leveraged by stakeholders to inform policy and clinical decision-making.^21^ This evidence map was developed using the four-step framework introduced by Hetrick et al.^22^: identify objectives, describe characteristics to be mapped and eligibility criteria, screen the literature, and chart the study within the map. The protocol for this evidence map was previously published^23^ in agreement with the Preferred Reporting Items for Systematic Review and Meta-Analysis Protocols (PRISMA-P).^24^

### Aim and objectives

The aim of this study was to map and describe how trans people have been studied and represented within and across multiple fields of research. The objectives were to

(1) increase access to research that includes trans people for community members, health care providers, and policymakers by establishing an online evidence map, including a searchable reference database;
(2) document trans research in the fields of social sciences, health, sciences, education, humanities, and business, including information about sample demographics, study topic, and study design; and
(3) characterize evidence gaps and opportunities for more responsible research with trans individuals and communities.

### Eligibility criteria

It is suggested that researchers clarify concepts and engage key stakeholders as part of the process of developing evidence maps.^25^ Accordingly, one-on-one consultations were held with members of trans and cisgender communities to discuss search scope, terminology, and possible uses of an evidence map. Based on the results of pilot searches and consultations, the eligibility criteria were established to include empirical research studies of any design with human participants, which identifiably included trans people or their personal information, and were reported in English in peer-reviewed journals.

### Information sources

The identification of academic databases was informed by the larger goal of locating trans research from multiple fields. A secondary emphasis was to gather research on a global scale. For example, to appropriately identify research related to gender diverse Indigenous people, three databases focused on Indigenous and First Nations research were included.

Fifteen databases were selected to ensure diverse study design identification,^26^ including Academic Search Premier, Anthropology Plus, Bibliography of Native North Americans, CINAHL, First Nations Periodical Index, Indigenous Studies Portal, LILACS, ProQuest Social Sciences Premium (contains ERIC, Social Services Abstracts & Applied Social Sciences Index and Abstracts, and Sociological Abstracts,), PsycINFO, PubMed, SciELO, Scopus, Social Work Abstracts, Web of Science, and Women's Studies International.

### Search strategy

Search terms concentrated on transgender, non-binary, and other gender diverse experiences and identities. Because there are multiple terms used for (and/or by) trans people, and this language continues to evolve over time,^6^ the full list of search terms was wide-ranging and comprised terms linked to gender identity (e.g., “trans woman”), diagnoses (e.g., “gender dysphoria” and “gender identity disorder”), therapeutics and surgical procedures (e.g., facial feminization), language that was used historically (e.g., transvestite), and words used in a range of cultures and countries (e.g., waria, travesti, Two-Spirit, and hijra). A sample search strategy for one academic database is provided in Supplementary Data S1.

### Data management

**FIG. 1.**
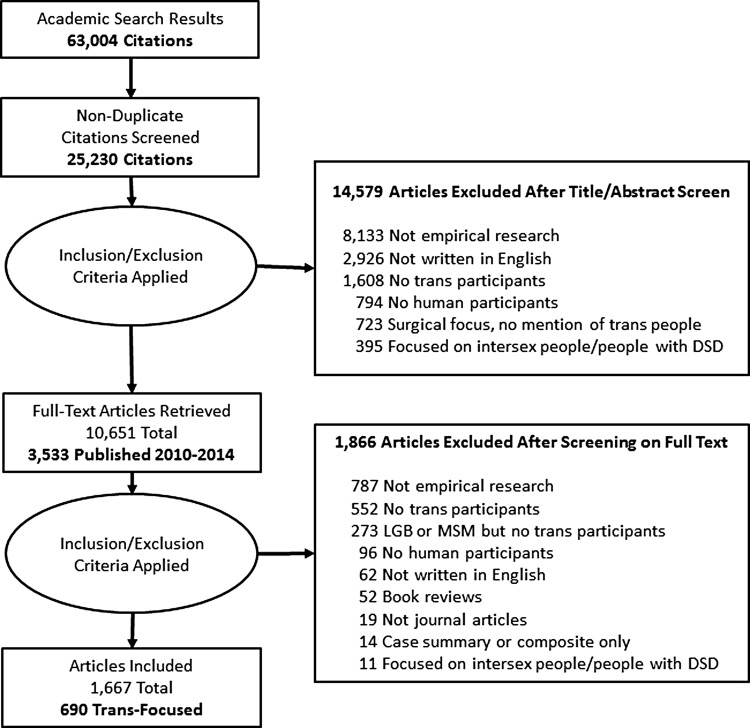
PRISMA flow diagram. PRISMA, Preferred Reporting Items for Systematic Review and Meta-Analysis.

A health sciences librarian reviewed the draft search. Pilot searches were conducted in January 2015 in all 15 databases for each search string to ensure that the search was specific, but not overly sensitive. Full searches were then carried out between January 25 and February 22, 2015. Searches resulted in a total of 63,004 references (Table 1). After eliminating duplicates, the total number of references included in the review was 25,230 (Fig. 1).

**Table 1.** Search Results

Database	*N* records
Academic Search Premier	9,477
Anthropology Plus	339
Bibliography of Native North Americans	75
CINAHL	2386
First Nations Periodical Index	41
Indigenous Studies Portal	84
LILACS	738
ProQuest Social Sciences Premium	10,212
ProQuest Subject Terms	2,718
PsycINFO	6,223
PubMed	7,464
SciELO	482
Scopus	11,640
Social Work Abstracts	144
Web of Science	7,641
Women's Studies International	3,320
Total No. references retrieved	63,004
Duplicates removed	37,758
Empty records deleted	16
Total No. of references	25,230

### Selection process

#### Screening on title and abstract

The first author developed the initial approach to screening and 2 reviewers conducted a pilot review of a random sample of 100 references. This was followed by a follow-up review of a random sample of ∼10% of the dataset (2,393 references). Differences were reconciled through discussion and clarification, leading to a refinement of the eligibility criteria. After this, references were randomly allocated into groups of 1,000. Two reviewers screened the first two groups, reconciling differences through dialogue and discussion.

Reference screening was conducted based on the content of the title and abstract (level 1). Studies were excluded if they were not written in English, if they were not empirical research, if they did not include humans, or if they included only cisgender heterosexual people or people diagnosed with disorders of sex development (DSD; sometimes referred to as intersex people). If a reference was not excluded at level 1, the article was uploaded so that the full text could be reviewed (level 2). Any reference with no abstract was automatically screened on full text.

Whether there were any trans participants included in studies was often not clear from the abstracts. For example, in research with a diversity of LGBT2Q participants, authors might have presented the total number of participants in the abstract, or the study may have included trans participants, but they were not necessarily mentioned at this level. Early in the screening process, it became clear that many disagreements between reviewers were linked to a lack of specificity about sample characteristics in the abstract. As a result, it was necessary to automatically include references with LGBT2Q or MSM samples for screening on full text. In addition, due to the connections between HIV, sex work, and trans populations, all references that mentioned sex workers or people living with HIV as participants were automatically screened on full text. Lack of specificity was also a challenge with some clinical case studies. As a result, any study that mentioned trans-specific surgeries or therapeutics was automatically included. Finally, due to diversity within the general population, any study with a sample size over 1,000 was included. The rationale for this was to verify in the full-text article whether surveys included demographic questions inclusive of trans identities, and whether any trans participant had self-identified.

#### Screening on full text

For full-text screening, two team members reviewed each reference, and any difference was reconciled through discussion. The goal of level 2 screening was to identify original research that included trans participants or their personal information. In addition, at this level, we identified three different types of studies: trans focused, LGBT2Q/MSM, and mixed. Trans-focused studies included those with only trans participants as well as those with a cisgender control group. LGBT2Q/MSM studies were studies that included trans people as part of larger studies with sexual and gender diverse participants. Mixed studies were those with both cisgender and trans participants. In addition, studies with photographs were also flagged at this level of screening. The purpose of identifying this information at level 2 was to support data extraction. The evidence map presented in this study included only trans-focused studies.

#### Data items for mapping

Data extraction focused on developing an evidence map that emphasized the distribution and extent^27^ of trans research studies. The following information was collected for mapping: year of publication, study topic, study design, trans sample demographics, data sources, geographic location of data collection, and open access availability. This article focuses on data related to study topic and study design.

### Data analysis

#### Study topics

To develop a list of study topics for the map, the team started with the social determinants of health^28^ and frameworks that incorporate both structural health perspectives and individual health behaviors. Models by Ansari et al.^29^ and Brennan Ramirez et al.^30^ inspired early conceptualizations of topic areas. After piloting, additional subjects were added to the map that helped to expand the coding framework beyond a health focus. New topics that were added included the following: arts and creativity; sex work; resilience, well-being and quality of life; and resistance and activism.

In the first phase of data extraction, one reviewer went through each reference to identify key study topics. In coding for study topic, we focused on the stated purpose as identified by the study author(s). While there was no set limit to the number of study topics that could be selected, we aimed for a range of two to four study topics per reference. In the next phase, a second team member reviewed groups of references by study topic. For example, all references within the study topic of aging or physical health were verified for consistency and topic cohesion. In this phase, some of the more traditional social determinant topics were also renamed to better communicate the subject matter included in that category. For example, natural built environments were reconceptualized as space and place.

In this phase, the second reviewer also conducted word searches within the set of included studies to verify that no relevant references had been excluded. For example, in searching for articles about aging, the dataset was searched for any reference that included relevant search terms such as “age,” “aging,” “elder,” “senior,” and “old” in the title and/or abstract. This not only produced larger sets of references for checking but also helped to ensure that studies relevant to each topic were captured within the map.

#### Study design

Because this review included a broad range of quantitative, qualitative, and clinical study types, it was not possible to use an existing evidence-based categorization scheme. As a result, two of the coauthors developed a coding framework, including the following options: (1) systematic review of randomized controlled trials; (2) randomized controlled trial; (3) nonrandomized controlled trial; (4) case–control study; (5) cohort study; (6) systematic review of descriptive or qualitative studies; (7) cross-sectional study; (8) qualitative study with interviews or focus groups; (9) ethnography or phenomenological qualitative study; (10) historical research; (11) case report, case study, or case series; (12) autoethnography; (13) basic science; and (14) community-based research or other forms of participatory research.

Clear definitions of each study design were identified using the following sources: systematic reviews,^31^ case–control, cohort, and cross-sectional studies,^32–34^ case studies,^35^ and case reports and case series.^36^ To be categorized as a systematic review, studies needed to include a clear search strategy or method to identify studies, and to explicitly state their methods of study selection. Because there are limited systematic reviews in the field of trans studies and this evidence map aimed for broad inclusion, we did not require the third criteria from the PRISMA-P definition of a systematic review (explicitly described methods of synthesis)^31^ in order for studies to be included.

One reviewer extracted information about study design and data collection methods from all trans-focused studies. A second reviewer verified the first 10% of the data extraction. After clarifying any difference in coding, additional questions about how to code particular studies were discussed with a third member of the study team. Based on this information reviewer, two checked the references within each study design, grouping for accuracy and consistency.

## Results

### Screening on title and abstract

A total of 25,230 references were screened based on title and abstract content (level 1). Around 14,579 references were excluded for the following reasons: 8,133 based on study design, 2,926 were not in English, 1,608 because they gave no indication that trans people had been participants, 794 did not include human participants (i.e., they were based on animal models or relied on documents for analysis), 723 were articles about surgery that did not suggest trans participation, and 395 focused on intersex or DSD experience. A total of 6,915 references met the inclusion criteria based on title and abstract, and an additional 3,736 were included based on no abstract being available (Fig. 1).

### Screening on full text

A total of 10,651 references were eligible for screening on full text. Due to resource constraints, the decision was made to focus the first version of the evidence map on the most recent 5-year period. As a result, 3,533 references published between 2010 and 2014 were screened on full text.

A total of 1,667 articles met the inclusion criteria. Six hundred ninety articles were trans focused, 462 included LGBT2Q and/or MSM participants, and 515 included mixed samples. A total of 1,866 studies were excluded based on the following criteria: not empirical research (787 references); no trans participants (552 references); LGB or MSM, but explicitly no trans participants (273 references); no human participants (96 references); not written in English (62 references); book reviews (52 references); not journal articles (19 references); case summary or composite only (14 references); or focused on intersex participants or people diagnosed with DSD (11 references).

The 690 trans-focused articles form the basis of the remaining data analysis for this article (see Supplementary Data S2 for a full list of the trans-focused references). Data on study topics and study design are the focus of the next section, and are summarized in Supplementary Data S3. Combining data about topic and study design provides additional insights into how researchers have chosen to explore trans research topics, including information about areas of overemphasis and underemphasis, topics that could benefit from knowledge synthesis, and areas that need further attention.

### Study topics

**FIG. 2.**
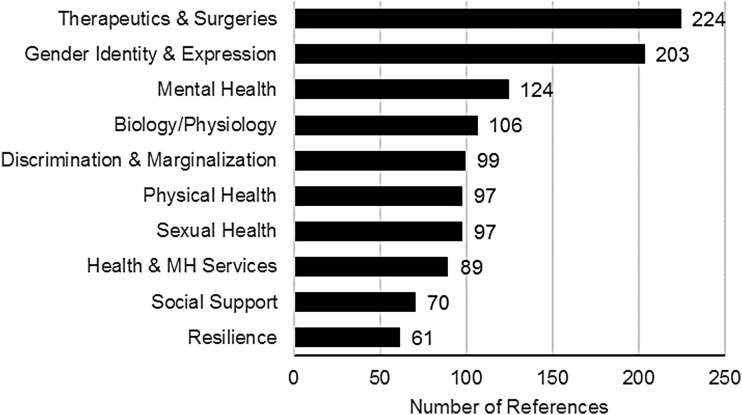
Top 10 study topics.

The map included a total of 37 study topics (Table 2). The top 10 study topics were as follows: (1) therapeutics and surgeries; (2) gender identity and expression; (3) mental health; (4) biology and physiology; (5) discrimination and marginalization; (6) physical health; (7) sexual health, HIV, and sexually transmitted infections (STIs); (8) health and mental health services; (9) social support, relationships, and families; and, (10) resilience, well-being, and quality of life (Fig. 2).

**Table 2.** Summary Table of Study Topics and Frequencies

Study topic	No. of references
Age and aging	12
Arts and creativity	17
Biology and physiology	106
Disability	12
Discrimination and marginalization	99
Early life experiences	40
Education	24
Employment	20
Ethics	9
Ethnicity, culture, race, and racialization	48
Gender identity and expression	203
Health and mental health services	89
Historical perspectives	11
Housing	4
Income	6
Indigeneity	3
Intersectionalities	29
Law and criminalization	24
Mental health	124
Migrant and refugee experiences	2
Other	22
Parenting, reproduction, and assisted reproduction	15
Physical health	97
Religion and spirituality	11
Research methods	24
Resilience/well-being/quality of life	61
Resistance and politicization	34
Sex work	17
Sexual health, HIV, and STIs	97
Sexuality	52
Social support, relationships, and families	70
Space and place	29
Sports and physical activity	5
Substance use (alcohol and drug use)	16
Therapeutic process	34
Therapeutics and surgeries	224
Violence and trauma	47

STIs, sexually transmitted infections.

#### Therapeutics and surgeries

The number one topic area was therapeutics and surgeries, with 224 references. This study topic included gender-affirming processes and procedures such as cross-gender hormone treatment, feminizing or masculinizing procedures such as facial feminization surgery, silicone injection, or electrolysis/laser hair removal, and studies focusing on gender-affirming surgeries such as orchiectomy and vaginoplasty, chest reconstruction, hysterectomy, and phalloplasty. Also included in this category were studies that detailed surgical procedures and outcomes, research and case reports describing side effects of therapeutics or surgeries, and studies exploring levels of satisfaction with gender-affirming medical therapeutics or procedures.

#### Gender identity and expression

Gender identity and expression was the second most common study topic with 203 references. While it was not surprising that a trans research evidence map would include a large number of studies focused on gender identity, efforts were made to clearly distinguish this topic area so that it did not include all studies in the review. Areas of focus included the following: the experience of gender identity, including trans gender identity development; nonbinary and other gender diverse identities; gender dysphoria; gender identity disclosure; medical and social transition; and gender identity assessment and diagnosis.

#### Mental health

Mental health was the third most common study topic with 124 references. This included diagnoses and/or experiences of depression, anxiety, suicide, and other co-occurring mental health diagnoses. This category also included studies documenting the interaction between discrimination, structural oppression, and mental health, and the medicalization and pathologization of gender identity.

#### Biology and physiology

Including 106 studies, the category of biology and physiology includes research at the cellular level, neurological research, bone density studies, and genetic and chromosomal research. In some cases, these studies explored the impacts of medical transition on the physical body. In others, researchers were attempting to identify the etiology of trans gender identity through twin studies, handedness, and measures of cortical thickness.

#### Discrimination and marginalization

There were a total of 99 articles on the topic of discrimination and marginalization. This included studies about different aspects of discrimination such as harassment, bullying, microaggressions, cisgenderism, transphobia, and other forms of oppression. In addition, this topic included research on the topic of social exclusion, stigma, and marginalization. This topic was distinct from violence and trauma, a subject area that included 47 studies. Verbal abuse, physical abuse, and any other form of violence or trauma were included in the latter category.

#### Physical health

The area of physical health had 97 studies, including research related to diabetes, cancer, eating disorders, granulomas, meningiomas, and cardiovascular disease. Some studies explored the link between trans-related therapeutics and longer term health, where others documented complications as a result of surgeries or other medical procedures. Physical health as a study topic was distinct from side effects and impacts of therapeutics and surgeries, and there was little overlap between these two areas of the map. Short-term impacts or complications from surgeries such as chest reconstruction or vaginoplasty were coded within the area of therapeutics and surgeries, whereas longer term health impacts that needed their own intervention were classified under the area of physical health.

#### Sexual health, HIV, and STIs

The category of sexual health, HIV, and STIs included 97 studies about sexual behaviors, and HIV and other STIs. The HIV and STI literature included articles linked to testing, treatment and treatment adherence, transmission, and co-infection, as well as literature that connected HIV and STIs to broader syndemic factors. Sexual health literature included studies about sexual behaviors, communication and negotiation of safer sex behavior, and research related to sexual risk factors. Sexual health was differentiated from the study topic of sexuality, which included 52 references and referred more specifically to sexual attraction and sexual identity.

#### Health and mental health services

Health and mental health services was a relatively large area of the map, including 89 references. These studies investigated barriers and access to health and/or mental health services, experiences with mental health services, discrimination in health care, patient satisfaction, studies of interactions between patients and providers from the trans person's perspective, waitlists, cost-effectiveness, and models of care. This research also explored the impact of barriers to health services on health and mental health.

#### Social support, relationships, and families

Social support, relationships, and families included 70 references. This element of the map included references related to social support and communities, relationships with friends and family, as well as romantic and/or sexual relationships. Social support has been measured and investigated as a factor in relation to health incorporating mental health, physical health, and sexual health. In addition, there were a number of articles related to family support, including family responses to trans children, siblings, or parents.

#### Resilience, well-being, and quality of life

The review included 61 articles on the topic of resilience, well-being, and quality of life. In these strength-based articles, researchers often explored alternate, nonpathologizing conceptualizations of trans lives, including experiences of hope, resilience, and community support.

**FIG. 3.**
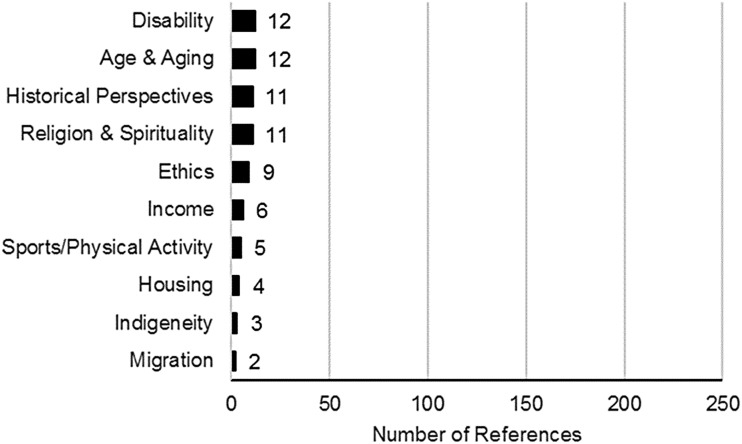
Bottom 10 study topics.

Of the 37 study topics that we categorized, the top 10 most common (listed above), each included at least 50 references. In the mid-range (i.e., between the top 10 and the bottom 10), categories included the following: sexuality; ethnicity, culture, race, and racialization; violence and trauma; early life experiences; resistance and politicization; therapeutic process; intersectionalities^*^; space and place; education; law and criminalization (crime, prisons, incarceration, and policing); research methods; employment; arts and creativity; sex work; substance use; and parenting, reproduction, and assisted reproduction. The bottom 10 topics in the map all included less than 15 references. These were as follows: disability; age and aging; historical perspectives; religion and spirituality; ethics; income; sports and physical activity; housing; Indigeneity; and migration and refugee experiences (Fig. 3).

### Exploring the intersections between study topic and study design

**FIG. 4.**
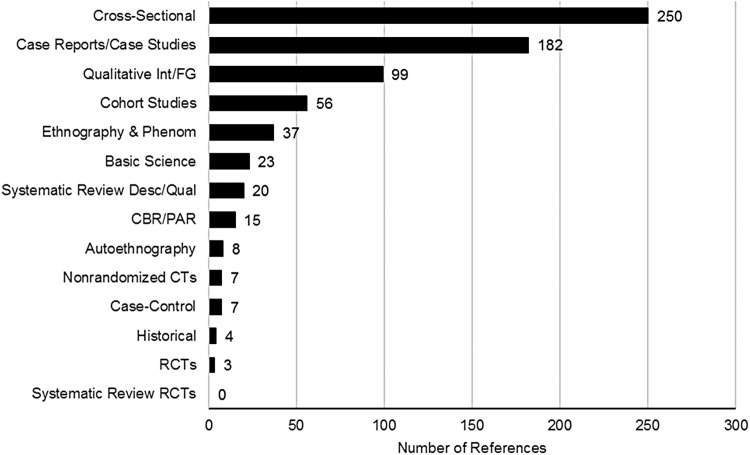
Frequency of study designs.

Of the 690 studies in the review, the emphasis was on observational research. Less than 2% were experimental. The frequency of study design across the trans-focused dataset was: (1) cross-sectional studies (250 references); (2) case reports, case studies, and case series (182 references); (3) qualitative study with interviews or focus groups (99 references); (4) cohort studies (56 references); (5) ethnographies or phenomenological studies (37 references); (6) basic science (23 references); (7) systematic reviews of descriptive or qualitative studies (20 references); (8) community-based research or other participatory research (15 references), (9) autoethnographies (8 references); (10) case–control studies (7 references); (11) nonrandomized controlled trials (7 references); (12) historical research (4 studies); and (13) randomized controlled trials (3 references) (Fig. 4).

The most common research was cross-sectional, emphasizing information gathered at one point in time. Within the top 10 study topics, cross-sectional research was most common in the areas of (1) mental health; (2) gender identity and expression; (3) sexual health, HIV, and STIs; (4) biology and physiology; and (5) therapeutics and surgeries. Cross-sectional research most often involved survey research and clinical measures.

Case reports, case studies, and case series were also very common within the dataset, specifically within the areas of therapeutics and surgeries, and physical health. In these situations, case reports were often used to document novel procedures, surgical complications, or physical side effects related to therapeutics. We also saw the use of case reports and case studies in relation to mental health; gender identity and expression; therapeutic processes; health and mental health services; and sexual health, HIV, and STIs. In the case of health and mental health services, and therapeutic processes, some clinicians reported on client demographics within their clinic, or on the process with specific patients.

Ninety-nine studies included qualitative interviews or focus groups. These methods were particularly relevant when exploring gender identity and expression; discrimination and social exclusion; and social support, relationships, and families. While cross-sectional studies were more frequently used in each of these areas, qualitative interviews or focus groups were the second most common study design for all of these study topics.

## Conclusions

### Topics that received the most attention

Study topics that received the most attention from researchers were as follows: (1) therapeutics and surgeries; (2) gender identity and expression; (3) mental health; (4) biology and physiology; (5) discrimination and marginalization; (6) physical health; (7) sexual health, HIV, and STIs; (8) health and mental health services; (9) social support, relationships, and families; and (10) resilience, well-being, and quality of life. Comparing these results to Reisner et al.'s^10^ review of health-related outcome categories, there were similarities and differences. For example, both reviews share an emphasis on the following topics: (1) mental health; (2) sexual and reproductive health; (3) stigma and discrimination; and (4) general health. In contrast, two topics that were highlighted in Reisner et al.'s^10^ review—substance use, and violence and victimization—did not include a large enough number of studies to be included in the top 10 topics of the evidence map. Some of these differences were linked to Reisner et al.'s^10^ emphasis on quantitative health research. Having a broader subject and methodological focus in this study meant that it was possible to incorporate greater diversity into the evidence map, including research related to therapeutics and surgeries, health and mental health services, social support, and resilience.

### Topics that received the least attention

Topics that have received the least attention include several factors linked to the social determinants of health such as ethnicity and culture, housing, income, employment, and space and place. This review highlights the relatively minor attention invested to date in these study topics and underscores the need to assess whether additional research focused in these areas would be beneficial. For example, given the challenges many trans people face in obtaining employment, research centering on poverty and employment in trans communities, including barriers and facilitators to employment, may be called for. These studies could provide insight into these topics beyond their consideration as risk factors in relation to health and/or mental health.

### Areas that have been systematically reviewed and opportunities for knowledge synthesis

Examining the overlap between the study topics that have received the most attention and existing systematic reviews, there was some positive overlap. For example, gender identity and expression is one of the most researched subject areas and is the topic of five systematic reviews. Similarly, mental health received good attention from researchers and was the focus of five systematic reviews. Sexual health, HIV, and STIs has been the subject of three reviews.

As discussed, therapeutics and surgeries was the most commonly investigated study topic. On the one hand, the ability to conduct reviews in this area was complicated by study designs that tended to emphasize case reports. That said, researchers have taken several approaches to synthesizing knowledge in this area, including case series and analysis of outcomes linked to specific therapeutic interventions or surgeries (e.g., long-term impact of cross-gender hormone treatment, or complications from silicone injection). In addition, although they were not included in this study because they did not meet the criteria for systematic reviews, some authors who are also surgeons review their experiences with surgical procedures, including outcomes and advances in technique.

While 20 systematic reviews of descriptive and qualitative studies have been conducted, there are opportunities for additional knowledge synthesis related to the following: specific aspects of gender identity and expression such as disclosure, or social or medical transition; discrimination and marginalization; physical health; health and mental health services; social support, relationships, and families; and resilience, well-being, and quality of life. Other topics in the map that received less attention (although they each included at least 15 studies) were as follows: sexuality; ethnicity, culture, race, and racialization; violence and trauma; early life experiences; resistance and politicization; education; law and criminalization; employment; arts and creativity; and sex work. These are all relevant and important topics for future systematic or scoping reviews.

### Limitations

The primary limitations of this study relate to resources and technology. Time and financial resources necessitated limiting the map to studies published between 2010 and 2014. To complete the full map, it will be necessary to screen an additional 7,118 references on full text, and references that meet the inclusion criteria will need further data extraction. In addition, to update the map to 2017 would require the searches to be updated and these references would then need to be screened on title and abstract, and where relevant on full text.

Resource constraints have also limited the type of research included in the evidence map. This project is focused on documenting research with trans people from the perspective of human subjects research ethics. As a result, all studies in the map include at least one trans participant. One drawback is that this also means studies about trans topics that do not include trans people are not currently a part of the evidence map. For example, a study to evaluate the knowledge and awareness of health care providers in relation to trans health would not be included, unless it explicitly also included one or more trans participants. While these types of studies form part of the larger field of trans research, this work is not visible in this dataset.

Similarly, the evidence map contains empirical research published in English in peer-reviewed journals. In stating this, it is also important to acknowledge that it does not include solely theoretical, conceptual, or historical work, unless that work is based on original or secondary data analysis with trans participants. There are also no community research reports (sometimes referred to as “gray” literature) or book chapters. In focusing on one aspect of research with trans participants, our intent was not to contribute to making this other work less visible or to imply that it does not constitute an important aspect of the broader field of trans studies.

That the map is already out of date before being published points to the critical need for different ways of working. In time, promising new developments in text mining, automation, and semiautomation will allow us to complete large, living reviews and share this information with key stakeholders in a more timely manner.

### Hesitations: the implications of mapping

There is great potential for this evidence map and the accompanying database to be useful to community members, researchers, clinicians, and policymakers. There are also limitations to how useful it can be to community members if information is not presented in an accessible manner. In addition, research itself can be damaging. As noted by Ansara and Hegarty,^37^ some research continues to perpetuate pathologizing beliefs and to misgender participants from multiple angles.

The selection of the term “evidence map” is informative. Building on the work of Ahmed,^38^ and her approach to following multiple meanings of words and concepts, it is useful to be circumspect about the concept of evidence in relation to evidence-based practice, and about research as a form of evidence. One should be mindful of the implicit goals of empirical research, and question evidence as “evidence of what?,” and “evidence for what?.” In addition to providing data, the research articles in this review are themselves a form of evidence, documenting the actions and decisions of researchers and clinicians.

In speaking of evidence maps, we refer as well to evidence gaps. What do gaps mean in the context of research about trans people? The word gap suggests that something is absent. However, we should ask whether what is missing is something that should be there. What do these gaps hinder and what purpose might they also serve, and perhaps more importantly, whom do they serve? This analysis leads to larger questions about who and what gets studied, who makes these decisions, and what motivates researcher attention.

Critical Data Studies^39^ highlights the connections between “the spatial nature of data” and “the processes of data production and accumulation” (p. 1). Data visualizations such as maps are built on templates of those that have come before. In some ways, this map is no different. It mirrors a tradition of evidence mapping and borrows from longer standing frameworks related to social determinants of health and medical framing of experiences. Where this project is different is in the ways we consider the potential of digital evidence maps as living documents^40^ that can be leveraged to document previous ways of working and to “challenge the legacies of colonialism—to emphasize local knowledge and local control” (p. 422).^41^

In identifying future directions for research and knowledge synthesis, it is critical to engage trans communities and other stakeholders in local and global contexts to determine research priorities. Engagement is about more than participation: rather we advocate for the centering of trans people, and more specifically trans women of color.^42^ There are many excellent examples of these forms of engagement, including Marvellous Grounds: Queer of Colour Spaces in Toronto^43^ and the work of Reisner et al. at the Fenway Institute.^44^ These types of involvement will help to ensure that the knowledge that is produced is relevant to trans communities and to stakeholders such as policy-makers, health care providers, and educators. Within this study, we have taken the approach that it is better for people to be aware of the types of research that are being conducted. These insights make it clearer as to whose knowledge and perspectives are centered in this work, and it is more likely that trans communities and our allies will be in a position to benefit from existing research and hold researchers accountable as community awareness increases.

## Supplementary Material

Supplemental data

Supplemental data

Supplemental data

## Footnotes

^*^ The articles that were categorized within the intersectionalities study topic explicitly explored the impact of interacting or interlocking identities. For example, if the article was about trans women with disabilities, or trans people of color, the authors needed to address the ways these experiences intersected to produce specific structural dynamics, rather than exploring these as stand-alone topics.

## Abbreviations Used

DSD: disorders of sex development
LGBT2Q: lesbian, gay, bisexual, trans, Two-Spirit, and queer
MSM: men who have sex with men
PRISMA-P: Preferred Reporting Items for Systematic Review and Meta-Analysis Protocols
STI: sexually transmitted infection
