# Supplementary material for: Documenting Research with Transgender, Nonbinary, and Other Gender Diverse (Trans) Individuals and Communities: Introducing the Global Trans Research Evidence Map
Source: Transgend Health. 2019 Mar 1;4(1):68–80. doi: 10.1089/trgh.2018.0020 (PMC6400230; doi:10.1089/trgh.2018.0020)
Supplement: Supplemental data [file Supp_Data2.docx]

**Supplemental Data S2: Full List of Trans-Focused References**

Abdullah, M. A., Basharat, Z., Kamal, B., Sattar, N. Y., Hassan, Z. F., Jan, A. D., & Shafqat, A. (2012). Is social exclusion pushing the Pakistani hijras (transgenders) towards commercial sex work? A qualitative study. *BMC International Health and Human Rights*, *12*(32). doi:10.1186/1472-698X-12-32

Abelson, M. J. (2014). Dangerous privilege: Trans men, masculinities, and changing perceptions of safety. *Sociological Forum*, *29*(3), 549-570.

Adams, C., & Kumar, R. (2013). The effect of estrogen in a man with Parkinson’s disease and a review of its therapeutic potential. *International Journal of Neuroscience*, *123*(10), 741-742.

Adenuga, P., Summers, P., & Bergfeld, W. (2012). Hair regrowth in a male patient with extensive androgenetic alopecia on estrogen therapy. *Journal of the American Academy of Dermatology*, *67*(3), e121-e123.

Agrawal, N., Altiner, S., Mezitis-Nicholas, H. E., & Helbig, S. (2013). Silicone-induced granuloma after injection for cosmetic purposes: A rare entity of calcitriol-mediated hypercalcemia. *Case Reports in Medicine*, 807292. doi:[10.1155/2013/807292](http://dx.doi.org/10.1155/2013/807292)

Ahlin, H. B., Kolby, L., Elander, A., & Selvaggi, G. (2014). Improved results after implementation of the Ghent algorithm for subcutaneous mastectomy in female-to-male transsexuals. *Journal of Plastic Surgery and Hand Surgery*, *48*(6), 362-367.

Ahmadzad-Asl, M., Jalali, A.-M., Alavi, K., Naserbakht, M., Taban, M., Mohseninia-Omrani, K., & Eftekhar M. (2011). The epidemiology of transsexualism in Iran. *Journal of Gay & Lesbian Mental Health*, *15*(1), 83-93.

Ahmed, U., Yasin, G., & Umair, A. (2014). Factors affecting the social exclusion of eunuchs (Hijras) in Pakistan. *Mediterranean Journal of Social Sciences*, *5*(23), 2277-2284.

Ainsworth, T. A., & Spiegel, J. H. (2010). Quality of life of individuals with and without facial feminization surgery or gender reassignment surgery. *Quality of Life Research: An International Journal of Quality of Life Aspects of Treatment, Care and Rehabilitation*, *19*(7), 1019-1024.

Akhtar, H., Badshah, Y., Akhtar, S., Kanwal, N., Akhtar, M. N., Zaidi, N. U., & Qadri, I. (2012). Prevalence of human immunodeficiency virus infection among transgender men in Rawalpindi (Pakistan). *Virology Journal*, *9*(229). doi:10.1186/1743-422X-9-229

Alameddine, A. K., Alimov, V. K., Turner, Jr. G. S., & Deaton, D. W. (2011). Surgical pitfalls of excising an intramyocardial lipoma. *Journal of Thoracic and Cardiovascular Surgery*, *141*(2), 592-594.

Algars, M., Alanko, K., Santtila, P., & Sandnabba, N. K. (2012). Disordered eating and gender identity disorder: A qualitative study. *Eating Disorders*, *20*(4), 300-311.

Alhabshi, S. M. I., Ismail Z., & Arasaratnam, S. A. (2011). Primary non-Hodgkin B cell lymphoma in a man. *Iranian Journal of Radiology*, *8*(1), 39-41.

Altaf, A., Zahidie, A., & Agha, A. (2012). Comparing risk factors of HIV among hijra sex workers in Larkana and other cities of Pakistan: An analytical cross sectional study. *BMC Public Health*, *12*(1), 279-287.

Altman, K. (2012). Facial feminization surgery: Current state of the art. *International Journal of Oral and Maxillofacial Surgery*, *41*(8), 885-894.

Altomare, D. F., Scalera, I., Bettocchi, C., & Di Lena, M. (2013). Graciloplasty for recurrent recto-neovaginal fistula in a male-to-female transsexual. *Techniques in Coloproctology*, *17*(1), 107-109.

Amend, B., Seibold, J., Toomey, P., Stenzl, A., & Sievert, K. D. (2013). Surgical reconstruction for male-to-female sex reassignment. *European Urology*, *64*(1), 141-149.

Aminsharifi, A., Afsar, F., Jafari, M., & Tourchi, A. (2012). Removal of an entrapped large metallic dilator from the sigmoid neovagina in a male-to-female transsexual using a laparoscopic approach. *International Journal of Surgery Case Reports*, *3*(7), 266-268.

Amirian, I., Gogenur, I., & Rosenberg, J. (2011). Conservatively treated perforation of the neovagina in a male to female transsexual patient. *BMJ Case Reports*, bcr0820103241. doi:[10.1136/bcr.08.2010.3241](https://dx.doi.org/10.1136%2Fbcr.08.2010.3241)

Anderson, J. A. (2014). Pitch elevation in trangendered patients: Anterior glottic web formation assisted by temporary injection augmentation. *Journal of Voice*, *28*(6), 816-821.

Andrasik, M. P., Yoon, R., Mooney, J., Broder, G., Bolton, M., Votto, T., & Davis-Vogel, A. (2014). Exploring barriers and facilitators to participation of male-to-female transgender persons in preventive HIV vaccine clinical trials. *Prevention Science*, *15*(3), 268-276.

Andreazza, T. S., Costa, A. B., Massuda, R., Salvador, J., Silveira, E. M., Piccon, F., ... & Lobato, M. I. R. (2014). Discordant transsexualism in male monozygotic twins: Neuroanatomical and psychological differences. *Archives of Sexual Behavior*, *43*(2), 399-405.

Ansara, Y. G., & Hegarty, P. (2012). Cisgenderism in psychology: Pathologising and misgendering children from 1999 to 2008. *Psychology and Sexuality*, *3*(2), 137-160.

Antoszewski, B., Bratoś, R., Sitek, A., & Fijałkowska, M. (2012). Long-term results of breast reduction in female-to-male transsexuals. *Polski Przeglad Chirurgiczny/ Polish Journal of Surgery*, *84*(3), 144-151.

Aramburu, A. C., & Ballard-Reisch, D. (2013). Gender expression as a reflection of identity reformation in couple partners following disclosure of male-to-female transsexualism. *International Journal of Transgenderism*, *14*(2), 49-65.

Arsenault, N. (2012). A manifesto of living self-portraiture (identity, transformation, and performance). *Canadian Theatre Review, 150*, 64-69.

Asscheman, H., Giltay, E. J., Megens, J. A. J., De Ronde, W., Van Trotsenburg, M. A. A., & Gooren, L. J. G. (2011). A long-term follow-up study of mortality in transsexuals receiving treatment with cross-sex hormones. *European Journal of Endocrinology*, *164*(4), 635-642.

Auer, M. K., Fuss, J., Hohne, N., Stalla, G. K., & Sievers, C. (2014). Transgender transitioning and change of self-reported sexual orientation. *PloS One*, *9*(10), e110016. doi:[10.1371/journal.pone.0110016](https://doi.org/10.1371/journal.pone.0110016)

Auer, M. K., Fuss, J., Stalla, G. K., & Athanasoulia, A. P. (2013a). Twenty years of endocrinologic treatment in transsexualism: Analyzing the role of chromosomal analysis and hormonal profiling in the diagnostic work-up. *Fertility and Sterility*, *100*(4), 1103-1110.

Auer, M. K., Hohne, N., Bazarra-Castro, M. A., Pfister, H., Fuss, J., Stalla, G. K., … Ising M. (2013b). Psychopathological profiles in transsexuals and the challenge of their special status among the sexes. *PloS One*, *8*(10), e78469. doi:[10.1371/journal.pone.0078469](https://doi.org/10.1371/journal.pone.0078469)

Ayanian, S., & Irwig, M. S. (2013). Hypogonadism in a male-to-female transsexual with super obesity. *Andrologia*, *45*(4), 285-288.

Baba, T., Endo, T., Ikeda, K., Shimizu, A., Honnma, H., Ikeda, H., … Saito, T. (2011). Distinctive features of female-to-male transsexualism and prevalence of gender identity disorder in Japan. *Journal of Sexual Medicine*, *8*(6), 1686-1693.

Bailey, L., Ellis, S. J., & McNeil, J. (2014). Suicide risk in the UK trans population and the role of gender transition in decreasing suicidal ideation and suicide attempt. *Mental Health Review Journal*, *19*(4), 209-220.

Balgos, B., Gaillard, J. C., & Sanz, K. (2012). The warias of Indonesia in disaster risk reduction: The case of the 2010 Mt Merapi eruption in Indonesia. *Gender & Development*, *20*(2), 337-348.

Bandini, E., Fisher, A. D., Ricca, V., Ristori, J., Meriggiola, M. C., Jannini, E. A., … Maggi, M. (2011). Childhood maltreatment in subjects with male-to-female gender identity disorder. *International Journal of Impotence Research*, *23*(6), 276-285.

Baradkar, V., Samal, B., Mali, S. A., Kulkarni, K., & Shastri, J. (2011). Acanthamoeba on Sabouraud’s agar from a patient with keratitis. *Tropical Parasitology*, *1*(2), 141-142.

Baral, S. D., Poteat, T., Stromdahl, S., Wirtz, A. L., Guadamuz, T. E., & Beyrer, C. (2013). Worldwide burden of HIV in transgender women: A systematic review and meta-analysis. *The Lancet Infectious Diseases*, *13*(3), 214-222.

Barišić, J., Milosavljević, M., Duišin, D., Batinić, B., Vujović, S., & Milovanović, S. (2014). Assessment of self-perception of transsexual persons: Pilot study of 15 patients.  *Scientific World Journal*, 281326. doi:[10.1155/2014/281326](http://dx.doi.org/10.1155/2014/281326)

Barnes, T. G., Christodoulidou, M., Lucky, M. A., Singh, G., & Artioukh, D. Y. (2013). Pitfalls in the management of rectal cancer after male-to-female sex change procedure. *Colorectal Disease*, *15*(4), e199.

Barnett, J. T., & Johnson, C. W. (2013). We are all royalty: Narrative comparison of a drag queen and king. *Journal of Leisure Research*, *45*(5), 677-694.

Bauer, G. R., Redman, N., Bradley, K., & Scheim, A. I. (2013). Sexual health of trans men who are gay, bisexual, or who have sex with men: Results from Ontario, Canada. *International Journal of Transgenderism*, *14*(2), 66-74.

Bauer, G. R., Scheim, A. I., Deutsch, M. B., & Massarella, C. (2014). Reported emergency department avoidance, use, and experiences of transgender persons in Ontario, Canada: Results from a respondent-driven sampling survey. *Annals of Emergency Medicine*, *63*(6), 713-720.

Bauer, G. R., Travers, R., Scanlon, K., & Coleman, T. A. (2012). High heterogeneity of HIV-related sexual risk among transgender people in Ontario, Canada: A province-wide respondent-driven sampling survey. *BMC Public Health*, *12*(1), 292-303.

Bazargan, M., & Galvan, F. (2012). Perceived discrimination and depression among low-income Latina male-to-female transgender women. *BMC Public Health*, *12*(1), 663-670.

Beagan, B. L., De Souza, L., Godbout, C., Hamilton, L., MacLeod, J., Paynter, E., & Tobin, A. (2012). “This is the biggest thing you’ll ever do in your life”: Exploring the occupations of transgendered people. *Journal of Occupational Science*, *19*(3), 226-240.

Benotsch, E. G., Zimmerman, R., Cathers, L., McNulty, S., Pierce, J., Heck, T., … Snipes, D. (2013). Non-medical use of prescription drugs, polysubstance use, and mental health in transgender adults. *Drug and Alcohol Dependence*, *132*(1-2), 391-394.

Benson, K. E. (2013). Seeking support: Transgender client experiences with mental health services. *Journal of Feminist Family Therapy*, *25*(1), 17-40.

Bento, B. (2012). Sexuality and trans experiences: From the hospital to the bedroom. *Ciencia & Saude Coletiva*, *17*(10), 2655-2664.

Bentz, E. K., Pils, D., Bilban, M., Kaufmann, U., Hefler, L. A., Reinthaller, A., … Tempfer, C. B. (2010). Gene expression signatures of breast tissue before and after cross-sex hormone therapy in female-to-male transsexuals. *Fertility and Sterility*, *94*(7), 2688-2696.

Berkowitz, D., & Belgrave, L. L. (2010). “She works hard for the money”: Drag queens and the management of their contradictory status of celebrity and marginality. *Journal of Contemporary Ethnography*, *39*(2), 159-186.

Berry, M. G., Curtis, R., & Davies, D. (2012). Female-to-male transgender chest reconstruction: A large consecutive, single-surgeon experience. *Journal of Plastic, Reconstructive & Aesthetic Surgery: JPRAS*, *65*(6), 711-719.

Bethea, M. S., & McCollum, E. E. (2013). The disclosure experiences of male-to-female transgender individuals: A systems theory perspective. *Journal of Couple & Relationship Therapy*, *12*(2), 89-112.

Bhatta, D. N. (2014). HIV-related sexual risk behaviors among male-to-female transgender people in Nepal. *International Journal of Infectious Diseases*, *22*, 11-15.

Bianca, R., Mitidieri, E., Fusco, F., D’Aiuto, E., Grieco, P., Novellino, E., … Sorrentino, R. (2012). Endogenous urotensin II selectively modulates erectile function through eNOS. *PloS One*, *7*(2), e31019. doi:[10.1371/journal.pone.0031019](https://doi.org/10.1371/journal.pone.0031019)

Bith-Melander, P., Sheoran, B., Sheth, L., Bermudez, C., Drone, J., Wood, W., & Schroeder, K. (2010). Understanding sociocultural and psychological factors affecting transgender people of color in San Francisco. *JANAC: Journal of the Association of Nurses in AIDS Care*, *21*(3), 207-220.

Blackburn, M. V. (2014). (Re)writing one’s self as an activist across schools and sexual and gender identities: An investigation of the limits of LGBT-inclusive and queering discourses. *Journal of Language and Literacy Education*, *10*(1), 1-13.

Blanchard, R. (2010). The DSM diagnostic criteria for transvestic fetishism. *Archives of Sexual Behavior*, *39*(2), 363-372.

Blosnich, J. R., Brown, G. R., Shipherd, J. C., Kauth, M., Piegari, R. I., & Bossarte, R. M. (2013). Prevalence of gender identity disorder and suicide risk among transgender veterans utilizing veterans health administration care. *American Journal of Public Health*, *103*(10), e27-e32.

Blumer, M. L. C., Green, M. S., Knowles, S. J., & Williams, A. (2012). Shedding light on thirteen years of darkness: Content analysis of articles pertaining to transgender issues in marriage/couple and family therapy journals. *Journal of Marital & Family Therapy*, *38*(S1), 244-256.

Bockting, W., Miner, M., Romine, R. E., Swinburne, H. A., & Coleman, E. (2013). Stigma, mental health, and resilience in an online sample of the US transgender population. *American Journal of Public Health*, *103*(5), 943-951.

Bodoin, E. M., Byrd, C. T., & Adler, R. K. (2014). The clinical profile of the male-to-female transgender person of the 21st century. *Contemporary Issues in Communication Science & Disorders*, *41*, 39-54.

Bogliolo, S., Cassani, C., Babilonti, L., Gardella, B., Zanellini, F., Dominoni, M., … Spinillo, A. (2014). Robotic single-site surgery for female-to-male transsexuals: Preliminary experience. *Scientific World Journal*, 674579. doi:[10.1155/2014/674579](http://dx.doi.org/10.1155/2014/674579)

Bolger, A., Jones, T., Dunstan, D., & Lykins, A. (2014). Australian trans men: Development, sexuality, and mental health. *Australian Psychologist*, *49*(6), 395-402.

Boqun, X., Xiaonan, D., YuGui, C., Lingling, G., Xue, D., Gao, C., ... & Ma, Z.  (2013). Expression of SET protein in the ovaries of patients with polycystic ovary syndrome. *International Journal of Endocrinology*, 367956. doi:10.1155/2013/367956

Borg, M. B. J. (2011). Heist-ing the analyst’s penis (at gunpoint): Community enactment in the treatment of an FtM transgendered analysand. *International Journal of Transgenderism*, *13*(2), 77-90.

Boske, C. (2011). My name is Michelle: A real-life case to raise consciousness. *Journal of Cases in Educational Leadership*, *14*(2), 49-60.

Boza, C., & Nicholson, P. K. (2014). Gender-related victimization, perceived social support, and predictors of depression among transgender Australians. *International Journal of Transgenderism*, *15*(1), 35-52.

Bradford, J., Reisner, S. L., Honnold, J. A., & Xavier, J. (2013). Experiences of transgender-related discrimination and implications for health: Results from the Virginia Transgender Health Initiative Study. *American Journal of Public Health*, *103*(10), 1820-1829.

Brennan, J., Kuhns, L. M., Johnson, A. K., Belzer, M., Wilson, E. C., & Garofalo, R. (2012). Syndemic theory and HIV-related risk among young transgender women: The role of multiple, co-occurring health problems and social marginalization. *American Journal of Public Health*, *102*(9), 1751-1757.

Brewster, M. E., Velez, B., DeBlaere, C., & Bonnie, M. (2012). Transgender individuals’ workplace experiences: The applicability of sexual minority measures and models. *Journal of Counseling Psychology*, *59*(1), 60-70.

Brewster, M. E., Velez, B. L., Mennicke, A., & Tebbe, E. (2014). Voices from beyond: A thematic content analysis of transgender employees’ workplace experiences. *Psychology of Sexual Orientation and Gender Diversity*, *1*(2), 159-169.

Briones, M. R. S. A. (2011). Crossers at crossing: Narratives of work and aspirations of transgender informal workers in Los Baños, Laguna. *Philippine Quarterly of Culture and Society*, *39*(1), 1-26.

Brown, C., Dashjian, L. T., Acosta, T. J., Mueller, C. T., Kizer, B. E., & Trangsrud, H. B. (2012). The career experiences of male-to-female transsexuals. *The Counseling Psychologist*, *40*(6), 868-894.

Brown, C., Dashjian, L. T., Acosta, T. J., Mueller, C. T., Kizer, B. E., & Trangsrud, H. B. (2013). Learning from the life experiences of male-to-female transsexuals. *Journal of GLBT Family Studies*, *9*(2), 105-128.

Brown, G. R. (2010). Autocastration and autopenectomy as surgical self-treatment in incarcerated persons with gender identity disorder. *International Journal of Transgenderism*, *12*(1), 31-39.

Brown, G. R. (2014). Qualitative analysis of transgender inmates’ correspondence: Implications for Departments of Correction. *Journal of Correctional Health Care*, *20*(4), 334-342.

Browne, K., & Lim, J. (2010). Trans lives in the “gay capital of the UK.” *Gender, Place & Culture: A Journal of Feminist Geography*, *17*(5), 615-633.

Brunocilla, E., Soli, M., Franceschelli, A., Schiavina, R., Borghesi, M., Gentile, G., … Colombo, F. (2012). Radiological evaluation by magnetic resonance of the “new anatomy” of transsexual patients undergoing male to female sex reassignment surgery. *International Journal of Impotence Research*, *24*(5), 206-209.

Bucci, S., Mazzon, G., Liguori, G., Napoli, R., Pavan, N., Bormioli, S., … Trombetta, C. (2014). Neovaginal prolapse in male-to-female transsexuals: An 18-year-long experience. *BioMed Research International*, 240761. doi:[10.1155/2014/240761](http://dx.doi.org/10.1155/2014/240761)

Budge, S. L., Adelson, J. L., & Howard, K. A. S. (2013a). Anxiety and depression in transgender individuals: The roles of transition status, loss, social support, and coping. *Journal of Consulting and Clinical Psychology*, *81*(3), 545-557.

Budge, S. L., Katz-Wise, S. L., Tebbe, E. N., Howard, K. A. S., Schneider, C. L., & Rodriguez, A. (2013b). Transgender emotional and coping processes: Facilitative and avoidant coping throughout gender transitioning. *The Counseling Psychologist*, *41*(4), 601-647.

Budge, S. L., Rossman, H. K., & Howard, K. A. S. (2014). Coping and psychological distress among genderqueer individuals: The moderating effect of social support. *Journal of LGBT Issues in Counseling*, *8*(1), 95-117.

Budge, S. L., Tebbe, E. N., & Howard, K. A. S. (2010). The work experiences of transgender individuals: Negotiating the transition and career decision-making processes. *Journal of Counseling Psychology*, *57*(4), 377-397.

Bui, H. N., Schagen, S. E., Klink, D. T., Delemarre-van de Waal, H. A., Blankenstein, M. A., & Heijboer, A. C. (2013). Salivary testosterone in female-to-male transgender adolescents during treatment with intra-muscular injectable testosterone esters. *Steroids*, *78*(1), 91-95.

Burdge, B. J. (2014). Being true, whole, and strong: A phenomenology of transgenderism as a valued life experience. *Journal of Gay & Lesbian Social Services,* *26*(3), 355-382.

Burke, S. M., Cohen-Kettenis, P. T., Veltman, D. J., Klink, D. T., & Bakker, J. (2014a). Hypothalamic response to the chemo-signal androstadienone in gender dysphoric children and adolescents. *Frontiers in Endocrinology*, *5*, 60. doi: 10.3389/fendo.2014.00060

Burke, S. M., Menks, W. M., Cohen-Kettenis, P. T., Klink, D. T., & Bakker, J. (2014b). Click-evoked otoacoustic emissions in children and adolescents with gender identity disorder. *Archives of Sexual Behavior*, *43*(8), 1515-1523.

Busari, A. O. (2013). Bolstering self-esteem as intervention technique in the management of symptoms of gender identity disorder among adolescents. *Gender & Behaviour*, *11*(2), 5535-5545.

Caldarera, A., & Pfäfflin, F. (2011). Transsexualism and sex reassignment surgery in Italy. *International Journal of Transgenderism*, *13*(1), 26-36.

Camp, S., Cartwright, P., & Siddiqi, F. (2011). The prefabricated gracilis muscle flap with full-thickness skin graft and delay for urethral channel reconstruction. *Annals of Plastic Surgery*, *67*(1), 59-61.

Can, O. I., Demiroǧlu, Z., Köker, M., Ulaş, H., & Salaçin, S. (2011). Legal aspects of gender reassignment surgery in Turkey: A case report. *Indian Journal of Gender Studies*, *18*(1), 77-88.

Capitan, L., Simon, D., Kaye, K., & Tenorio, T. (2014). Facial feminization surgery: The forehead. Surgical techniques and analysis of results. *Plastic and Reconstructive Surgery*, *134*(4), 609-619.

Carella, S., Romanzi, A., Ciotti, M., & Onesti, M. G. (2013). Skin ulcer: A long-term complication after massive liquid silicone oil infiltration. *Aesthetic Plastic Surgery*, *37*(6), 1220-1224.

Carobene, M., Bolcic, F., Farias, M. S., Quarleri, J., & Avila, M. M. (2014). HIV, HBV, and HCV molecular epidemiology among trans (transvestites, transsexuals, and transgender) sex workers in Argentina. *Journal of Medical Virology*, *86*(1), 64-70.

Carrillo, B., Gomez-Gil, E., Rametti, G., Junque, C., Gomez, A., Karadi, K., … Guillamon, A. (2010). Cortical activation during mental rotation in male-to-female and female-to-male transsexuals under hormonal treatment. *Psychoneuroendocrinology*, *35*(8), 1213-1222.

Case, L. K., & Ramachandran, V. S. (2012). Alternating gender incongruity: A new neuropsychiatric syndrome providing insight into the dynamic plasticity of brain-sex. *Medical Hypotheses*, *78*(5), 626-631.

Caudwell, J. (2014). [Transgender] young men: Gendered subjectivities and the physically active body. *Sport, Education and Society*, *19*(4), 398-414.

Cebula, H., Pham, T. Q., Boyer, P., & Froelich, S. (2010). Regression of meningiomas after discontinuation of cyproterone acetate in a transsexual patient. *Acta Neurochirurgica*, *152*(11), 1955-1956.

Cerezo, A., Morales, A., Quintero, D., & Rothman, S. (2014). Trans migrations: Exploring life at the intersection of transgender identity and immigration. *Psychology of Sexual Orientation and Gender Diversity*, *1*(2), 170-180.

Cerwenka, S., Nieder, T. O., Briken, P., Cohen-Kettenis, P. T., Cuypere, G., Haraldsen, I. R. H., … Richter-Appelt, H. (2014a). Intimate partnerships and sexual health in gender-dysphoric individuals before the start of medical treatment. *International Journal of Sexual Health*, *26*(1), 52-65.

Cerwenka, S., Nieder, T. O., Cohen-Kettenis, P., De Cuypere, G., Haraldsen, I. R., Kreukels, B. P., & Richter-Appelt, H. (2014b). Sexual behavior of gender-dysphoric individuals before gender-confirming interventions: A European multicenter study. *Journal of Sex & Marital Therapy*, *40*(5), 457-471.

Chan, K. L., & Mok, C. C. (2013). Development of systemic lupus erythematosus in a male-to-female transsexual: The role of sex hormones revisited. *Lupus*, *22*(13), 1399-1402.

Chandra, P., Basra, S. S., Chen, T. C., & Tangpricha, V. (2010). Alterations in lipids and adipocyte hormones in female-to-male transsexuals. *International Journal of Endocrinology*, 945053. doi:[10.1155/2010/945053](http://dx.doi.org/10.1155/2010/945053)

Chang, H. L., & Chow, C. C. (2011). The treatment of fetishism in an adolescent with attention deficit hyperactivity disorder. *Chang Gung Medical Journal*, *34*(4), 440-443.

Chekir, C., Emi, Y., Arai, F., Kikuchi, Y., Sasaki, A., Matsuda, M., ... & Nakatsuka, M. (2012). Altered arterial stiffness in male‐to‐female transsexuals undergoing hormonal treatment. *Journal of Obstetrics and Gynaecology Research*, *38*(6), 932-940.

Chen, S., Mcfarland, W., Thompson, H. M., & Raymond, H. F. (2011). Transmen in San Francisco: What do we know from HIV test site data? *AIDS and Behavior*, *15*(3), 659-662.

Cho, S. W., & Jin, H. R. (2012). Feminization of the forehead in a transgender: Frontal sinus reshaping combined with brow lift and hairline lowering. *Aesthetic Plastic Surgery*, *36*(5), 1207-1210.

Church, H. A., O’Shea, D., & Lucey, J. V. (2014). Parent-child relationships in gender identity disorder. *Irish Journal of Medical Science*, *183*(2), 277-281.

Cohen, B., Parker, D., Lu, C. T., & Strahan, A. (2011). An unusual case of caecal volvulus. *ANZ Journal of Surgery*, *81*(12), 944-945.

Cohen-Kettenis, P. T., Schagen, S. E. E., Steensma, T. D., De Vries, A. L. C., & Delemarre-Van De Waal, H. A. (2011). Puberty suppression in a gender-dysphoric adolescent: A 22-year follow-up. *Archives of Sexual Behavior*, *40*(4), 843-847.

Colebunders, B., T’Sjoen, G., Weyers, S., & Monstrey, S. (2014). Hormonal and surgical treatment in trans-women with BRCA1 mutations: A controversial topic. *Journal of Sexual Medicine*, *11*(10), 2496-2499.

Colizzi, M., Costa, R., Pace, V., & Todarello, O. (2013). Hormonal treatment reduces psychobiological distress in gender identity disorder, independently of the attachment style. *Journal of Sexual Medicine*, *10*(12), 3049-3058.

Colizzi, M., Costa, R., & Todarello, O. (2014). Transsexual patients’ psychiatric comorbidity and positive effect of cross-sex hormonal treatment on mental health: Results from a longitudinal study. *Psychoneuroendocrinology*, *39*, 65-73.

Connell, C. (2010a). Doing, undoing, or redoing gender? Learning from the workplace experiences of transpeople. *Gender & Society*, *24*(1), 31-55.

Connell, R. (2010b). Two cans of paint: A transsexual life story, with reflections on gender change and history. *Sexualities*, *13*(1), 3-19.

Conron, K. J., Scott, G., Stowell, G. S., & Landers, S. J. (2012). Transgender health in Massachusetts: Results from a household probability sample of adults. *American Journal of Public Health*, *102*(1), 118-122.

Costa, E. M., & Mendonca, B. B. (2014). Clinical management of transsexual subjects. *Arquivos Brasileiros de Endocrinologia E Metabologia*, *58*(2), 188-196.

Costantino, A., Cerpolini, S., Alvisi, S., Morselli, P. G., Venturoli, S., & Meriggiola, M. C. (2013). A prospective study on sexual function and mood in female-to-male transsexuals during testosterone administration and after sex reassignment surgery. *Journal of Sex & Marital Therapy*, *39*(4), 321-335.

Cosyns, M., Van Borsel, J., Wierckx, K., Dedecker, D., Van de Peer, F., Daelman, T., … T’Sjoen, G. (2014). Voice in female-to-male transsexual persons after long-term androgen therapy. *The Laryngoscope*, *124*(6), 1409-1414.

Cotton, R. (2014). Supporting transgender students in schools. *British Journal of School Nursing*, *9*(3), 141-143.

Cousino, M. K., Davis, A., Ng, H., & Stancin, T. (2014). An emerging opportunity for pediatric psychologists: Our role in a multidisciplinary clinic for youth with gender dysphoria. *Clinical Practice in Pediatric Psychology*, *2*(4), 400-411.

Cregten-Escobar, P., Bouman, M. B., Buncamper, M. E., & Mullender, M. G. (2012). Subcutaneous mastectomy in female-to-male transsexuals: A retrospective cohort-analysis of 202 patients. *Journal of Sexual Medicine*, *9*(12), 3148-3153.

Cruz, T. M. (2014). Assessing access to care for transgender and gender nonconforming people: A consideration of diversity in combating discrimination. *Social Science & Medicine*, *110*, 65-73.

Cupisti, S., Giltay, E. J., Gooren, L. J., Kronawitter, D., Oppelt, P. G., Beckmann, M. W., … Mueller, A. (2010). The impact of testosterone administration to female-to-male transsexuals on insulin resistance and lipid parameters compared with women with polycystic ovary syndrome. *Fertility and Sterility*, *94*(7), 2647-2653.

d’Ythurbide, G., Kerrou, K., Brocheriou, I., & Hertig, A. (2012). Reactive amyloidosis complicated by end-stage renal disease 28 years after liquid silicone injection in the buttocks. *BMJ Case Reports*. doi:10.1136/bcr-2012-006803

da Silva, F. G., Filho, A. M., Damiao, R., & da Silva, E. A. (2011). Human acellular matrix graft of tunica albuginea for penile reconstruction. *Journal of Sexual Medicine*, *8*(11), 3196-3203.

Daniolos, P. T, & Telingator, C. J. (2013). Engendering identity. *Journal of the American Academy of Child & Adolescent Psychiatry*, *52*(12), 1245-1247.

Dargie, E., Blair, K. L, Pukall, C. F., & Coyle, S. M. (2014). Somewhere under the rainbow: Exploring the identities and experiences of trans persons. *Canadian Journal of Human Sexuality*, *23*(2), 60-74.

Dasgupta, T., Dasgupta, R., & Dwivedi, P. (2012). Somatic inkblots imagery in transsexual: A case study. *SIS Journal of Projective Psychology & Mental Health*, *19*(1), 61-65.

Davey, A., Bouman, W. P., Arcelus, J., & Meyer, C. (2014). Social support and psychological well-being in gender dysphoria: A comparison of patients with matched controls. *Journal of Sexual Medicine*, *11*(12), 2976-2985.

Davies, A., Bouman, W. P., Richards, C., Barrett, J., Ahmad, S., Baker, K., … Stradins, L. (2013). Patient satisfaction with gender identity clinic services in the United Kingdom. *Sexual and Relationship Therapy*, *28*(4), 400-418.

Davis, S. A., & Meier, S. C. (2014). Effects of testosterone treatment and chest reconstruction surgery on mental health and sexuality in female-to-male transgender people. *International Journal of Sexual Health*, *26*(2), 113-128.

de Lind van Wijngaarden, J. W., Schunter, B. T., & Iqbal, Q. (2013). Sexual abuse, social stigma and HIV vulnerability among young feminised men in Lahore and Karachi, Pakistan. *Culture, Health & Sexuality*, *15*(1), 73-84.

de Ronde, W., Vogel, S., Bui, H. N., & Heijboer, A. C. (2011). Reduction in 24-hour plasma testosterone levels in subjects who showered 15 or 30 minutes after application of testosterone gel. *Pharmacotherapy*, *31*(3), 248-252.

De Santis, J. P., Martin, C. W., & Lester, A. (2010). An educational program on HIV prevention for male-to-female transgender women in South Miami Beach, Florida. *JANAC: Journal of the Association of Nurses in AIDS Care*, *21*(3), 265-271.

de Vries, A. L., Doreleijers, T. A., Steensma, T. D., & Cohen-Kettenis, P. T. (2011a). Psychiatric comorbidity in gender dysphoric adolescents. *Journal of Child Psychology and Psychiatry, and Allied Disciplines*, *52*(11), 1195-1202.

de Vries, A. L., Kreukels, B. P., Steensma T. D., Doreleijers T. A., & Cohen-Kettenis P. T. (2011b). Comparing adult and adolescent transsexuals: An MMPI-2 and MMPI-A study. *Psychiatry Research*, *186*(2/3), 414-418.

de Vries, A. L., McGuire, J. K., Steensma, T. D., Wagenaar, E. C., Doreleijers, T. A., & Cohen-Kettenis, P. T. (2014). Young adult psychological outcome after puberty suppression and gender reassignment. *Pediatrics*, *134*(4), 696-704.

de Vries, A. L., Noens, I. L., Cohen-Kettenis, P. T., van Berckelaer-Onnes, I. A., & Doreleijers, T. A. (2010). Autism spectrum disorders in gender dysphoric children and adolescents. *Journal of Autism and Developmental Disorders*, *40*(8), 930-936.

de Vries, A. L., Steensma, T. D., Doreleijers, T. A., & Cohen-Kettenis, P. T. (2011c). Puberty suppression in adolescents with gender identity disorder: A prospective follow-up study. *Journal of Sexual Medicine*, *8*(8), 2276-2283.

de Vries, K. M. (2012). Intersectional identities and conceptions of the self: The experience of transgender people. *Symbolic Interaction*, *35*(1), 49-67.

Deipolyi, A. R., Han, S. J., & Parsa, A. T. (2010). Development of a symptomatic intracranial meningioma in a male-to-female transsexual after initiation of hormone therapy. *Journal of Clinical Neuroscience: Official Journal of the Neurosurgical Society of Australasia*, *17*(10), 1324-1326.

Deliktas, H., Ozcan, O., Cullu, N., & Erdogan, O. (2014). Neovaginal perforation following sexual intercourse in a transsexual patient. *BMC Research Notes*, *7*(797). doi:10.1186/1756-0500-7-797

Dempf, R., & Eckert, A. W. (2010). Contouring the forehead and rhinoplasty in the feminization of the face in male-to-female transsexuals. *Journal of Cranio-Maxillofacial Surgery*, *38*(6), 416-422.

Dessy, L. A., Mazzocchi, M., Corrias, F., Ceccarelli, S., Marchese, C., & Scuderi, N. (2014). The use of cultured autologous oral epithelial cells for vaginoplasty in male-to-female transsexuals: A feasibility, safety, and advantageousness clinical pilot study. *Plastic and Reconstructive Surgery*, *133*(1), 158-161.

Devereaux, E. (2010). Doctor Alan Hart: X-ray vision in the archive. *Australian Feminist Studies*, *25*(64), 175-187.

Dhand, A., & Dhaliwal, G. (2010). Examining patient conceptions: A case of metastatic breast cancer in an African American male to female transgender patient. *Journal of General Internal Medicine*, *25*(2), 158-161.

Dhejne, C., Lichtenstein, P., Boman, M., Johansson, A. L., Langstrom, N., & Landen, M. (2011). Long-term follow-up of transsexual persons undergoing sex reassignment surgery: Cohort study in Sweden. *PloS One*, *6*(2), e16885. doi:[10.1371/journal.pone.0016885](https://doi.org/10.1371/journal.pone.0016885)

Dhejne, C., Öberg, K., Arver, S., & Landén, M. (2014). An analysis of all applications for sex reassignment surgery in Sweden, 1960-2010: Prevalence, incidence, and regrets. *Archives of Sexual Behavior*, *43*(8), 1535-1545.

Dhillon, R., Bastiampillai, T., Krishnan, S., Opray, N., & Tibrewal, P. (2011). Transgender late onset psychosis: The role of sex hormones. *Australian & New Zealand Journal of Psychiatry*, *45*(7), 595.

Di Ceglie, D., Skagerberg, E., Baron-Cohen, S., & Auyeung, B. (2014). Empathising and systemising in adolescents with gender dysphoria. *Opticon1826*, (16), 1-8.

Dickerson, E. M., Jones, P., Wilkins, D., Regnier, J., & Prahlow, J. A. (2013). Complicated suicide versus autoeroticism?: A case involving multiple drugs and a porta-potty. *The American Journal of Forensic Medicine and Pathology*, *34*(1), 29-33.

Dickey, L. M., Burnes, T. R., & Singh, A. A. (2012). Sexual identity development of female-to-male transgender individuals: A grounded theory inquiry. *Journal of LGBT Issues in Counseling*, *6*(2), 118-138.

Dietert, M., & Dentice, D. (2013). Growing up trans: Socialization and the gender binary. *Journal of GLBT Family Studies*, *9*(1), 24-42.

Dispenza, F., Watson, L. B., Chung, Y. B., & Brack, G. (2012). Experience of career-related discrimination for female-to-male transgender persons: A qualitative study. *The Career Development Quarterly*, *60*(1), 65-81.

Djordjevic, M. L., & Bizic, M. R. (2013). Comparison of two different methods for urethral lengthening in female to male (metoidioplasty) surgery. *Journal of Sexual Medicine*, *10*(5), 1431-1438.

Doan, P. L. (2010). The tyranny of gendered spaces- Reflections from beyond the gender dichotomy. *Gender, Place and Culture*, *17*(5), 635-654.

Doorduin, T., & van Berlo, W. (2014). Trans people’s experience of sexuality in the Netherlands: A pilot study. *Journal of Homosexuality*, *61*(5), 654-672.

dos Ramos Farías, M. S., Picconi, M. A., Garcia, M. N., González, J. V., Basiletti, J., Pando M. D. L. Á., & Ávila, M. M. (2011). Human papilloma virus genotype diversity of anal infection among trans (male to female transvestites, transsexuals or transgender) sex workers in Argentina. *Journal of Clinical Virology*, *51*(2), 96-99.

Dowshen, N., Forke, C. M., Johnson, A. K., Kuhns, L. M., Rubin, D., & Garofalo, R. (2011). Religiosity as a protective factor against HIV risk among young transgender women. *Journal of Adolescent Health*, *48*(4), 410-414.

du Preez, H. M. (2012). Dr James Barry (1789-1865): The Edinburgh years. *Journal of the Royal College of Physicians of Edinburgh*, *42*(3), 258-265.

Dubois, L. Z. (2012). Associations between transition-specific stress experience, nocturnal decline in ambulatory blood pressure, and C-reactive protein levels among transgender men. *American Journal of Human Biology*, *24*(1), 52-61.

Dugan, J. P., Kusel, M. L., & Simounet, D. M. (2012). Transgender college students: An exploratory study of perceptions, engagement, and educational outcomes. *Journal of College Student Development*, *53*(5), 719-736.

Duisin, D., Batinic, B., Barisic, J., Djordjevic, M. L., Vujovic, S., & Bizic, M. (2014). Personality disorders in persons with gender identity disorder. *Scientific World Journal*, 809058. doi:[10.1155/2014/809058](http://dx.doi.org/10.1155/2014/809058)

Dziengel, L. (2014). Renaming, reclaiming, renewing the self: Intersections of gender, identity, and health care. *Affilia: Journal of Women & Social Work*, *29*(1), 105-110.

Ecklund, K. (2012). Intersectionality of identity in children: A case study. *Professional Psychology: Research & Practice*, *43*(3), 256-264.

Edelman, E. A. (2011). “This area has been declared a prostitution free zone”: Discursive formations of space, the state, and trans “sex worker” bodies. *Journal of Homosexuality*, *58*(6-7), 848-864.

Edelman, E. A., & Zimman, L. (2014). Boycunts and bonus holes: Trans men’s bodies, neoliberalism, and the sexual productivity of genitals. *Journal of Homosexuality*, *61*(5), 673-690.

Edwards-Leeper, L., & Spack, N. P. (2012). Psychological evaluation and medical treatment of transgender youth in an interdisciplinary “Gender Management Service” (GeMS) in a major pediatric center. *Journal of Homosexuality*, *59*(3), 321-336.

Effrig, J. C., Bieschke, K. J., & Locke, B. D. (2011). Examining victimization and psychological distress in transgender college students. *Journal of College Counseling*, *14*(2), 143-157.

Ehrensaft, D. (2010). “I’m a Prius”: A child case of a gender/ethnic hybrid. *Journal of Gay & Lesbian Mental Health*, *15*(1), 46-57.

Ehrensaft, D. (2013). Look, mom, I’m a boy- Don’t tell anyone I was a girl. *Journal of LGBT Youth*, *10*(1-2), 9-28.

Ehsanzadeh, P., Raza, S., & Haq, Z. (2014). A new perspective on gender dysphoria and repetitive sex reassignment surgeries: A case report. *The Primary Care Companion for CNS Disorders*, *16*(2). doi:[10.4088/PCC.13l01608](https://dx.doi.org/10.4088%2FPCC.13l01608)

Eisner, S. (2012). Love, rage and the occupation: Bisexual politics in Israel/Palestine. *Journal of Bisexuality*, *12*(1), 80-137.

El Muayed, M., Costas, A. A., & Pick, A. J. (2010). 1,25-dihydroxyvitamin D-mediated hypercalcemia in oleogranulomatous mastitis (paraffinoma), ameliorated by glucocorticoid administration. *Endocrine Practice*, *16*(1), 102-106.

Elamin, M. B., Garcia, M. Z., Murad, M. H., Erwin, P. J., & Montori, V. M. (2010). Effect of sex steroid use on cardiovascular risk in transsexual individuals: A systematic review and meta-analyses. *Clinical Endocrinology*, *72*(1), 1-10.

Elaut, E., Bogaert, V., De Cuypere, G., Weyers, S., Gijs, L., Kaufman, J. M., & T’Sjoen, G. (2010). Contribution of androgen receptor sensitivity to the relation between testosterone and sexual desire: An exploration in male-to-female transsexuals. *Journal of Endocrinological Investigation*, *33*(1), 37-41.

Ellis, S. J., McNeil, J., & Bailey L. (2014). Gender, stage of transition and situational avoidance: A UK study of trans people’s experiences. *Sexual and Relationship Therapy*, *29*(3), 351-364.

Erich, S., Tittsworth, J., & Kersten, A. S. (2010a). An examination and comparison of transsexuals of color and their white counterparts regarding personal well-being and support networks. *Journal of GLBT Family Studies*, *6*(1), 25-39.

Erich, S., Tittsworth, J., Meier, S. L. C., & Lerman, T. (2010b). Transsexuals of color: Perceptions of discrimination based on transsexual status and race/ethnicity status. *Journal of GLBT Family Studies*, *6*(3), 294-314.

Ertemi, H., Mumtaz, F. H., Howie, A. J., Mikhailidis, D. P., & Thompson, C. S. (2011). Effect of angiotensin II and its receptor antagonists on human corpus cavernous contractility and oxidative stress: Modulation of nitric oxide mediated relaxation. *The Journal of Urology*, *185*(6), 2414-2420.

Esteva de Antonio, I. E., & Gomez-Gil, E. (2013). Coordination of healthcare for transsexual persons: A multidisciplinary approach. *Current Opinion in Endocrinology, Diabetes, and Obesity*, *20*(6), 585-591.

Ettner, R., Ettner, F., & White, T. (2012). Secrecy and the pathogenesis of hypertension. *International Journal of Family Medicine*, 492718. doi:[10.1155/2012/492718](http://dx.doi.org/10.1155/2012/492718)

Ewan, L. A., Middleman, A. B., & Feldmann, J. (2014). Treatment of anorexia nervosa in the context of transsexuality: A case report. *International Journal of Eating Disorders*, *47*(1), 112-115.

Fabbre, V. D. (2014). Gender transitions in later life: The significance of time in queer aging. *Journal of Gerontological Social Work*, *57*(2-4), 161-175.

Fabbri, R., Vicenti, R., Macciocca, M., Pasquinelli, G., Paradisi, R., Battaglia, C., … Venturoli, S. (2014). Good preservation of stromal cells and no apoptosis in human ovarian tissue after vitrification. *BioMed Research International*, 673537. doi:[10.1155/2014/673537](http://dx.doi.org/10.1155/2014/673537)

Faccini, L. (2010a). Lost in the shadow of the crowd: Will I be healed? Choosing treatment targets and approaches. *Sexuality and Disability*, *28*(2), 119-128.

Faccini, L. (2010b). Treatment for a person with intellectual disability, co-morbid clinical disorders and sex offenses. *Sexuality and Disability*, *28*(2), 129-139.

Faccini, L., & Saide, M. A. (2012). “Can you breathe?” Autoerotic asphyxiation and asphxiophilia in a person with an intellectual disability and sex offending. *Sexuality and Disability*, *30*(1), 97-101.

Faccio, E., Bordin, E., & Cipolletta, S. (2013). Transsexual parenthood and new role assumptions. *Culture, Health and Sexuality*, *15*(9), 1055-1070.

Fallon, S. (2012). Sex, gender, and the theatre of self: Acting theory in (Gestalt) psychotherapy with a transsexual client. *Gestalt Review*, *16*(2), 162-180.

Feldman, J., Romine, R. S., & Bockting, W. O. (2014a). HIV risk behaviors in the U.S. transgender population: Prevalence and predictors in a large internet sample. *Journal of Homosexuality*, *61*(11), 1558-1588.

Feldman, J., & Spencer, K. (2014b). Gender dysphoria in a 39-year-old man. *CMAJ: Canadian Medical Association Journal*, *186*(1), 49-50.

Fernandes, H. M., Manolitsas, T. P., & Jobling, T. W. (2014). Carcinoma of the neovagina after male-to-female reassignment. *Journal of Lower Genital Tract Disease*, *18*(2), E43-E45.

Fernández, R., Esteva, I., Gómez-Gil, E., Rumbo, T., Almaraz, M. C., Roda, E., … Pásaro, E. (2014a). Association study of ERβ, AR, and CYP19A1 genes and MtF transsexualism. *Journal of Sexual Medicine*, *11*(12), 2986-2994.

Fernández, R., Esteva, I., Gómez-Gil, E., Rumbo, T., Almaraz, M. C., Roda, E., … Pásaro, E. (2014b). The (CA)n polymorphism of ERβ gene is associated with FtM transsexualism. *Journal of Sexual Medicine*, *11*(3), 720-728.

Ferron, P., Young, S., Boulanger, C., Rodriguez, A., & Moreno, J. (2010). Integrated care of an aging HIV-infected male-to-female transgender patient. *JANAC: Journal of the Association of Nurses in AIDS Care*, *21*(3), 278-282.

Fink, M., & Miller, Q. (2014). Trans media moments: Tumblr, 2011-2013. *Television and New Media*, *15*(7), 611-626.

Finkenauer, S., Sherratt, J., Marlow, J., & Brodey, A. (2012). When injustice gets old: A systematic review of trans aging. *Journal of Gay & Lesbian Social Services*, *24*(4), 311-330.

Firth, M. T. (2014). Childhood abuse and depressive vulnerability in clients with gender dysphoria. *Counselling & Psychotherapy Research*, *14*(4), 297-305.

Fischer, E. M., Patsch, J., Muschitz, C., Becker, S., & Resch, H. (2011). Severe osteoporosis with multiple vertebral fractures after gender reassignment therapy- Is it male or female osteoporosis? *Gynecological Endocrinology*, *27*(5), 341-344.

Fisher, A. D., Bandini, E., Casale, H., Ferruccio, N., Meriggiola, M. C., Gualerzi, A., … Maggi, M. (2013). Sociodemographic and clinical features of gender identity disorder: An Italian multicentric evaluation. *Journal of Sexual Medicine*, *10*(2), 408-419.

Fisher, A. D., Bandini, E., Ricca, V., Ferruccio, N., Corona, G., Meriggiola, M. C., … Maggi, M. (2010). Dimensional profiles of male to female gender identity disorder: An exploratory research. *Journal of Sexual Medicine*, *7*(7), 2487-2498.

Fisher, A. D., Castellini, G., Bandini, E., Casale, H., Fanni, E., Benni, L., … Rellini, A. H. (2014). Cross-sex hormonal treatment and body uneasiness in individuals with gender dysphoria. *Journal of Sexual Medicine*, *11*(3), 709-719.

Fletcher, J. B., Kisler, K. A., & Reback, C. J. (2014). Housing status and HIV risk behaviors among transgender women in Los Angeles. *Archives of Sexual Behavior*, *43*(8), 1651-1661.

Flor-Henry, P. (2010). EEG analysis of male to female transsexuals: Discriminant function and source analysis. *Clinical EEG and Neuroscience*, *41*(4), 219-222.

Fontanari, A.-M. V., Andreazza, T., Costa, A. B., Salvador, J., Koff, W. J., Aguiar, B., … Lobato, M. I. R. (2013). Serum concentrations of brain-derived neurotrophic factor in patients with gender identity disorder. *Journal of Psychiatric Research*, *47*(10), 1546-1548.

Francis, D. (2014). “You must be thinking what a lesbian man teacher is doing in a nice place like Dipane Letsie School?”: Enacting, negotiating and reproducing dominant understandings of gender in a rural school in the Free State, South Africa. *Gender & Education*, *26*(5), 539-552.

Furuhashi, T. (2011). Biological male “Gender Identity Disorder” is composed of essentially distinguishable core and periphery groups. *Ethical Human Psychology and Psychiatry*, *13*(1), 64-75.

Gabrielli, E., Ferraioli, G., Ferraris, L., Riva, A., Galii, M., Filice, C., & Gervasoni, C. (2010). Enfuvirtide administration in HIV-positive transgender patient with soft tissue augmentation: US evaluation. *New Microbiologica*, *33*(3), 263-265.

Galupo, M. P., Bauerband, L. A., Gonzalez, K. A., Hagen, D. B., Hether, S. D., & Krum, T. E. (2014a). Transgender friendship experiences: Benefits and barriers of friendships across gender identity and sexual orientation. *Feminism & Psychology*, *24*(2), 193-215.

Galupo, M. P., Henise, S. B., & Davis, K. S. (2014b). Transgender microaggressions in the context of friendship: Patterns of experience across friends’ sexual orientation and gender identity. *Psychology of Sexual Orientation and Gender Diversity*, *1*(4), 461-470.

Galupo, M. P., Krum, T. E., Hagen, D. B., Gonzalez, K. A., & Bauerband, L. A. (2014c). Disclosure of transgender identity and status in the context of friendship. *Journal of LGBT Issues in Counseling*, *8*(1), 25-42.

Ganor, Y., Zhou, Z., Bodo, J., Tudor, D., Leibowitch, J., Mathez, D., … Bomsel, M. (2013). The adult penile urethra is a novel entry site for HIV-1 that preferentially targets resident urethral macrophages. *Mucosal Immunology*, *6*(4), 776-786.

Garaffa, G., Christopher, N. A., & Ralph, D. J. (2010a). Total phallic reconstruction in female-to-male transsexuals. *European Urology*, *57*(4), 715-722.

Garaffa, G., Ralph, D. J., & Christopher, N. (2010b). Total urethral construction with the radial artery-based forearm free flap in the transsexual. *BJU International*, *106*(8), 1206-1210.

Garcia, M. M., Christopher, N. A., De Luca, F., Spilotros, M., & Ralph, D. J. (2014). Overall satisfaction, sexual function, and the durability of neophallus dimensions following staged female to male genital gender confirming surgery: The Institute of Urology, London U.K. experience. *Translational Andrology and Urology*, *3*(2), 156-162.

Garcia, M., & Lehman, Y. (2011). Issues concerning the informality and outdoor sex work performed by travestis in Sao Paulo, Brazil. *Archives of Sexual Behavior*, *40*(6), 1211-1221.

Garcia-Malpartida, K., Martin-Gorgojo, A., Rocha, M., Gomez-Balaguer, M., & Hernandez-Mijares, A. (2010). Prolactinoma induced by estrogen and cyproterone acetate in a male-to-female transsexual. *Fertility & Sterility*, *94*(3), 1097.e13-1097.e15.

Garofalo, R., Johnson, A. K., Kuhns, L. M., Cotten, C., Joseph, H., & Margolis, A. (2012). Life skills: Evaluation of a theory-driven behavioral HIV prevention intervention for young transgender women. *Journal of Urban Health*, *89*(3), 419-431.

Gelfer, M. P., & Tice, R. M. (2013a). Perceptual and acoustic outcomes of voice therapy for male-to-female transgender individuals immediately after therapy and 15 months later. *Journal of Voice*, *27*(3), 335-347.

Gelfer, M. P., & Van Dong, B. R. (2013b). A preliminary study on the use of vocal function exercises to improve voice in male-to-female transgender clients. *Journal of Voice*, *27*(3), 321-334.

Gervasoni, C., Zanini, F., Gabrielli, E., Merli, S., Riva, A., & Galli, M. (2011). Tubercular gluteus abscesses: A return to the early 20th century or a consequence of new, unprecedented behaviors? *Clinical Infectious Diseases: An Official Publication of the Infectious Diseases Society of America*, *52*(8), 1082-1083.

Giami, A., & Beaubatie, E. (2014). Gender identification and sex reassignment surgery in the trans population: A survey study in France. *Archives of Sexual Behavior*, *43*(8), 1491-1501.

Giami, A., & Le Bail, J. (2011). HIV infection and STI in the trans population: A critical review. *Revue d’Epidemiologie et de Sante Publique*, *59*(4), 259-268.

Godoy, M. C. B., Nonaka, D., Lowy, J., & Ko, J. P. (2010). Ground-glass centrilobular nodules on multidetector CT scan. *CHEST*, *138*(2), 427-433.

Goldblum, P., Testa, R. J., Pflum, S., Hendricks, M. L., Bradford, J., & Bongar, B. (2012). The relationship between gender-based victimization and suicide attempts in transgender people. *Professional Psychology: Research and Practice*, *43*(5), 468-475.

Golub, S. A., Walker, J. J., Longmire-Avital, B., Bimbi, D. S., & Parsons, J. T. (2010). The role of religiosity, social support, and stress-related growth in protecting against HIV risk among transgender women. *Journal of Health Psychology*, *15*(8), 1135-1144.

Gómez-Gil, E., Esteva, I., Almaraz, M. C., Pasaro, E., Segovia, S., & Guillamon, A. (2010). Familiality of gender identity disorder in non-twin siblings. *Archives of Sexual Behavior*, *39*(2), 546-552.

Gomez-Gil, E., Esteva, I., Carrasco, R., Almaraz, M. C., Pasaro, E., Salamero, M., & Guillamon, A. (2011). Birth order and ratio of brothers to sisters in Spanish transsexuals. *Archives of Sexual Behavior*, *40*(3), 505-510.

Gomez-Gil, E., Gomez, A., Canizares, S., Guillamon, A., Rametti, G., Esteva, I., … Salamero-Baro, M. (2012a). Clinical utility of the Bem Sex Role Inventory (BSRI) in the Spanish transsexual and nontranssexual population. *Journal of Personality Assessment*, *94*(3), 304-309.

Gómez-Gil, E., Gutiérrez, F., Cañizares, S., Zubiaurre-Elorza, L., Monràs, M., de Antonio, I. E. … Guillamón A. (2013). Temperament and character in transsexuals. *Psychiatry Research*, *210*(3), 969-974.

Gómez-Gil, E., Zubiaurre-Elorza, L., de Antonio, I. E., Guillamon, A., & Salamero, M. (2014). Determinants of quality of life in Spanish transsexuals attending a gender unit before genital sex reassignment surgery. *Quality of Life Research*, *23*(2), 671-678.

Gómez-Gil, E., Zubiaurre-Elorza, L., Esteva, I., Guillamon, A., Godás, T., Almaraz, M. C., … Salamero M. (2012b). Hormone-treated transsexuals report less social distress, anxiety and depression. *Psychoneuroendocrinology*, *37*(5), 662-670.

Gonzalez, C. A., Bockting, W. O., Beckman, L. J., & Duran, R. E. (2012). Agentic and communal personality traits: Their associations with depression and resilience among transgender women. *Sex Roles*, *67*(9-10), 528-543.

Goodrich, K. M. (2012). Lived experiences of college-age transsexual individuals. *Journal of College Counseling*, *15*(3), 215-232.

Gooren, L., & Morgentaler, A. (2014). Prostate cancer incidence in orchidectomised male-to-female transsexual persons treated with oestrogens. *Andrologia*, *46*(10), 1156-1160.

Gooren, L. J., Sungkaew, T., & Giltay, E. J. (2013a). Exploration of functional health, mental well-being and cross-sex hormone use in a sample of Thai male-to-female transgendered persons (kathoeys). *Asian Journal of Andrology*, *15*(2), 280-285.

Gooren, L. J., van Trotsenburg, M. A. A., Giltay, E. J., & van Diest, P. J. (2013b). Breast cancer development in transsexual subjects receiving cross-sex hormone treatment. *Journal of Sexual Medicine*, *10*(12), 3129-3134.

Gorin-Lazard, A., Baumstarck, K., Boyer, L., Maquigneau, A., Gebleux, S., Penochet, J. C., … Bonierbale, M. (2012). Is hormonal therapy associated with better quality of life in transsexuals? A cross-sectional study. *Journal of Sexual Medicine*, *9*(2), 531-541.

Gorin-Lazard, A., Baumstarck, K., Boyer, L., Maquigneau, A., Penochet, J. C., Pringuey, D., … Auquier, P. (2013). Hormonal therapy is associated with better self-esteem, mood, and quality of life in transsexuals. *Journal of Nervous and Mental Disease*, *201*(11), 996-1000.

Govier, E., Diamond, M., Wolowiec, T., & Slade, C. (2010). Dichotic listening, handedness, brain organization, and transsexuality. *International Journal of Transgenderism*, *12*(3), 144-154.

Gower, K., & Ritter, B. A. (2010). Not a pronoun: A transgender’s professional journey. *The CASE Journal, 7*(1), 6-11.

Goyal, S., Deb, K. S., Elawadhi, D., & Kaw, N. (2014). Substance abuse as a way of life in marginalized gender identity disorder: A case report with review of Indian literature. *Asian Journal of Psychiatry*, *12*, 160-162.

Graham, L. F. (2014a). Navigating community institutions: Black transgender women’s experiences in schools, the criminal justice system, and churches. *Sexuality Research & Social Policy*, *11*(4), 274-287.

Graham, L. F., Crissman, H. P., Tocco, J., Hughes, L. A., Snow, R. C., & Padilla, M. B. (2014b). Interpersonal relationships and social support in transitioning narratives of Black transgender women in Detroit. *International Journal of Transgenderism*, *15*(2), 100-113.

Grant, P., Lipscomb, D., & Edgell, H. (2010). Emphysematous pelonephritis in a non-diabetic post-operative transsexual patient. *Acute Medicine*, *9*(1), 30-33.

Grossman, A. H., D’Augelli, A. R., & Frank, J. A. (2011). Aspects of psychological resilience among transgender youth. *Journal of LGBT Youth*, *8*(2), 103-115.

Grynberg, M., Fanchin, R., Dubost, G., Colau, J. C., Bremont-Weil, C., Frydman, R., & Ayoubi, J. M. (2010). Histology of genital tract and breast tissue after long-term testosterone administration in a female-to-male transsexual population. *Reproductive Biomedicine Online*, *20*(4), 553-558.

Guadamuz, T. E., Wimonsate, W., Varangrat, A., Phanuphak, P., Jommaroeng, R., McNicholl, J. M., … van Griensven, F. (2011). HIV prevalence, risk behavior, hormone use and surgical history among transgender persons in Thailand. *AIDS and Behavior*, *15*(3), 650-658.

Gupta, D., Elwadhi, D., Mehta, M., & Kaw, N. (2012). Psycho-social functioning in an individual with gender identity disorder. *Psychological Studies*, *57*(3), 269-272.

Guzman-Parra, J., Paulino-Matos, P., de Diego-Otero, Y., Perez-Costillas, L., Villena-Jimena, A., … Bergero-Miguel, T. (2014). Substance use and social anxiety in transsexual individuals. *Journal of Dual Diagnosis*, *10*(3), 162-167.

Hagen, D B., & Galupo, M. P. (2014). Trans* individuals’ experiences of gendered language with health care providers: Recommendations for practitioners. *International Journal of Transgenderism*, *15*(1), 16-34.

Hahn, A., Kranz, G. S., Kublbock, M., Kaufmann, U., Ganger, S., Hummer, A., … Lanzenberger, R. (2014). Structural connectivity networks of transgender people. *Cerebral Cortex, 25*(10), 3527-3534.

Haines, B. A., Ajayi, A. A., & Boyd, H. (2014). Making trans parents visible: Intersectionality of trans and parenting identities. *Feminism & Psychology*, *24*(2), 238-247.

Hakeem, A. (2012). Psychotherapy for gender identity disorders. *Advances in Psychiatric Treatment*, *18*(1), 17-24.

Hamdan, A. L. (2012). Cricothyroid approximation using a silastic sheath: A new approach. *Middle East Journal of Anaesthesiology*, *21*(6), 909-912.

Hancock, A. B., & Garabedian, L. M. (2013). Transgender voice and communication treatment: A retrospective chart review of 25 cases. *International Journal of Language & Communication Disorders*, *48*(1), 54-65.

Hancock, A., & Helenius, L. (2012). Adolescent male-to-female transgender voice and communication therapy. *Journal of Communication Disorders*, *45*(5), 313-324.

Hansen-Reid, M. (2011). Samoan Fa’afafine- Navigating the New Zealand prison environment: A single case study. *Sexual Abuse in Australia and New Zealand*, *3*(1), 4-9.

Hariri, L. P., Gaissert, H. A., Brown, R., Ciaranello, A., Greene, R. E., Selig, M. K., & Kradin, R. L. (2012). Progressive granulomatous pneumonitis in response to cosmetic subcutaneous silicone injections in a patient with HIV-1 infection: Case report and review of the literature. *Archives of Pathology and Laboratory Medicine*, *136*(2), 204-207.

Hasegawa, K., Namba, Y., & Kimata, Y. (2013). Phalloplasty with an innervated island pedicled anterolateral thigh flap in a female-to-male transsexual. *Acta Medica Okayama*, *67*(5), 325-331.

Hassan, A., Grady, E., Ringelstein, J., Halama, J. R., Mazhari, A., & Friedman, N. C. (2012). Effect of silicone gluteal implant on bone mineral density evaluation by DXA scan. *Journal of Clinical Densitometry*, *15*(1), 124-128.

Hedjazi, A., Zarenezhad, M., Hoseinzadeh, A., Hassanzadeh, R., & Hosseini, S. M. V. (2013). Socio-demographic characteristics of transsexuals referred to the forensic medicine center in southwest of Iran. *North American Journal of Medical Sciences*, *5*(3), 224-227.

Hemiliamma, M. N., Sailarekha, N., Anandakumar, L., Murali-Mohan, K. V. (2012). Study of CD4 count in retro-viral positive eunuchs. *Indian Journal of Public Health Research and Development*, *3*(1), 125-126.

Hess, J., Rossi, N. R., Panic, L., Rubben, H., & Senf, W. (2014). Satisfaction with male-to-female gender reassignment surgery. *Deutsches Arzteblatt International*, *111*(47), 795–801.

Hewitt, J. K., Paul, C., Kasiannan, P., Grover, S. R., Newman, L. K., & Warne, G. L. (2012). Hormone treatment of gender identity disorder in a cohort of children and adolescents. *The Medical Journal of Australia*, *196*(9), 578–581.

Heylens, G., Elaut, E., Kreukels, B. P. C., Paap, M. C. S., Cerwenka S., Richter-Appelt H., … De Cuypere, G. (2014a). Psychiatric characteristics in transsexual individuals: Multicentre study in four European countries. *The British Journal of Psychiatry*, *204*(2), 151–156.

Heylens, G., Verroken, C., De Cock, S., T’Sjoen, G., & De Cuypere, G. (2014b). Effects of different steps in gender reassignment therapy on psychopathology: A prospective study of persons with a gender identity disorder. *Journal of Sexual Medicine*, *11*(1), 119–126.

Hill, S. C., Daniel, J., Benzie, A., Ayres, J., King, G., & Smith, A. (2011). Sexual health of transgender sex workers attending an inner-city genitourinary medicine clinic. *International Journal of STD & AIDS*, *22*(11), 686–687.

Hiramatsu, H., Tokashiki, R., Nakamura, H., Motohashi, R., Sakurai, E., Nomoto, M., … Suzuki, M. (2012). Analysis of high-pitched phonation using three-dimensional computed tomography. *Journal of Voice*, *26*(5), 548–554.

Hisasue, S.-I., Sasaki, S., Tsukamoto, T., & Horie, S. (2012). The relationship between second-to-fourth digit ratio and female gender identity. *Journal of Sexual Medicine*, *9*(11), 2903–2910.

Hoebeke, P. B., Decaestecker, K., Beysens, M., Opdenakker, Y., Lumen, N., & Monstrey, S. M. (2010). Erectile implants in female-to-male transsexuals: Our experience in 129 patients. *European Urology*, *57*(2), 334–340.

Hoenig, J. F. (2011). Frontal bone remodeling for gender reassignment of the male forehead: A gender-reassignment surgery. *Aesthetic Plastic Surgery*, *35*(6), 1043–1049.

Hoffman, B. (2014). An overview of depression among transgender women. *Depression Research and Treatment*, 394283. doi:[10.1155/2014/394283](http://dx.doi.org/10.1155/2014/394283)

Holmberg, E. B., Oates, J., Dacakis, G., & Grant, C. (2010). Phonetograms, aerodynamic measurements, self-evaluations, and auditory perceptual ratings of male-to-female transsexual voice. *Journal of Voice*, *24*(5), 511–522.

Hongal, S., Torwane, N. A., Goel, P., Byarakele, C., Mishra, P., & Jain, S. (2014). Oral health-related knowledge, attitude and practices among eunuchs (hijras) residing in Bhopal City, Madhya Pradesh, India: A cross-sectional questionnaire survey. *Journal of Indian Society of Periodontology*, *18*(5), 624–631.

Horvath, K. J., Iantaffi, A., Swinburne-Romine, R., & Bockting, W. (2014). A comparison of mental health, substance use, and sexual risk behaviors between rural and non-rural transgender persons. *Journal of Homosexuality*, *61*(8), 1117–1130.

Hoshiai, M., Matsumoto, Y., Sato, T., Ohnishi, M., Okabe, N., Kishimoto, Y., … Kuroda, S. (2010). Psychiatric comorbidity among patients with gender identity disorder. *Psychiatry & Clinical Neurosciences*, *64*(5), 514–519.

Hotton, A. L., Garofalo, R., Kuhns, L. M., & Johnson, A. K. (2013). Substance use as a mediator of the relationship between life stress and sexual risk among young transgender women. *AIDS Education and Prevention*, *25*(1), 62–71.

Humphries-Waa, K. (2014). The use of hormone therapy in the male-to-female transgender population: Issues for consideration in Thailand. *International Journal of Sexual Health*, *26*(1), 41–51.

Hunt, J. (2014). An initial study of transgender people’s experiences of seeking and receiving counselling or psychotherapy in the UK. *Counselling & Psychotherapy Research*, *14*(4), 288–296.

Hwahng, S. J., & Nuttbrock, L. (2014). Adolescent gender-related abuse, androphilia, and HIV risk among transfeminine people of color in New York City. *Journal of Homosexuality*, *61*(5), 691–713.

Iantaffi, A. (2011). Views from both sides of the bridge? Gender, sexual legitimacy and transgender people’s experiences of relationships. *Culture, Health & Sexuality*, *13*(3), 355–370.

Ikeda, K., Baba, T., Noguchi, H., Nagasawa, K., Endo, T., Kiya, T., & Saito, T. (2013). Excessive androgen exposure in female-to-male transsexual persons of reproductive age induces hyperplasia of the ovarian cortex and stroma but not polycystic ovary morphology. *Human Reproduction*, *28*(2), 453–461.

Inoubli, A., De Cuypere, G., Rubens, R., Heylens, G., Elaut, E., Van Caenegem, E., … T’Sjoen, G. (2011). Karyotyping, is it worthwhile in transsexualism? *Journal of Sexual Medicine*, *8*(2), 475–478.

Ishikawa, T., Kyoya, T., Nakamura, Y., Sato, E., Tomiyama, T., & Kyono, K. (2014). Oxygen consumption rate of early pre-antral follicles from vitrified human ovarian cortical tissue. *Journal of Reproduction and Development*, *60*(6), 460–467.

Jackowich, R., Johnson, T., Brassard, P., Bélanger, M., & Wassersug, R. (2014). Age of sex reassignment surgery for male-to-female transsexuals. *Archives of Sexual Behavior*, *43*(1), 13–15.

James, C., & De La Haye, W. (2011). Challenges and treatment of a transsexual in Jamaica. *Social and Economic Studies*, *60*(1), 137–152.

Janssen, A., & Erickson-Schroth, L. (2013). A new generation of gender: Learning patience from our gender nonconforming patients. *Journal of the American Academy of Child & Adolescent Psychiatry*, *52*(10), 995–997.

Jauk, D. (2013). Gender violence revisited: Lessons from violent victimization of transgender identified individuals. *Sexualities*, *16*(7), 807–825.

Jefferson, K, Neilands, T. B., & Sevelius, J. (2013). Transgender women of color: Discrimination and depression symptoms. *Ethnicity and Inequalities in Health and Social Care*, *6*(4), 121–136.

Jenness, V., & Fenstermaker, S. (2014). Agnes goes to prison: Gender authenticity, transgender inmates in prisons for men, and pursuit of “the real deal.” *Gender & Society*, *28*(1), 5–31.

Johansson, C. A., Sundbom, E., Höjerback, T., & Bodlund, O. (2010). A five-year follow-up study of Swedish adults with gender identity disorder. *Archives of Sexual Behavior*, *39*(6), 1429–1437.

Johnson, C. W, Singh, A. A., & Gonzalez, M. (2014). “It’s complicated”: Collective memories of transgender, queer, and questioning youth in high school. *Journal of Homosexuality*, *61*(3), 419–434.

Jokic-Begic, N., Korajlija, L. A., & Jurin, T. (2014). Psychosocial adjustment to sex reassignment surgery: A qualitative examination and personal experiences of six transsexual persons in Croatia. *Scientific World Journal*, 960745. doi:[10.1155/2014/960745](http://dx.doi.org/10.1155/2014/960745)

Judge, C., O’Donovan, C., Callaghan, G., Gaoatswe, G., & O’Shea, D. (2014). Gender dysphoria- Prevalence and co-morbidities in an Irish adult population. *Frontiers in Endocrinology*, *5*(87). doi:[10.3389/fendo.2014.00087](https://dx.doi.org/10.3389%2Ffendo.2014.00087)

Junger, J., Habel, U., Brohr, S., Neulen, J., Neuschaefer-Rube, C., Birkholz, P., … Pauly, K. (2014). More than just two sexes: The neural correlates of voice gender perception in gender dysphoria. *PloS One*, *9*(11), e111672. doi:[10.1371/journal.pone.0111672](https://doi.org/10.1371/journal.pone.0111672)

Kalra, G., & Shah, N. (2013). The cultural, psychiatric, and sexuality aspects of hijras in India. *International Journal of Transgenderism*, *14*(4), 171–181.

Kannan, R. Y., Sankar, T. K., & Ward, D. J. (2010). The disaster of DIY breast augmentation. *Journal of Plastic, Reconstructive & Aesthetic Surgery: JPRAS*, *63*(1), e100–e101.

Kannangara, D. R., Roberts, D. M., Furlong, T. J., Graham, G. G., Williams, K. M., & Day, R. O. (2012). Oxypurinol, allopurinol and allopurinol-1-riboside in plasma following an acute overdose of allopurinol in a patient with advanced chronic kidney disease. *British Journal of Clinical Pharmacology*, *73*(5), 828–829.

Karpel, L., & Cordier, B. (2013). Postoperative regrets after sex reassignment surgery: A case report. *Sexologies*, *22*(2), 81–89.

Kaufmann, J. (2010a). Narrative and the re/production of transsexual: the foreclosure of an endured emergence of gender multiplicity. *Cultural Studies - Critical Methodologies*, *10*(2), 91–99.

Kaufmann, J. (2010b). Trans-representation. *Qualitative Inquiry*, *16*(2), 104–115.

Kaufmann, J. (2014). Masculinist method: A cautionary tale. *Qualitative Research Journal*, *14*(1), 41–49.

Kaufmann, U., Domig, K. J., Lippitsch, C. I., Kraler, M., Marschalek, J., Kneifel, W., … Petricevic, L. (2014). Ability of an orally administered lactobacilli preparation to improve the quality of the neovaginal microflora in male to female transsexual women. *European Journal of Obstetrics & Gynecology & Reproductive Biology*, *172*, 102–105.

Kauth, M. R., Shipherd, J. C., Lindsay, J., Blosnich, J. R., Brown, G. R., & Jones, K. T. (2014). Access to care for transgender veterans in the veterans health administration: 2006-2013. *American Journal of Public Health*, *104*(S4), S532–S534.

Kedia, G. T., Oelke, M., Sohn, M., Kuczyk, M. A., & Ãœckert, S. (2013). Pharmacologic Characterization of Human Male Urethral Smooth Muscle: An In Vitro Approach. *Urology*, *82*(6), 1451.e13–1451.e19.

Kern, L., Edmonds, P., Perrin, E. C., & Stein, M. T. (2014). An 8-year-old biological female who identifies herself as a boy: Perspectives in primary care and from a parent. *Journal of Developmental and Behavioral Pediatrics*, *35*(4), 301–303.

Khan, O., & Sim, J. J. (2010). Silicone-induced granulomas and renal failure. *Dialysis and Transplantation*, *39*(6), 254–259.

Khatchadourian, K., Amed, S., & Metzger, D. L. (2014). Clinical management of youth with gender dysphoria in Vancouver. *The Journal of Pediatrics*, *164*(4), 906–911.

Khazal, S., Abdel-Azim, H., Kapoor, N., & Mahadeo, K. M. (2014). Overcoming psychosocial and developmental barriers to blood and marrow transplantation (BMT) in an adolescent/young adult (AYA) transgender patient with chronic myelogenous leukemia. *Pediatric Hematology and Oncology*, *31*(8), 765–767.

Khoosal, D., Grover P., & Terry, T. (2011). Satisfaction with a gender realignment service. *Sexual and Relationship Therapy*, *26*(1), 72–83.

Kim, S. K., Kim, T. H., Yang, J. I., Kim, M. H., Kim, M. S., & Lee, K. C. (2012). The etiology and treatment of the softened phallus after the radial forearm osteocutaneous free flap phalloplasty. *Archives of Plastic Surgery*, *39*(4), 390–396.

Kim, S. K., Moon, J. B., Heo, J., Kwon, Y. S., & Lee, K. C. (2010). A new method of urethroplasty for prevention of fistula in female-to-male gender reassignment surgery. *Annals of Plastic Surgery*, *64*(6), 759–764.

King, A. (2012). The dawn of a new identity: Aspects of a relational approach to psychotherapy with a transsexual client. *British Journal of Psychotherapy*, *28*(1), 35–49.

Király, I., Pataricza, J., Bajory, Z., Simonsen, U., Varro, A., Papp, J. G., … Kun, A. (2013). Involvement of large-conductance Ca2+-activated K+ channels in both nitric oxide and endothelium-derived hyperpolarization-type relaxation in human penile small arteries. *Basic & Clinical Pharmacology & Toxicology*, *113*(1), 19–24.

Kise, K., & Nguyen, M. (2011). Adult baby syndrome and gender identity disorder. *Archives of Sexual Behavior*, *40*(5), 857–859.

Knight, E. J, & McDonald, M. J. (2013). Recurrence and progression of meningioma in male-to-female transgender individuals during exogenous hormone use. *International Journal of Transgenderism*, *14*(1), 18–23.

Kosenko, K. A. (2010). Meanings and dilemmas of sexual safety and communication for transgender individuals. *Health Communication*, *25*(2), 131–141.

Kosenko, K. A. (2011a). Contextual influences on sexual risk-taking in the transgender community. *Journal of Sex Research*, *48*(2-3), 285–296.

Kosenko, K. A. (2011b). The safer sex communication of transgender adults: Processes and problems. *Journal of Communication*, *61*(3), 476–495.

Kosenko, K., Rintamaki, L., Raney, S., & Maness, K. (2013). Transgender patient perceptions of stigma in health care contexts. *Medical Care*, *51*(9), 819–822.

Kozee, H. B., Tylka, T. L., & Bauerband, L. A. (2012). Measuring transgender individuals’ comfort with gender identity and appearance: Development and validation of the transgender congruence scale. *Psychology of Women Quarterly*, *36*(2), 179–196.

Kranz, G. S., Hahn, A., Baldinger, P., Haeusler, D., Philippe, C., Kaufmann, U., … Lanzenberger, R. (2014a). Cerebral serotonin transporter asymmetry in females, males and male-to-female transsexuals measured by PET in vivo. *Brain Structure & Function*, *219*(1), 171–183.

Kranz, G. S., Hahn, A., Kaufmann, U., Küblböck, M., Hummer, A., Ganger, S., … Lanzenberger, R. (2014b). White matter microstructure in transsexuals and controls investigated by diffusion tensor imaging. *Journal of Neuroscience*, *34*(46), 15466–15475.

Krell, E. (2013). Contours through covers: Voice and affect in the music of Lucas Silveira. *Journal of Popular Music Studies*, *25*(4), 476–503.

Kreukels, B. P. C., & Cohen-Kettenis, P. T. (2011). Puberty suppression in gender identity disorder: The Amsterdam experience. *Nature Reviews Endocrinology*, *7*(8), 466–472.

Kreukels, B. P. C., Haraldsen, I. R., De Cuypere, G., Richter-Appelt, H., Gijs, L., & Cohen-Kettenis, P. T. (2012). A European network for the investigation of gender incongruence: The ENIGI initiative. *European Psychiatry*, *27*(6), 445–450.

Krishnan, A. S., & Barrett, T. (2012). Westermark sign in pulmonary embolism. *New England Journal of Medicine*, *366*(11), e16. doi:10.1056/NEJMicm1107936

Krum, T. E., Davis, K. S., & Galupo, M. P. (2013). Gender-inclusive housing preferences: A survey of college-aged transgender students. *Journal of LGBT Youth*, *10*(1-2), 64–82.

Ku, H. L., Lin, C. S., Chao, H. T., Tu, P. C., Li, C. T., Cheng, C. M., ... & Hsieh, J. C. (2013). Brain signature characterizing the body-brain-mind axis of transsexuals. *PloS One*, *8*(7), e70808. doi:[10.1371/journal.pone.0070808](https://doi.org/10.1371/journal.pone.0070808)

Kuhn, A., Santi, A., & Birkhäuser, M. (2011). Vaginal prolapse, pelvic floor function, and related symptoms 16 years after sex reassignment surgery in transsexuals. *Fertility & Sterility*, *95*(7), 2379–2382.

Kumar, K., & Gupta, M. (2012). Social dichotomy versus gender dichotomy: A case report of gender identity disorder. *Indian Journal of Psychological Medicine*, *34*(2), 190–192.

Künzel, H. E., Murck, H., Stalla, G. K., & Steiger, A. (2011). Changes in the sleep electroencephalogram (EEG) during male to female transgender therapy. *Psychoneuroendocrinology*, *36*(7), 1005–1009.

Kuper, L. E., Nussbaum, R., & Mustanski, B. (2012). Exploring the diversity of gender and sexual orientation identities in an online sample of transgender individuals. *Journal of Sex Research*, *49*(2-3), 244–254.

Kuper L. E., Wright, L., & Mustanski, B. (2014). Stud identity among female-born youth of color: Joint conceptualizations of gender variance and same-sex sexuality. *Journal of Homosexuality*, *61*(5), 714–731.

Kurahashi, H., Watanabe, M., Sugimoto, M., Ariyoshi, Y., Mahmood, S., Araki, M., … Kumon, H. (2013). Testosterone replacement elevates the serum uric acid levels in patients with female to male gender identity disorder. *Endocrine Journal*, *60*(12), 1321–1327.

Kyoya, T., Nakamura, Y., Miyatani, S., Miyagawa, T., Tomiyama, T., & Kyono, K. (2014). Evaluation of oxygen consumption in human vitrified and warmed pre-antral follicles after prolonged low temperatures. *Reproductive Medicine & Biology*, *13*(1), 47–52.

Laidlaw, E., & Irwig, M. (2013). Risks and benefits of estrogen therapy for a male-to-female transsexual with a prothrombin gene mutation. *Endocrine Practice*, *19*(6), e150–e153.

Lauerma, H., Voutilainen, J., & Tuominen, T. (2010). Matricide and two sexual femicides by a male strangler with a transgender sadomasochistic identity. *Journal of Forensic Sciences*, *55*(2), 549–550.

Law, C. L., Martinez, L. R., Ruggs, E. N., Hebl, M. R., & Akers, E. (2011). Trans-parency in the workplace: How the experiences of transsexual employees can be improved. *Journal of Vocational Behavior*, *79*(3), 710–723.

Lawrence, A. A. (2010). Societal individualism predicts prevalence of nonhomosexual orientation in male-to-female transsexualism. *Archives of Sexual Behavior*, *39*(2), 573–583.

Lee, K. C., Huang, C. Y., & Wang, P. H. (2012). Parasitic peritoneal leiomyomatosis mimicking intra-abdominal abscess with hematoma. *Taiwanese Journal of Obstetrics and Gynecology*, *51*(1), 115–116.

Leibowitz, S. F., & Spack, N. P. (2011). The development of a gender identity psychosocial clinic: Treatment issues, logistical considerations, interdisciplinary cooperation, and future initiatives. *Child and Adolescent Psychiatric Clinics of North America*, *20*(4), 701–724.

Leinung, M., Urizar, M., Patel, N., & Sood, S. (2013). Endocrine treatment of transsexual persons: Extensive personal experience. *Endocrine Practice*, *19*(4), 644–650.

Lemaire, M., Thomazeau, B., & Bonnet-Brilhault, F. (2014). Gender identity disorder and autism spectrum disorder in a 23-year-old female. *Archives of Sexual Behavior*, *43*(2), 395–398.

Lemma, A. (2012). Research off the couch: Re-visiting the transsexual conundrum. *Psychoanalytic Psychotherapy*, *26*(4), 263–281.

Lemma, A. (2013). The body one has and the body one is: Understanding the transsexual’s need to be seen. *International Journal of Psychoanalysis*, *94*(2), 277–292.

Levitt, H. M., & Ippolito, M. R. (2014a). Being transgender: Navigating minority stressors and developing authentic self-presentation. *Psychology of Women Quarterly*, *38*(1), 46–64.

Levitt, H. M., & Ippolito, M. R. (2014b). Being transgender: The experience of transgender identity development. *Journal of Homosexuality*, *61*(12), 1727–1758.

Levy, D. L., & Lo, J. R. (2013). Transgender, transsexual, and gender queer individuals with a Christian upbringing: The process of resolving conflict between gender identity and faith. *Journal of Religion and Spirituality in Social Work*, *32*(1), 60–83.

Lewis, S. T., & Johnson, C. W. (2011). “But it’s not that easy”: Negotiating (trans)gender expressions in leisure spaces. *Leisure/ Loisir*, *35*(2), 115–132.

Leyngold, M. M., & Rivera-Serrano, C. M. (2014). Microvascular penile replantation utilizing the deep inferior epigastric vessels. *Journal of Reconstructive Microsurgery*, *30*(8), 581–584.

Liedtke, M. D., Vanguri, A., & Rathbun, R. C. (2012). A probable interaction between warfarin and the antiretroviral TRIO study regimen. *Annals of Pharmacotherapy*, *46*(11), e34. doi:10.1345/aph.1R290

Light, A. D., Obedin-Maliver, J., Sevelius, J. M., & Kerns, J. L. (2014). Transgender men who experienced pregnancy after female-to-male gender transitioning. *Obstetrics and Gynecology*, *124*(6), 1120–1127.

Lin, C. S., Ku, H. L., Chao, H. T., Tu, P. C., Li, C. T., Cheng, C. M., … Hsieh, J. C. (2014). Neural network of body representation differs between transsexuals and cissexuals. *PloS One*, *9*(1), e85914. doi:[10.1371/journal.pone.0085914](https://doi.org/10.1371/journal.pone.0085914)

Lin, Y. C., Lin, W. C., & Hsu, J. M. (2013). Urethral stricture in male-to-female transsexual patients- Report of two cases. *Formosan Journal of Surgery*, *46*(5), 173–175.

Lombardo, F., Toselli, L., Grassetti, D., Paoli, D., Masciandaro, P., Valentini, F., … Gandini, L. (2013). Hormone and genetic study in male to female transsexual patients. *Journal of Endocrinological Investigation*, *36*(8), 550–557.

Longfield, K., Panyanouvong, X., Chen, J., & Kays, M. B. (2011). Increasing safer sexual behavior among Lao kathoy through an integrated social marketing approach. *BMC Public Health*, *11*(872). doi:10.1186/1471-2458-11-872

Lopez, G. J., Hoffman, R. S., & Davenport, M. (2011). Plantaris rupture: A mimic of deep venous thrombosis. *Journal of Emergency Medicine*, *40*(2), e27–e30.

Luders, E., Sanchez, F. J., Tosun, D., Shattuck, D. W., Gaser, C., Vilain, E., & Toga, A. W. (2012). Increased cortical thickness in male-to-female transsexualism. *Journal of Behavioral and Brain Science*, *2*(3), 357–362.

Luecke, J. C. (2011). Working with transgender children and their classmates in pre-adolescence: Just be supportive. *Journal of LGBT Youth*, *8*(2), 116–156.

Macdonald, J. (2013). An autoethnography of queer transmasculine femme incoherence and the ethics of trans research. *Studies in Symbolic Interaction*, *40*, 129–152.

Macdonnell, J. A., & Grigorovich, A. (2012). Gender, work, and health for trans health providers: A focus on transmen. *ISRN Nursing*, 161097. doi:10.5402/2012/161097

Maglione, K. D., Margolies, L., Jaffer, S., Szabo, J., Schmidt, H., Weltz, C., & Sonnenblick, E. B. (2014). Breast cancer in male-to-female transsexuals: Use of breast imaging for detection. *American Journal of Roentgenology*, *203*(6), W735–W740.

Maguen, S., & Shipherd, J. C. (2010). Suicide risk among transgender individuals. *Psychology and Sexuality*, *1*(1), 34–43.

Mahalingam, G., Ricanek, K. Jr, & Albert, A. M. (2014). Investigating the periocular-based face recognition across gender transformation. *IEEE Transactions on Information Forensics and Security*, *9*(12), 2180–2192.

Males, S., Joly, V., Adle-Biassette, H., Abgrall, S., Lariven, S., Leboulanger, N., & Yeni, P. (2010). Silicone in HIV-1-infected patients: A cause of misdiagnosed granulomatous disease. *International Journal of Infectious Diseases*, *14*(S3), e277–e279.

Mandlis, L. R. (2011). A passport to trouble: Bureaucratic incompetence as censorship. *Journal of Information Ethics*, *20*(2), 85–102.

Manieri, C., Castellano, E., Crespi, C., Di Bisceglie, C., Dell’Aquila, C., Gualerzi, A., & Molo, M. (2014). Medical treatment of subjects with gender identity disorder: The experience in an Italian public health center. *International Journal of Transgenderism*, *15*(2), 53–65.

Mann, S. L. (2011). Drag queens’ use of language and the performance of blurred gendered and racial identities. *Journal of Homosexuality*, *58*(6-7), 793–811.

Marciano, A. (2014). Living the VirtuReal: Negotiating transgender identity in cyberspace. *Journal of Computer-Mediated Communication*, *19*(4), 824–838.

Marcus, B. F., & McNamara, S. (2013). “Strange and otherwise unaccountable actions”: Category, conundrum, and trans identities. *Journal of the American Psychoanalytic Association*, *61*(1), 45–66.

Maree, J. G. (2014). Career construction with a gay client: A case study. *British Journal of Guidance & Counselling*, *42*(4), 436–449.

Martin, K. A., Bostwick, J. M., & Vargas, H. E. (2011). Liver transplant case report: Transgenderism and liver transplantation. *International Journal of Transgenderism*, *13*(1), 45–49.

Martins, T. A., Kerr, L. R. F. S., Macena, R. H. M., Mota, R. S., Carneiro, K. L., Gondim, R. C., & Kendall, C. (2013). Travestis, an unexplored population at risk of HIV in a large metropolis of northeast Brazil: A respondent-driven sampling survey. *AIDS Care*, *25*(5), 606–612.

Mastronikolis, N. S., Remacle, M., Biagini, M., Kiagiadaki, D., & Lawson, G. (2013). Wendler glottoplasty: An effective pitch raising surgery in male-to-female transsexuals. *Journal of Voice*, *27*(4), 516–522.

Masumori, N., & Tsukamoto, T. (2014). Risk factors for the development of well leg compartment syndrome after sex reassignment surgery in patients with gender identity disorder. *International Journal of Urology*, *21*(6), 623–624.

Mathew, H., & Hamid, B. (2013). Context is everything: An unusual breast core biopsy case. *International Journal of Surgical Pathology*, *21*(5), 502–503.

Maycock, L. B., & Kennedy, H. P. (2014). Breast care in the transgender individual. *Journal of Midwifery & Women’s Health*, *59*(1), 74–81.

Mazumder, A., Kabir, S. M. I., Patowary, A., & Chaliha, R. (2013). A case of sexual perversion: A case report. *Journal of Punjab Academy of Forensic Medicine and Toxicology*, *13*(1), 28–29.

McDuffie, E., & Brown, G. R. (2010). 70 U.S. veterans with gender identity disturbances: A descriptive study. *International Journal of Transgenderism*, *12*(1), 21–30.

McMullin, D. T. (2011). Fa’afafine notes: On Tagaloa, Jesus, and Nafanua. *Amerasia Journal*, *37*(3), 115–131.

Meier, S. L. C,, Fitzgerald, K. M., Pardo, S. T., & Babcock, J. (2011). The effects of hormonal gender affirmation treatment on mental health in female-to-male transsexuals. *Journal of Gay and Lesbian Mental Health*, *15*(3), 281–299.

Meier, S., Pardo, S., Labuski, C., & Babcock, J. (2013a). Measures of clinical health among female-to-male transgender persons as a function of sexual orientation. *Archives of Sexual Behavior*, *42*(3), 463–474.

Meier, S. L., Sharp, C., Michonski, J., Babcock, J. C., & Fitzgerald, K. (2013b). Romantic relationships of female-to-male trans men: A descriptive study. *International Journal of Transgenderism*, *14*(2), 75–85.

Mendonca, D., Leitao, D. S., Friend, R., Epelboim, J., & Eiger, G. (2012). An unusual case of pulmonary embolism. *Respiratory Care*, *57*(8), 1345–1347.

Mepham, N., Bouman, W. P., Arcelus, J., Hayter, M., & Wylie, K. R. (2014). People with gender dysphoria who self-prescribe cross-sex hormones: Prevalence, sources, and side effects knowledge. *Journal of Sexual Medicine*, *11*(12), 2995–3001.

Merryfeather, L., & Bruce, A. (2014). The invisibility of gender diversity: Understanding transgender and transsexuality in nursing literature. *Nursing Forum*, *49*(2), 110–123.

Meybodi, A. M., Hajebi, A., & Jolfaei, A. G. (2014a). Psychiatric axis I comorbidities among patients with gender dysphoria. *Psychiatry Journal*, 971814. doi:[10.1155/2014/971814](http://dx.doi.org/10.1155/2014/971814)

Meybodi, A. M., Hajebi, A., & Jolfaei, A. G. (2014b). The frequency of personality disorders in patients with gender identity disorder. *Medical Journal of the Islamic Republic of Iran*, *28*(1), 582-587.

Mihm, L. B., Swetman, G., Boh, E. E., Wang, A., & Witzig, R. (2010). Patient with AIDS and acute circinate skin eruptions. *Clinical Infectious Diseases*, *51*(8), 929–930.

Miller, J., & Nichols, A. (2012). Identity, sexuality and commercial sex among Sri Lankan nachchi. *Sexualities*, *15*(5/6), 554–569.

Miner, M. H., Bockting, W. O., Romine, R. S., & Raman, S. (2012). Conducting internet research with the transgender population: Reaching broad samples and collecting valid data. *Social Science Computer Review*, *30*(2), 202–211.

Mishra, R. (2012). The case: IVF treatment for an HIV-discordant transgender couple? *Cambridge Quarterly of Healthcare Ethics  21*(2), 281.

Miyajima, E., Taira, N., Koda, M., & Kondo, T. (2014). Differences in personality traits between male-to-female and female-to-male gender identity disorder subjects. *Psychiatry Research*, *220*(1/2), 496–499.

Miyajima, T., Kim, Y. T., & Oda, H. (2012). A study of changes in bone metabolism in cases of gender identity disorder. *Journal of Bone and Mineral Metabolism*, *30*(4), 468–473.

Mizock, L., & Fleming, M. Z. (2011). Transgender and gender variant populations with mental illness: Implications for clinical care. *Professional Psychology: Research and Practice*, *42*(2), 208–213.

Mizock, L., & Mueser, K. T. (2014). Employment, mental health, internalized stigma, and coping with transphobia among transgender individuals. *Psychology of Sexual Orientation and Gender Diversity*, *1*(2), 146–158.

Mokonogho, J., Mittal, S., & Quitangon, G. (2010). Treating the transgender homeless population: Experiences during residency training. *Journal of Gay & Lesbian Mental Health*, *14*(4), 346–354.

Monsour, M., & Rawlins, W. K. (2014). Transitional identities and postmodern cross-gender friendships: An exploratory investigation. *Women and Language*, *37*(1), 11-39.

Monstrey, S. J, Ceulemans, P., & Hoebeke, P. (2011). Sex reassignment surgery in the female-to-male transsexual. *Seminars in Plastic Surgery*, *25*(3), 229–244.

Moody, C., & Smith, N. G. (2013). Suicide protective factors among trans adults. *Archives of Sexual Behavior*, *42*(5), 739–752.

Moreman, S. T., & McIntosh, D. M. (2010). Brown scriptings and rescriptings: A critical performance ethnography of Latina drag queens. *Communication and Critical/ Cultural Studies*, *7*(2), 115–135.

Morgan, S. W., & Stevens, P. E. (2012). Transgender identity development as represented by a group of transgendered adults. *Issues in Mental Health Nursing*, *33*(5), 301–308.

Motmans, J., Meier, P., Ponnet, K., & T’Sjoen, G. (2012). Female and male transgender quality of life: Socioeconomic and medical differences. *Journal of Sexual Medicine*, *9*(3), 743–750.

Motmans, J., Ponnet, K., & De Cuypere, G. (2014). Sociodemographic characteristics of trans persons in Belgium: A secondary data analysis of medical, state, and social data. *Archives of Sexual Behavior,* *44*(5), 1289-1299.

Muccino, E., Gentile, G., Marchesi, M., & Zoja, R. (2014). The homicide of a transgender by an ante-mortem “incaprettamento”. A case report. *Romanian Journal of Legal Medicine*, *22*(3), 157–160.

Mueller, A., Haeberle, L., Zollver, H., Claassen, T., Kronawitter, D., Oppelt, P. G., … Dittrich, R. (2010). Effects of intramuscular testosterone undecanoate on body composition and bone mineral density in female-to-male transsexuals. *Journal of Sexual Medicine*, *7*(9), 3190–3198.

Mueller, A., Zollver, H., Kronawitter, D., Oppelt, P. G., Claassen, T., Hoffmann, I., … Dittrich, R. (2011). Body composition and bone mineral density in male-to-female transsexuals during cross-sex hormone therapy using gonadotrophin-releasing hormone agonist. *Experimental and Clinical Endocrinology and Diabetes*, *119*(2), 95–100.

Muhr, S. L., & Sullivan, K. R. (2013). “None so queer as folk”: Gendered expectations and transgressive bodies in leadership. *Leadership*, *9*(3), 416–435.

Mullen, G., & Moane, G. (2013). A qualitative exploration of transgender identity affirmation at the personal, interpersonal, and sociocultural levels. *International Journal of Transgenderism*, *14*(3), 140–154.

Murad, M. H., Elamin, M. B., Garcia, M. Z., Mullan, R. J., Murad, A., Erwin, P. J., & Montori, V. M. (2010). Hormonal therapy and sex reassignment: A systematic review and meta-analysis of quality of life and psychosocial outcomes. *Clinical Endocrinology*, *72*(2), 214–231.

Murray, S. B., Boon, E., & Touyz, S. W. (2013). Diverging eating psychopathology in transgendered eating disorder patients: A report of two cases. *Eating Disorders*, *21*(1), 70–74.

Murty, O. P. (2010). Male-to-female transsexual on estrogen: Sudden death due to pulmonary thromboembolism. *Journal of Forensic Medicine and Toxicology*, *27*(1), 27–34.

Nadal, K. L., Davidoff, K. C., Davis, L. S., & Wong, Y. (2014). Emotional, behavioral, and cognitive reactions to microaggressions: Transgender perspectives. *Psychology of Sexual Orientation and Gender Diversity*, *1*(1), 72–81.

Nadal, K. L., Skolnik, A., & Wong, Y. (2012). Interpersonal and systemic microaggressions toward transgender people: Implications for counseling. *Journal of LGBT Issues in Counseling*, *6*(1), 55–82.

Nagoshi, J. L., Brzuzy, S., & Terrell, H. K. (2012). Deconstructing the complex perceptions of gender roles, gender identity, and sexual orientation among transgender individuals. *Feminism & Psychology*, *22*(4), 405–422.

Nakamura, A., Watanabe, M., Sugimoto, M., Sako, T., Mahmood, S., Kaku, H., … Kumon, H. (2013). Dose-response analysis of testosterone replacement therapy in patients with female to male gender identity disorder. *Endocrine Journal*, *60*(3), 275–281.

Nash, C. J. (2011). Trans experiences in lesbian and queer space. *Canadian Geographer*, *55*(2), 192–207.

Nawata, H., Ogomori, K., Tanaka, M., Nishimura, R., Urashima, H., Yano, R., … Kuwabara, Y. (2010). Regional cerebral blood flow changes in female to male gender identity disorder. *Psychiatry & Clinical Neurosciences*, *64*(2), 157–161.

Nemoto, T., Bödeker, B., & Iwamoto, M. (2011). Social support, exposure to violence and transphobia, and correlates of depression among male-to-female transgender women with a history of sex work. *American Journal of Public Health*, *101*(10), 1980–1988.

Nemoto, T., Bodeker, B., Iwamoto, M., & Sakata, M. (2014). Practices of receptive and insertive anal sex among transgender women in relation to partner types, sociocultural factors, and background variables. *AIDS Care*, *26*(4), 434–440.

Nemoto, T., Iwamoto, M., Perngparn, U., Areesantichai, C., Kamitani, E., & Sakata, M. (2012). HIV-related risk behaviors among kathoey (male-to-female transgender) sex workers in Bangkok, Thailand. *AIDS Care*, *24*(2), 210–219.

Neto, R. R., Hintz, F., Krege, S., Rübben, H., & vom Dorp, F. (2012). Gender reassignment surgery - A 13 year review of surgical outcomes. *International Braz J Urol*, *38*(1), 97–107.

Nichols, A. (2010). Dance ponnaya, dance! Police abuses against transgender sex workers in Sri Lanka. *Feminist Criminology*, *5*(2), 195–222.

Nichols, J. (2013). Rie’s story, Ryan’s journey: Music in the life of a transgender student. *Journal of Research in Music Education*, *61*(3), 262–279.

Nieder, T. O., Herff, M., Cerwenka, S., Preuss, W. F., Cohen-Kettenis, P. T., De Cuypere, G. … Richter-Appelt, H. (2011). Age of onset and sexual orientation in transsexual males and females. *Journal of Sexual Medicine*, *8*(3), 783–791.

Nikolic, D. V., Djordjevic, M. L., Granic, M., Nikolic, A. T., Stanimirovic, V. V., Zdravkovic D., & Jelic, S. (2012). Importance of revealing a rare case of breast cancer in a female to male transsexual after bilateral mastectomy. *World Journal of Surgical Oncology*, *10*(280). doi:10.1186/1477-7819-10-280

Nistal, M, Gonzalez-Peramato, P., & De Miguel, M. P. (2013). Sertoli cell dedifferentiation in human cryptorchidism and gender reassignment shows similarities between fetal environmental and adult medical treatment estrogen and antiandrogen exposure. *Reproductive Toxicology*, *42*, 172–179.

Nordmarken, S. (2014). Becoming ever more monstrous: Feeling transgender in-betweenness. *Qualitative Inquiry*, *20*(1), 37–50.

Nuru, A. K. (2014). Between layers: Understanding the communicative negotiation of conflicting identities by transgender individuals. *Communication Studies*, *65*(3), 281–297.

Nuttbrock, L., Bockting, W., Mason, M., Hwahng, S., Rosenblum, A., Macri, M., & Becker, J. (2011). A further assessment of Blanchard’s typology of homosexual versus non-homosexual or autogynephilic gender dysphoria. *Archives of Sexual Behavior*, *40*(2), 247–257.

Nuttbrock, L., Bockting, W., Rosenblum, A., Hwahng, S., Mason, M., Macri, M., & Becker, J. (2013). Gender abuse, depressive symptoms, and HIV and other sexually transmitted infections among male-to-female transgender persons: A three-year prospective study. *American Journal of Public Health*, *103*(2), 300–307.

Nuttbrock, L., Bockting, W., Rosenblum, A., Hwahng, S., Mason, M., Macri, M., & Becker, J. (2014a). Gender abuse and major depression among transgender women: A prospective study of vulnerability and resilience. *American Journal of Public Health*, *104*(11), 2191–2198.

Nuttbrock, L., Bockting, W., Rosenblum, A., Hwahng, S., Mason, M., Macri, M., & Becker, J. (2014b). Gender abuse, depressive symptoms, and substance use among transgender women: A 3-year prospective study. *American Journal of Public Health*, *104*(11), 2199–2206.

Nuttbrock, L., Bockting, W., Rosenblum, A., Mason, M., Macri, M., & Becker, J. (2012). Gender identity conflict/affirmation and major depression across the life course of transgender women. *International Journal of Transgenderism*, *13*(3), 91–103.

Nuttbrock, L., Hwahng, S., Bockting, W., Rosenblum, A., Mason, M., Macri, M., & Becker, J. (2010). Psychiatric impact of gender-related abuse across the life course of male-to-female transgender persons. *Journal of Sex Research*, *47*(1), 12–23.

Ocha, W. (2012). Transsexual emergence: Gender variant identities in Thailand. *Culture, Health & Sexuality*, *14*(5-6), 563–575.

Offman, H. (2014). The princess and the penis: A post postmodern queer-y tale. *Psychoanalytic Dialogues*, *24*(1), 72–87.

Oh, S. K., Kim, G. W., Yang, J. C., Kim, S. K., Kang, H. K., & Jeong, G. W. (2012). Brain activation in response to visually evoked sexual arousal in male-to-female transsexuals: 3.0 tesla functional magnetic resonance imaging. *Korean Journal of Radiology*, *13*(3), 257–264.

Operario, D., Nemoto, T., Iwamoto, M., & Moore, T. (2011). Unprotected sexual behavior and HIV risk in the context of primary partnerships for transgender women. *AIDS and Behavior*, *15*(3), 674–682.

Operario, D., Yang, M.-F., Reisner, S. L., Iwamoto, M., & Nemoto, T. (2014). Stigma and the syndemic of HIV-related health risk behaviors in a diverse sample of transgender women. *Journal of Community Psychology*, *42*(5), 544–557.

Oster, J. M., Shastri, P, & Geyer, C. (2010). Cerebral venous sinus thrombosis after gender reassignment surgery. *Gender Medicine*, *7*(3), 270–275.

Ott, J., Aust, S., Promberger, R., Huber, J. C., & Kaufmann, U. (2011). Cross-sex hormone therapy alters the serum lipid profile: A retrospective cohort study in 169 transsexuals. *Journal of Sexual Medicine*, *8*(8), 2361–2369.

Ott, J., Kaufmann, U., Bentz, E. K., Huber, J. C., & Tempfer, C. B. (2010a). Incidence of thrombophilia and venous thrombosis in transsexuals under cross-sex hormone therapy. *Fertility and Sterility*, *93*(4), 1267–1272.

Ott, J., van Trotsenburg, M., Kaufmann, U., Schrögendorfer, K., Haslik, W., Huber, J. C., & Wenzl, R. (2010b). Combined hysterectomy/salpingo-oophorectomy and mastectomy is a safe and valuable procedure for female-to-male transsexuals. *Journal of Sexual Medicine*, *7*(6), 2130–2138.

Paap, M. C. S., & Haraldsen, I. R. (2010). Sex-based differences in answering strategy and the influence of cross-sex hormones. *Psychiatry Research*, *175*(3), 266–270.

Paap, M., Kreukels, B. P., Cohen‐Kettenis, P. T., Richter‐Appelt, H., de Cuypere, G., & Haraldsen, I. R. (2011). Assessing the utility of diagnostic criteria: A multisite study on gender identity disorder. *Journal of Sexual Medicine*, *8*(1), 180–190.

Pacchiarotti, J., Ramos, T., Howerton, K., Greilach, S., Zaragoza, K., Olmstead, M., & Izadyar, F. (2013). Developing a clinical-grade cryopreservation protocol for human testicular tissue and cells. *BioMed Research International*, 930962. doi:[10.1155/2013/930962](http://dx.doi.org/10.1155/2013/930962)

Page, A. D., & Peacock, J. R. (2013). Negotiating identities in a heteronormative context. *Journal of Homosexuality*, *60*(4), 639–654.

Palmer, D., Dietsch, A., & Searl, J. (2012). Endoscopic and stroboscopic presentation of the larynx in male-to-female transsexual persons. *Journal of Voice*, *26*(1), 117–126.

Park, S., Cheng, C. P., Lim, L. T., & Gerber, D. (2014). Secondary intracranial hypertension from testosterone therapy in a transgender patient. *Seminars in Ophthalmology*, *29*(3), 156–158.

Parkinson, J. (2014). Gender dysphoria in Asperger’s syndrome: A caution. *Australasian Psychiatry*, *22*(1), 84–85.

Parola, N., Bonierbale, M., Lemaire, A., Aghababian, V., Michel, A., & Lançon, C. (2010). Study of quality of life for transsexuals after hormonal and surgical reassignment. *Sexologies*, *19*(1), 24–28.

Pasterski, V., Gilligan, L., & Curtis, R. (2014). Traits of autism spectrum disorders in adults with gender dysphoria. *Archives of Sexual Behavior*, *43*(2), 387–393.

Pattison, S. T., & Mclaren, B. R. (2013). Triple negative breast cancer in a male-to-female transsexual. *Internal Medicine Journal*, *43*(2), 203–205.

Patton, J., & Reicherzer, S. (2010). Inviting “Kate’s” authenticity: Relational cultural theory applied in work with a transsexual sex worker of color using the competencies for counseling with transgender clients. *Journal of LGBT Issues in Counseling*, *4*(3-4), 214–227.

Pauley, D. (2014). Gender, panic and relational scaffolding in the therapy of a latency-aged boy. *Journal of Gay & Lesbian Mental Health*, *18*(2), 230–236.

Pawa, D., Firestone, R., Ratchasi, S., Dowling, O., Jittakoat, Y., Duke, A., & Mundy, G. (2013). Reducing HIV risk among transgender women in Thailand: A quasi-experimental evaluation of the sisters program. *PloS One*, *8*(10), e77113. doi:[10.1371/journal.pone.0077113](https://doi.org/10.1371/journal.pone.0077113)

Pelusi, C., Costantino, A., Martelli, V., Lambertini, M., Bazzocchi, A., Ponti, F., … Meriggiola, M. C. (2014). Effects of three different testosterone formulations in female-to-male transsexual person. *Journal of Sexual Medicine*, *11*(12), 3002–3011.

Perrin, E., Smith, N., Davis, C., Spack, N., & Stein, M. T. (2010). Gender variant and gender dysphoria in two young children. *Journal of Developmental and Behavioral Pediatrics*, *31*(2), 161–164.

Perrone, A. M., Scifo, M. C., Martelli, V., Casadio, P., Morselli, P. G., Pelusi, G., & Meriggiola, M. C. (2010). Hysterectomy and bilateral salpingoovariectomy in a transsexual subject without visible scarring. *Diagnostic and Therapeutic Endoscopy*, 845029. doi:[10.1155/2010/845029](http://dx.doi.org/10.1155/2010/845029)

Perry, B., & Dyck, D. R. (2014). “I don’t know where it is safe”: Trans women’s experiences of violence. *Critical Criminology*, *22*(1), 49–63.

Perucchi, J., Brandão, B. C., Berto, C. M. G., Rodrigues, F. D., & da Silva, J. A. (2014). Brazil’s Unified Health System (SUS) and its treatment for transgender people. *Psychology*, *5*(9), 1090–1094.

Petricevic, L., Kaufmann, U., Domig, K. J., Kraler, M., Marschalek, J., Kneifel, W., & Kiss, H. (2014a). Molecular detection of Lactobacillus species in the neovagina of male-to-female transsexual women. *Scientific Reports*, *4*, 3746. doi:10.1038/srep03746

Petricevic, L., Kaufmann, U., Domig, K. J., Kraler, M., Marschalek, J., Kneifel, W., & Kiss, H. (2014b). Rectal lactobacillus species and their influence on the vaginal microflora: A model of male-to-female transsexual women. *Journal of Sexual Medicine*, *11*(11), 2738–2743.

Pieper, L. P. (2012). Gender regulation: Renée Richards revisited. *International Journal of the History of Sport*, *29*(5), 675–690.

Pimenoff, V., & Pfäfflin, F. (2011). Transsexualism: Treatment outcome of compliant and noncompliant patients. *International Journal of Transgenderism*, *13*(1), 37–44.

Planchenault, G. (2010). Virtual community and politeness: The use of female markers of identity and solidarity in a transvestites’ website. *Journal of Politeness Research*, *6*(1), 83–103.

Plemons, E. (2013). The surgical suite. *Journal of Medical Humanities*, *34*(2), 245–247.

Pollock, L., & Eyre, S. L. (2012). Growth into manhood: Identity development among female-to-male transgender youth. *Culture, Health & Sexuality*, *14*(2), 209–222.

Poompruek, P., Boonmongkon, P., & Guadamuz, T. E. (2014). “For me ... it’s a miracle”: Injecting beauty among kathoeis in a provincial Thai city. *International Journal of Drug Policy*, *25*(4), 798–803.

Porch, M., Stukalin, R., & Weisbrod, H. (2014). Complex cases in community mental health: Stories from the Castro and the Tenderloin. *Journal of Gay & Lesbian Mental Health*, *18*(4), 393–411.

Porter, K. E., Ronneberg, C. R., & Witten, T. M. (2013). Religious affiliation and successful aging among transgender older adults: Findings from the trans Metlife survey. *Journal of Religion, Spirituality & Aging*, *25*(2), 112–138.

Prabawanti, C., Bollen, L., Palupy, R., Morineau, G., Girault, P., Mustikawati, D. E., ... & Magnani, R. (2011). HIV, sexually transmitted infections, and sexual risk behavior among transgenders in Indonesia. *AIDS and Behavior*, *15*(3), 663–673.

Prabawanti, C., Dijkstra, A., Riono, P., & Tb, G. H. (2014). Preparatory behaviours and condom use during receptive and insertive anal sex among male-to-female transgenders (waria) in Jakarta, Indonesia. *Journal of the International AIDS Society*, *17*(1), 19343. doi:[10.7448/IAS.17.1.19343](https://dx.doi.org/10.7448%2FIAS.17.1.19343)

Prinsloo, J. (2011). Negotiating transgender identities on the internet- A South African study. *Agenda*, *25*(4), 30–41.

Prunas, A., Vitelli, R., Agnello, F., Curti, E., Fazzari, P., Giannini, F., … Bini, M. (2014). Defensive functioning in MtF and FtM transsexuals. *Comprehensive Psychiatry*, *55*(4), 966–971.

Puri, M., Hall, E. G., Erisman, M., & Vwich, Y. (2014). Acting strange after trying to “get numb.” *Current Psychiatry*, *13*(11), 50–54.

Rachlin, K., Hansbury, G., & Pardo, S. T. (2010). Hysterectomy and oophorectomy experiences of female-to-male transgender individuals. *International Journal of Transgenderism*, *12*(3), 155–166.

Raigosa, M., Avvedimento, S., & Fontdevila, J. (2013). Self-made compressive dressing for vaginoplasty. *Aesthetic Plastic Surgery*, *37*(4), 844-845.

Rametti, G., Carrillo, B., Gómez-Gil, E., Junque, C., Segovia, S., Gomez, Á., & Guillamon, A. (2011a). White matter microstructure in female to male transsexuals before cross-sex hormonal treatment. A diffusion tensor imaging study. *Journal of Psychiatric Research*, *45*(2), 199–204.

Rametti, G., Carrillo, B., Gómez-Gil, E., Junque, C., Zubiarre-Elorza, L., Segovia, S., ... & Guillamon, A. (2011b). The microstructure of white matter in male to female transsexuals before cross-sex hormonal treatment. A DTI study. *Journal of Psychiatric Research*, *45*(7), 949–954.

Rametti, G., Carrillo, B., Gómez-Gil, E., Junque, C., Zubiaurre-Elorza, L., Segovia, S., ... & Guillamon, A. (2012). Effects of androgenization on the white matter microstructure of female-to-male transsexuals. A diffusion tensor imaging study. *Psychoneuroendocrinology*, *37*(8), 1261–1269.

Rankin, S., & Beemyn, G. (2012). Beyond a binary: The lives of gender-nonconforming youth. *About Campus*, *17*(4), 2–10.

Rapues, J., Wilson, E. C., Packer, T., Colfax, G. N., & Raymond, H. F. (2013). Correlates of HIV infection among transfemales, San Francisco, 2010: Results from a respondent-driven sampling study. *American Journal of Public Health*, *103*(8), 1485–1492.

Reback, C., & Fletcher, J. (2014). HIV prevalence, substance use, and sexual risk behaviors among transgender women recruited through outreach. *AIDS & Behavior*, *18*(7), 1359–1367.

Reback, C. J., Shoptaw, S., & Downing, M. J. (2012). Prevention case management improves socioeconomic standing and reduces symptoms of psychological and emotional distress among transgender women. *AIDS Care*, *24*(9), 1136–1144.

Reed, H. M. (2011). Aesthetic and functional male to female genital and perineal surgery: Feminizing vaginoplasty. *Seminars in Plastic Surgery*, *25*(2), 163–174.

Rehan, N. (2011). Genital examination of hijras. *Journal of the Pakistan Medical Association*, *61*(7), 695–696.

Reicherzer, S., & Patton, J. (2012a). Transsexual drag entertainers as keepers of queer power-knowledge. *Electronic Journal of Human Sexuality*, *15*. Retrieved from <http://mail.ejhs.org/volume15/Drag.html>

Reicherzer, S., & Spillman, J. (2012b). A multiple case study examination of resiliency factors for Mexican and Mexican-American transsexual women. *International Journal of Transgenderism*, *13*(3), 147–164.

Reinsmith-Jones, K. (2013). Transsexualism as a model of spiritual transformation: Implications. *Journal of GLBT Family Studies*, *9*(1), 65–99.

Reisner, S. L., Bailey, Z., & Sevelius, J. (2014a). Racial/ethnic disparities in history of incarceration, experiences of victimization, and associated health indicators among transgender women in the U.S. *Women & Health*, *54*(8), 750–767.

Reisner, S. L., Gamarel, K. E., Dunham, E., Hopwood, R., & Hwahng, S. (2013). Female-to-male transmasculine adult health: A mixed-methods community-based needs assessment. *Journal of the American Psychiatric Nurses Association*, *19*(5), 293–303.

Reisner, S. L., Gamarel, K. E, Nemoto, T., & Operario, D. (2014b). Dyadic effects of gender minority stressors in substance use behaviors among transgender women and their non-transgender male partners. *Psychology of Sexual Orientation and Gender Diversity*, *1*(1), 63–71.

Reisner, S. L., Perkovich, B., & Mimiaga, M. J. (2010). A mixed methods study of the sexual health needs of New England transmen who have sex with nontransgender men. *AIDS Patient Care and STDs*, *24*(8), 501–513.

Reisner, S. L., White, J. M., Bradford, J. B., & Mimiaga, M. J. (2014c). Transgender health disparities: Comparing full cohort and nested matched-pair study designs in a community health center. *LGBT Health*, *1*(3), 177–184.

Reisner, S. L., White, J. M., Mayer, K. H., & Mimiaga, M. J. (2014d). Sexual risk behaviors and psychosocial health concerns of female-to-male transgender men screening for STDs at an urban community health center. *AIDS Care*, *26*(7), 857–864.

Remacle, M., Matar, N., Morsomme, D., Veduyckt, I., & Lawson, G. (2011). Glottoplasty for male-to-female transsexualism: Voice results. *Journal of Voice*, *25*(1), 120–123.

Repessé, X., Au, S.-M., Charron, C., & Vieillard-Baron, A. (2013). Kaposi’s sarcoma: A reversible cause of ARDS in HIV-infected patient. *Intensive Care Medicine*, *39*(6), 1134–1135.

Rezwan, N., Abdel, B. A., & Andrews, H. (2014). Bilateral ureteric obstruction: An unusual complication of male-to-female gender reassignment surgery. *BMJ Case Reports*, bcr2014204894. doi:[10.1136/bcr-2014-204894](https://dx.doi.org/10.1136%2Fbcr-2014-204894)

Richards, J. T. (2013). Giving voice to the trans community on GID reform in the “DSM-5”: A Saskatchewan perspective. *Canadian Journal of Counselling and Psychotherapy*, *47*(1), 71–87.

Riggle, E. D., Rostosky, S. S., McCants, L. E., & Pascale-Hague, D. (2011). The positive aspects of a transgender self-identification. *Psychology & Sexuality*, *2*(2), 147–158.

Riggs, D. W., Coleman, K., & Due, C. (2014). Healthcare experiences of gender diverse Australians: A mixed-methods, self-report survey. *BMC Public Health*, *14*(230). doi:10.1186/1471-2458-14-230

Rijn, A. B.-V., Steensma, T. D., Kreukels, B. P. C., & Cohen-Kettenis, P. T. (2013). Self-perception in a clinical sample of gender variant children. *Clinical Child Psychology and Psychiatry*, *18*(3), 464–474.

Riley, E. A., Clemson, L., Sitharthan, G., & Diamond, M. (2013). Surviving a gender-variant childhood: The views of transgender adults on the needs of gender-variant children and their parents. *Journal of Sex & Marital Therapy*, *39*(3), 241–263.

Roberts, T. K., Kraft, C. S., French, D., Ji, W., Wu, A. H., Tangpricha, V., & Fantz, C. R. (2014). Interpreting laboratory results in transgender patients on hormone therapy. *The American Journal of Medicine*, *127*(2), 159–162.

Roerink, S., Marsman, D., van Bon, A., & Netea-Maier, R. (2014). A missed diagnosis of acromegaly during a female-to-male gender transition. *Archives of Sexual Behavior*, *43*(6), 1199–1201.

Rolle, L., Falcone, M., Vighetti, S., Ceruti, C., Sedigh, O., Timpano, M., ... & Frea, B. (2014). Does sex reassignment surgery induce cerebral modifications in MTF transsexuals? *Journal of Sexual Medicine*, *11*(1), 312–312.

Rooke, A. (2010). Trans youth, science and art: Creating (trans) gendered space. *Gender, Place and Culture*, *17*(5), 655–672.

Rosiek, J. L., & Heffernan, J. (2014). Can’t code what the community can’t see: A case of the erasure of heteronormative harassment. *Qualitative Inquiry*, *20*(6), 726–733.

Ross, B. L. (2012). Outdoor brothel culture: The un/making of a transsexual stroll in Vancouver’s West End, 1975-1984. *Journal of Historical Sociology*, *25*(1), 126–150.

Rotondi, N., Bauer, G., Scanlon, K., Kaay, M., Travers, R., & Travers, A. (2011a). Prevalence of and risk and protective factors for depression in female-to-male transgender Ontarians: Trans PULSE project. *Canadian Journal of Community Mental Health*, *30*(2), 135–155.

Rotondi, N. K., Bauer, G. R., Scanlon, K., Kaay, M., Travers, R., & Travers, A. (2013). Nonprescribed hormone use and self-performed surgeries: “Do-it-yourself” transitions in transgender communities in Ontario, Canada. *American Journal of Public Health*, *103*(10), 1830–1836.

Rotondi, N. K., Bauer, G. R., Travers, R., Travers, A., Scanlon, K., & Kaay, M. (2011b). Depression in male-to-female transgender Ontarians: Results from the Trans PULSE Project. *Canadian Journal of Community Mental Health*, *30*(2), 113–133.

Rowniak, S., & Chesla, C. (2013). Coming out for a third time: Transmen, sexual orientation, and identity. *Archives of Sexual Behavior*, *42*(3), 449–461.

Rowniak, S., Chesla, C., Rose, C. D., & Holzemer, W. L. (2011). Transmen: The HIV risk of gay identity. *AIDS Education and Prevention*, *23*(6), 508–520.

Rupp, L. J., Taylor, V., & Shapiro, E. I. (2010). Drag queens and drag kings: The difference gender makes. *Sexualities*, *13*(3), 275–294.

Sahastrabuddhe, S., Gupta, A., Stuart, E., Godbole, S., Ghate, M., Sahay, S., … Mehendale, S. M. (2012). Sexually transmitted infections and risk behaviors among transgender persons (Hijras) of Pune, India. *Journal of Acquired Immune Deficiency Syndromes*, *59*(1), 72–78.

Saketopoulou, A. (2011). Minding the gap: Intersections between gender, race, and class in work with gender variant children. *Psychoanalytic Dialogues*, *21*(2), 192–209.

Saketopoulou, A. (2014). Mourning the body as bedrock: Developmental considerations in treating transsexual patients analytically. *Journal of the American Psychoanalytic Association*, *62*(5), 773–806.

Saltzburg, S., & Davis, T. S. (2010). Co-authoring gender-queer youth identities: Discursive tellings and retellings. *Journal of Ethnic & Cultural Diversity in Social Work*, *19*(2), 87–108.

Salvador, J., Massuda, R., Andreazza, T., Koff, W. J., Silveira, E., Kreische, F., ... & Lobato, M. I. R. (2012). Minimum 2‐year follow up of sex reassignment surgery in Brazilian male‐to‐female transsexuals. *Psychiatry and Clinical Neurosciences*, *66*(4), 371–372.

Samkhaniyani, E., Khalatbari, J., & Arkiyan, F. (2013). The relationship between the disconnection and rejection domain of early maladaptive schemas with defense mechanisms in individuals with gender identity disorder. *Life Science Journal*, *10*(S1), 436–440.

Santarnecchi, E., Vatti, G., Déttore, D., & Rossi, A. (2012). Intrinsic cerebral connectivity analysis in an untreated female-to-male transsexual subject: A first attempt using resting-state fMRI. *Neuroendocrinology*, *96*(3), 188–193.

Santos, G. M., Rapues, J., Wilson, E. C., Macias, O., Packer, T., Colfax, G., & Raymond, H. F. (2014a). Alcohol and substance use among transgender women in San Francisco: Prevalence and association with human immunodeficiency virus infection. *Drug & Alcohol Review*, *33*(3), 287–295.

Santos, G. M., Wilson, E. C., Rapues, J., Macias, O., Packer, T., & Raymond, H. F. (2014b). HIV treatment cascade among transgender women in a San Francisco respondent driven sampling study. *Sexually Transmitted Infections*, *90*(5), 430–433.

Saravanan, N., Thiruneervannan, R., & Christopher, P. (2014). A study to assess the periodontal status of transgender in Chennai city. *Biosciences Biotechnology Research Asia*, *11*(3), 1673–1678.

Sarrau, M., Casoli, V., & Weigert, R. (2014). Successful conservative management of traumatic post-coital recto-neovaginal fistula in male-to-female transsexual. *Journal of Obstetrics and Gynaecology*, *34*(8), 747–749.

Saunders, K., & Bass, C. (2011). Gender reassignment: 5 years of referrals in Oxfordshire. *The Psychiatrist*, *35*(9), 325–327.

Schagen, S. E., Delemarre-van de Waal, H. A., Blanchard, R., & Cohen-Kettenis, P. T. (2012). Sibling sex ratio and birth order in early-onset gender dysphoric adolescents. *Archives of Sexual Behavior*, *41*(3), 541–549.

Schenck, T. L., Holzbach, T., Zantl, N., Schuhmacher, C., Vogel, M., Seidl, S., ... & Giunta, R. E. (2010). Vaginal Carcinoma in a Female-to-Male Transsexual. *Journal of Sexual Medicine*, *7*(8), 2899–2902.

Schilt, K., & Windsor, E. (2014). The sexual habitus of transgender men: Negotiating sexuality through gender. *Journal of Homosexuality*, *61*(5), 732–748.

Schöning, S., Engelien, A., Bauer, C., Kugel, H., Kersting, A., Roestel, C., … Konrad, C. (2010). Neuroimaging differences in spatial cognition between men and male-to-female transsexuals before and during hormone therapy. *Journal of Sexual Medicine*, *7*(5), 1858–1867.

Schor, E. (2011). Irwin Keller: A performer of parts. *TDR: The Drama Review*, *55*(3), 80–89.

Schwartz, S. W. (2010). Martha at Martha: A seance with Richard Move. *Women and Performance*, *20*(1), 61–87.

Seal, L. J., Franklin, S., Richards, C., Shishkareva, A., Sinclaire, C., & Barrett, J. (2012). Predictive markers for mammoplasty and a comparison of side effect profiles in transwomen taking various hormonal regimens. *Journal of Clinical Endocrinology and Metabolism*, *97*(12), 4422–4428.

Seelman, K. L. (2014a). Recommendations of transgender students, staff, and faculty in the USA for improving college campuses. *Gender and Education*, *26*(6), 618–635.

Seelman, K. L. (2014b). Transgender individuals’ access to college housing and bathrooms: Findings from the national transgender discrimination survey. *Journal of Gay & Lesbian Social Services*, *26*(2), 186–206.

Seemanthini, T. S., & Manjula, M. Y. (2011). Personality and emotional intelligence of transsexuals. *Journal of Psychosocial Research*, *6*(2), 179–185.

Sevelius, J. M. (2013). Gender affirmation: A framework for conceptualizing risk behavior among transgender women of color. *Sex Roles*, *68*(11-12), 675–689.

Sevelius, J. M., Carrico, A., & Johnson, M. O. (2010). Antiretroviral therapy adherence among transgender women living with HIV. *JANAC: Journal of the Association of Nurses in AIDS Care*, *21*(3), 256–264.

Sevelius, J. M., Patouhas, E., Keatley, J. G., & Johnson, M. O. (2014a). Barriers and facilitators to engagement and retention in care among transgender women living with human immunodeficiency virus. *Annals of Behavioral Medicine*, *47*(1), 5–16.

Sevelius J. M., Saberi, P., & Johnson, M. O. (2014b). Correlates of antiretroviral adherence and viral load among transgender women living with HIV. *AIDS Care*, *26*(8), 976–982.

Sexton, L., Jenness, V., & Sumner, J. M. (2010). Where the margins meet: A demographic assessment of transgender inmates in men’s prisons. *Justice Quarterly*, *27*(6), 835–866.

Shao, T., Grossbard, M. L., & Klein, P. (2011). Breast cancer in female-to-male transsexuals: Two cases with a review of physiology and management. *Clinical Breast Cancer*, *11*(6), 417–419.

Shepard, B. (2013). From community organization to direct services: The street trans action revolutionaries to Sylvia Rivera Law Project. *Journal of Social Service Research*, *39*(1), 95–114.

Shipherd, J. C., Green, K. E., & Abramovitz, S. (2010). Transgender clients: Identifying and minimizing barriers to mental health treatment. *Journal of Gay & Lesbian Mental Health*, *14*(2), 94–108.

Shipherd, J. C., Maguen, S., Skidmore, W. C., & Abramovitz, S. M. (2011). Potentially traumatic events in a transgender sample: frequency and associated symptoms. *Traumatology*, *17*(2), 56–67.

Shipherd, J. C., Mizock, L., Maguen, S., & Green, K. E. (2012). Male-to-female transgender veterans and VA health care utilization. *International Journal of Sexual Health*, *24*(1), 78–87.

Shrestha, R. K., Sansom, S. L., Schulden, J. D., Song, B., Smith, L. C., Ramirez, R., ... & Heffelfinger, J. D. (2011). Costs and effectiveness of finding new HIV diagnoses by using rapid testing in transgender communities. *AIDS Education and Prevention*, *23*(S3), 49–57.

Shvartsbeyn, M., & Rapkiewicz, A. (2011). Silicon-associated subcutaneous lesion presenting as a mass: A confounding histopathologic correlation. *Human Pathology*, *42*(9), 1364–1367.

Silva-Santisteban, A., Raymond, H. F., Salazar, X., Villayzan, J., Leon, S., McFarland, W., & Caceres, C. F. (2012). Understanding the HIV/AIDS epidemic in transgender women of Lima, Peru: Results from a sero-epidemiologic study using respondent driven sampling. *AIDS & Behavior*, *16*(4), 872–881.

Simon, L., Kozak, L. R., Simon, V., Czobor, P., Unoka, Z., Szabo, A., & Csukly, G. (2013a). Regional grey matter structure differences between transsexuals and healthy controls- A voxel based morphometry study. *PloS One*, *8*(12), e83947. doi:[10.1371/journal.pone.0083947](https://doi.org/10.1371/journal.pone.0083947)

Simon, L., Zsolt, U., Fogd, D., & Czobor, P. (2011). Dysfunctional core beliefs, perceived parenting behavior and psychopathology in gender identity disorder: A comparison of male-to-female, female-to-male transsexual and nontranssexual control subjects. *Journal of Behavior Therapy and Experimental Psychiatry*, *42*(1), 38–45.

Simons, L., Schrager, S. M., Clark, L. F., Belzer, M., & Olson, J. (2013b). Parental support and mental health among transgender adolescents. *Journal of Adolescent Health*, *53*(6), 791–793.

Singh, A. A. (2013a). Transgender youth of color and resilience: Negotiating oppression and finding support. *Sex Roles*, *68*(11-12), 690–702.

Singh, A. A, Hays, D. G., & Watson, L. S. (2011a). Strength in the face of adversity: Resilience strategies of transgender individuals. *Journal of Counseling & Development*, *89*(1), 20–27.

Singh, A. A., & McKleroy, V. S. (2011b). “Just getting out of bed is a revolutionary act”: The resilience of transgender people of color who have survived traumatic life events. *Traumatology*, *17*(2), 34–44.

Singh, A. A., Meng, S., & Hansen, A. (2013b). “It’s already hard enough being a student”: Developing affirming college environments for trans youth. *Journal of LGBT Youth*, *10*(3), 208–223.

Singh, A. A., Meng, S. E., & Hansen, A. W. (2014). “I am my own gender”: Resilience strategies of trans youth. *Journal of Counseling & Development*, *92*(2), 208–218.

Singh, D., Deogracias, J. J., Johnson, L. L., Bradley, S. J., Kibblewhite, S. J., Owen-Anderson, A., ... & Zucker, K. J. (2010). The Gender Identity/Gender Dysphoria Questionnaire for Adolescents and Adults: Further validity evidence. *Journal of Sex Research*, *47*(1), 49–58.

Sitek, A., Fijałkowska, M., Żądzińska, E., & Antoszewski, B. (2012). Biometric characteristics of the pelvis in female-to-male transsexuals. *Archives of Sexual Behavior*, *41*(5), 1303–1313.

Siverskog, A. (2014). “They just don’t have a clue”: Transgender aging and implications for social work. *Journal of Gerontological Social Work*, *57*(2-4), 386–406.

Skagerberg, E., Davidson, S., & Carmichael, P. (2013a). Internalizing and externalizing behaviors in a group of young people with gender dysphoria. *International Journal of Transgenderism*, *14*(3), 105–112.

Skagerberg, E., Parkinson, R., & Carmichael, P. (2013b). Self-harming thoughts and behaviors in a group of children and adolescents with gender dysphoria. *International Journal of Transgenderism*, *14*(2), 86–92.

Skugarevsky, O., Ehrlich, E., & Sheleg, S. (2011). Accidental strangulation resulted from hypoxyphilia associated with multiple paraphilias and substance abuse: A psychological autopsy case report. *Romanian Journal of Legal Medicine*, *19*(4), 249–252.

Socias, M., Marshall, B., Aristegui, I., Romero, M., Cahn, P., Kerr, T., & Sued, O. (2014a). Factors associated with healthcare avoidance among transgender women in Argentina. *International Journal for Equity in Health*, *13*(81). doi:10.1186/s12939-014-0081-7

Socias, M. E., Marshall, B. D., Aristegui, I., Zalazar, V., Romero, M., Sued, O., & Kerr, T. (2014b). Towards full citizenship: Correlates of engagement with the gender identity law among transwomen in Argentina. *PloS One*, *9*(8), e105402. doi:[10.1371/journal.pone.0105402](https://doi.org/10.1371/journal.pone.0105402)

Soleman, R. S., Staphorsius, A. S., Cohen-Kettenis, P. T., Lambalk, C. B., Veltman, D. J., van Trotsenburg, M. A., … Kreukels, B. P. (2014). Oestrogens are not related to emotional processing: A study of regional brain activity in female-to-male transsexuals under gonadal suppression. *Cerebral Cortex*, *26*(2), 510–516.

Soley-Beltran, P., & Coll-Planas, G. (2011). “Having words for everything”. Institutionalizing gender migration in Spain (1998-2008). *Sexualities*, *14*(3), 334–353.

Song, C., Wong, M., Wong, C. H., & Ong, Y. S. (2011). Modifications of the radial forearm flap phalloplasty for female-to-male gender reassignment. *Journal of Reconstructive Microsurgery*, *27*(2), 115–120.

Spack, N. P. (2013). Management of transgenderism. *JAMA: Journal of the American Medical Association*, *309*(5), 478–484.

Spack, N. P., Edwards-Leeper, L., Feldman, H. A., Leibowitz, S., Mandel, F., Diamond, D. A., & Vance, S. R. (2012). Children and adolescents with gender identity disorder referred to a pediatric medical center. *Pediatrics*, *129*(3), 418–425.

Speer, S. A., & McPhillips, R. (2013). Patients’ perspectives on psychiatric consultations in the Gender Identity Clinic: Implications for patient-centered communication. *Patient Education and Counseling*, *91*(3), 385–391.

St Peter, M., Trinidad, A., & Irwig, M. S. (2012). Self-castration by a transsexual woman: Financial and psychological costs: A case report. *Journal of Sexual Medicine*, *9*(4), 1216–1219.

Steensma, T. D., Biemond, R., de Boer, F., & Cohen-Kettenis, P. T. (2011). Desisting and persisting gender dysphoria after childhood: A qualitative follow-up study. *Clinical Child Psychology and Psychiatry*, *16*(4), 499–516.

Steensma, T. D., McGuire, J. K., Kreukels, B. P., Beekman, A. J., & Cohen-Kettenis, P. T. (2013). Factors associated with desistence and persistence of childhood gender dysphoria: A quantitative follow-up study. *Journal of the American Academy of Child & Adolescent Psychiatry*, *52*(6), 582–590.

Steensma, T. D., Zucker, K. J., Kreukels, B. P. C., Vanderlaan, D. P., Wood, H., Fuentes, A., & Cohen-Kettenis, P. T. (2014). Behavioral and emotional problems on the teacher’s report form: A cross-national, cross-clinic comparative analysis of gender dysphoric children and adolescents. *Journal of Abnormal Child Psychology*, *42*(4), 635–647.

Stephens, S. C., Bernstein, K. T., & Philip, S. S. (2011). Male to female and female to male transgender persons have different sexual risk behaviors yet similar rates of STDs and HIV. *AIDS and Behavior*, *15*(3), 683–686.

Stotzer, R. L. (2011). Family cohesion among Hawai’i’s Māhūwahine. *Journal of GLBT Family Studies*, *7*(5), 424–435.

Stotzer, R. L. (2014). Law enforcement and criminal justice personnel interactions with transgender people in the United States: A literature review. *Aggression and Violent Behavior*, *19*(3), 263–277.

Strain, J. D., & Shuff, I. M. (2011). Psychological well-being and level of outness in a population of male-to-female transsexual women attending a national transgender conference. *International Journal of Transgenderism*, *12*(4), 230–240.

Suchet, M. (2011). Crossing over. *Psychoanalytic Dialogues*, *21*(2), 172–191.

Sultana, A., & Kalyani, M. K. (2012). Femaling males: Anthropological analysis of the transgender community in Pakistan. *Journal of Humanities and Social Sciences*, *20*(1), 93–108.

Summers, S. M., & Onate, J. (2014). New onset psychosis following abrupt discontinuation of hormone replacement therapy in a trans woman. *Journal of Gay & Lesbian Mental Health*, *18*(3), 312–319.

Syed, M. A., & Abdul, G. K. (2013). Gender identity disorder is not simply two in one. *International Medical Journal Malaysia*, *12*(2), 83–85.

Tagg, B. (2012). Transgender netballers: Ethical issues and lived realities. *Sociology of Sport Journal*, *29*(2), 151–167.

Tavakkoli, T. K., Djavan, B., Hosseini, J., Ghoreifi, A., Ershadi, M., & Hosseini, E. (2014). Fold-back perineoscrotal flap plus penile inversion vaginoplasty for male-to-female gender reassignment surgery in circumcised subjects. *European Journal of Plastic Surgery,* *38*(1), 43-48.

Tayade, P. J. (2011). Transsexualism. *International Journal of Medical Toxicology and Legal Medicine*, *13*(4), 24–29.

Taylor, E. T. (2013). Transmen’s health care experiences: Ethical social work practice beyond the binary. *Journal of Gay & Lesbian Social Services*, *25*(1), 102–120.

Taylor, R. D., Bimbi, D. S., Joseph, H. A., Margolis, A. D., & Parsons, J. T. (2011). Girlfriends: Evaluation of an HIV-risk reduction intervention for adult transgender women. *AIDS Education and Prevention*, *23*(5), 469–478.

Taziaux, M., Swaab, D. F., & Bakker, J. (2012). Sex differences in the neurokinin B system in the human infundibular nucleus. *Journal of Clinical Endocrinology and Metabolism*, *97*(12), E2210–E2220.

Tchang, L. A., Largo, R. D., Babst, D., Wettstein, R., Haug, M. D., Kalbermatten, D. F., & Schaefer, D. J. (2014). Second free radial forearm flap for urethral reconstruction after partial flap necrosis of tube-in-tube phalloplasty with radial forearm flap: A report of two cases. *Microsurgery*, *34*(1), 58–63.

Ten Kulve, J. S., De Jong, F. H., & De Ronde, W. (2011). The effect of circulating estradiol concentrations on gonadotropin secretion in young and old castrated male-to-female transsexuals. *Aging Male*, *14*(3), 155–161.

Terada, S., Matsumoto, Y., Sato, T., Okabe, N., Kishimoto, Y., & Uchitomi, Y. (2011). Suicidal ideation among patients with gender identity disorder. *Psychiatry Research*, *190*(1), 159–162.

Terada, S., Matsumoto, Y., Sato, T., Okabe, N., Kishimoto, Y., & Uchitomi, Y. (2012a). Factors predicting psychiatric co-morbidity in gender-dysphoric adults. *Psychiatry Research*, *200*(2-3), 469–474.

Terada, S., Matsumoto, Y., Sato, T., Okabe, N., Kishimoto, Y., & Uchitomi, Y. (2012b). School refusal by patients with gender identity disorder. *General Hospital Psychiatry*, *34*(3), 299–303.

Testa, R. J., Habarth, J., Peta, J., Balsam, K., & Bockting, W. (2014). Development of the gender minority stress and resilience measure. *Psychology of Sexual Orientation and Gender Diversity, 2*(1), 65-77.

Testa, R. J., Jimenez, C. L., & Rankin, S. (2014). Risk and resilience during transgender identity development: The effects of awareness and engagement with other transgender people on affect. *Journal of Gay and Lesbian Mental Health*, *18*(1), 31–46.

Testa, R. J., Sciacca, L. M., Wang, F., Hendricks, M. L., Goldblum, P., Bradford, J., & Bongar, B. (2012). Effects of violence on transgender people. *Professional Psychology: Research & Practice*, *43*(5), 452–459.

Thione, A., Cavadas, P. C., & Carballeira, A. (2014). Urethra reconstruction with a prelaminated pedicled anterolateral thigh flap: A case report. *Annals of Plastic Surgery*, *72*(S2), 695–697.

Thomas, J. P., & MacMillan, C. (2013). Feminization laryngoplasty: Assessment of surgical pitch elevation. *European Archives of Oto-Rhino-Laryngology*, *270*(10), 2695–2700.

Thornhill, L., & Klein, P. (2010). Creating environments of care with transgender communities. *JANAC: Journal of the Association of Nurses in AIDS Care*, *21*(3), 230–239.

Tomada, I., Tomada, N., Almeida, H., & Neves, D. (2013). Androgen depletion in humans leads to cavernous tissue reorganization and upregulation of Sirt1-eNOS axis. *Age*, *35*(1), 35–47.

Torwane, N. A., Hongal, S., Saxena, E., Rana, P. T., Jain, S., & Gouraha, A. (2014). Assessment of periodontal status among eunuchs residing in Bhopal city, Madhya Pradesh, India: A cross-sectional study. *Oral Health and Dental Management*, *13*(3), 628–33.

Tourbach, S. A. G. J., Hunter-Smith, D., & Morrison, W. A. (2011). Long anterior urethral reconstruction using a jejunal free flap. *Journal of Plastic Surgery and Hand Surgery*, *45*(1), 54–56.

Traish, A. M., & Gooren, L. J. (2010). Safety of physiological testosterone therapy in women: Lessons from female-to-male transsexuals (FMT) treated with pharmacological testosterone therapy. *Journal of Sexual Medicine*, *7*(11), 3758–3764.

Travers, A., & Deri, J. (2011). Transgender inclusion and the changing face of lesbian softball leagues. *International Review for the Sociology of Sport*, *46*(4), 488–507.

Trevor, M., & Boddy, J. (2013). Transgenderism and Australian social work: A literature review. *Australian Social Work*, *66*(4), 555–570.

Turo, R., Jallad, S., Prescott, S., & Cross, W. R. (2013). Metastatic prostate cancer in transsexual diagnosed after three decades of estrogen therapy. *Canadian Urological Association Journal*, *7*(7-8), E544–E546.

Ung Loh, J. (2014). Narrating identity: The employment of mythological and literary narratives in identity formation among the hijras of India. *Religion & Gender*, *4*(1), 21–39.

Urban, R. R., Teng, N. N. H., & Kapp, D. S. (2011). Gynecologic malignancies in female-to-male transgender patients: The need of original gender surveillance. *American Journal of Obstetrics and Gynecology*, *204*(5), e9–e12.

Usmani, M. A., Gaur, R. K., Azmi, S. A., & Gangwar, S. (2012). Treatment of transvestic fetishism with fluoxetine: A case report. *Iranian Journal of Psychiatry and Behavioral Sciences*, *6*(2), 100–101.

Van Caenegem, E., Taes, Y., Wierckx, K., Vandewalle, S., Toye, K., Kaufman, J. M., … T’Sjoen, G. (2013a). Low bone mass is prevalent in male-to-female transsexual persons before the start of cross-sex hormonal therapy and gonadectomy. *Bone*, *54*(1), 92–97.

Van Caenegem, E., Verhaeghe, E., Taes, Y., Wierckx, K., Toye, K., Goemaere, S., ... & T'Sjoen, G. (2013b). Long-term evaluation of donor-site morbidity after radial forearm flap phalloplasty for transsexual men. *Journal of Sexual Medicine*, *10*(6), 1644–1651.

Van Caenegem, E., Wierckx, K., Taes, Y., Dedecker, D., Van de Peer, F., Toye, K., ... & T'Sjoen, G. (2012). Bone mass, bone geometry, and body composition in female-to-male transsexual persons after long-term cross-sex hormonal therapy. *The Journal of Clinical Endocrinology and Metabolism*, *97*(7), 2503–2511.

Van Devanter, N., Duncan, A., Raveis, V. H., Birnbaum, J., Burrell-Piggott, T., & Siegel, K. (2012). Continued sexual risk behaviour in African American and Latino male-to-female transgender adolescents living with HIV/AIDS: A case study. *Journal of AIDS & Clinical Research*. Retrieved from <https://www.ncbi.nlm.nih.gov/pmc/articles/PMC3478681/pdf/nihms364030.pdf>

Vanderlaan, D. P., & Vasey, P. L. (2011). Male sexual orientation in Independent Samoa: Evidence for fraternal birth order and maternal fecundity effects. *Archives of Sexual Behavior*, *40*(3), 495–503.

VanderLaan, D. P., Vokey, J. R., & Vasey, P. L. (2013). Is transgendered male androphilia familial in non-western populations? The case of a Samoan village. *Archives of Sexual Behavior*, *42*(3), 361–370.

VanKim, N. A., Erickson, D. J., Eisenberg, M. E., Lust, K., Rosser, B. R., & Laska, M. N. (2014). Weight-related disparities for transgender college students. *Health Behavior and Policy Review*, *1*(2), 161–171.

Vasey, P. L., & VanderLaan, D. P. (2010). Avuncular tendencies and the evolution of male androphilia in Samoan fa’afafine. *Archives of Sexual Behavior*, *39*(4), 821–830.

Veale, J. (2014). Evidence against a typology: A taxometric analysis of the sexuality of male-to-female transsexuals. *Archives of Sexual Behavior*, *43*(6), 1177–1186.

Veale, J. F., Clarke, D. E., & Lomax, T. C. (2012). Male-to-female transsexuals’ impressions of Blanchard’s autogynephilia theory. *International Journal of Transgenderism*, *13*(3), 131–139.

Vegter, V. (2013). Conceptualizing masculinity in female-to-male trans-identified individuals: A qualitative inquiry. *Canadian Journal of Counselling and Psychotherapy*, *47*(1), 88–108.

Velayudhan, R., Khaleel, A., Sankar, N., Kumar, M., Kazhungil, F., & Raghuram, T. M. (2014). Fetishistic transvestism in a patient with mental retardation and psychosis. *Indian Journal of Psychological Medicine*, *36*(2), 198–200.

Verma, S. K., Shukla, A, & Bharti, P. (2011). The Indian eunuch: An efficient emotional labour. *Eastern Anthropologist*, *64*(2-3), 251–261.

Victor, V. M., Rocha, M., Bañuls, C., Rovira‐Llopis, S., Gómez, M., & Hernández‐Mijares, A. (2014). Mitochondrial impairment and oxidative stress in leukocytes after testosterone administration to female-to-male transsexuals. *Journal of Sexual Medicine*, *11*(2), 454–461.

Vigneswaran, N., Lim, J., Lee, H. J., Ong, W. C., Rasheed, M. Z., & Lim, T. C. (2013). A novel technique with aesthetic considerations in female-to-male transsexuals nipple areola complex reconstruction. *Journal of Plastic, Reconstructive & Aesthetic Surgery : JPRAS*, *66*(12), 1805–1807.

Vinay, B., Krishna, P. M., Suresh, K., & Srikala, B. (2010). Transsexualism in the Indian context. *Journal of Indian Association for Child and Adolescent Mental Health*, *6*(2), 44–46.

Visnyei, K., Samuel, M., Heacock, L., & Cortes, J. A. (2014). Hypercalcemia in a male-to-female transgender patient after body contouring injections: A case report. *Journal of Medical Case Reports*, *8*(1), 1–14.

Vitelli, R., & Riccardi, E. (2010). Gender identity disorder and attachment theory: The influence of the patient’s internal working models on psychotherapeutic engagement and objective. A study undertaken using the Adult Attachment Interview. *International Journal of Transgenderism*, *12*(4), 241–253.

Vivek, P. S. (2013). Sexual accident: Hijra community in Mumbai. *Eastern Anthropologist*, *66*(2-3), 329–344.

Vujović, S., Popović, S. Marojević, L. M., Ivović, M., Tančić-Gajić, M., Stojanović, M., ... & Duišin, D. (2014). Finger length ratios in Serbian transsexuals. *Scientific World Journal*, 763563*.* doi:[10.1155/2014/763563](http://dx.doi.org/10.1155/2014/763563)

Vukadinovic, V., Stojanovic, B., Majstorovic, M., & Milosevic, A. (2014). The role of clitoral anatomy in female to male sex reassignment surgery. *Scientific World Journal*, 437378. doi:[10.1155/2014/437378](http://dx.doi.org/10.1155/2014/437378)

Wagner, S., Greco, F., Hoda, M. R., Inferrera, A., Lupo, A., Hamza, A., & Fornara, P. (2010). Male-to-female transsexualism: Technique, results and 3-year follow-up in 50 patients. *Urologia Internationalis*, *84*(3), 330–333.

Walinsky, D., & Whitcomb, D. (2010). Using the ACA Competencies for counseling with transgender clients to increase rural transgender well-being. *Journal of LGBT Issues in Counseling*, *4*(3-4), 160–175.

Wallace, P. M. (2010a). Finding self: A qualitative study of transgender, transitioning, and adulterated silicone. *Health Education Journal*, *69*(4), 439–446.

Wallace, P. M., & Rasmussen, S. (2010b). Analysis of adulterated silicone: Implications for health promotion. *International Journal of Transgenderism*, *12*(3), 167–175.

Wallace, S. A., Blough, K. L., & Kondapalli, L. A. (2014). Fertility preservation in the transgender patient: Expanding oncofertility care beyond cancer. *Gynecological Endocrinology*, *30*(12), 868–871.

Wallien, M. S., Veenstra, R., Kreukels, B. P., & Cohen-Kettenis, P. T. (2010). Peer group status of gender dysphoric children: A sociometric study. *Archives of Sexual Behavior*, *39*(2), 553–560.

Walsh, C. M., Yang, L., Park, J. M., Askeland, R. W., & Fajardo, L. L. (2014). Angiolipoma of the breast in a transgender patient. *Breast Journal*, *20*(6), 662–663.

Weigert, R., Frison, E., Sessiecq, Q., Al Mutairi, K., & Casoli, V. (2013). Patient satisfaction with breasts and psychosocial, sexual, and physical well-being after breast augmentation in male-to-female transsexuals. *Plastic and Reconstructive Surgery*, *132*(6), 1421–1429.

Weyers, S., De Sutter, P., Hoebeke, S., Monstrey, G., T’Sjoen, G., Verstraelen, H., & Gerris, J. (2010a). Gynaecological aspects of the treatment and follow-up of transsexual men and women. *Facts, Views & Vision in ObGyn*, *2*(1), 35–54.

Weyers, S., Lambein, K., Sturtewagen, Y., Verstraelen, H., Gerris, J., & Praet, M. (2010b). Cytology of the “penile” neovagina in transsexual women. *Cytopathology*, *21*(2), 111–115.

Weyers, S., Villeirs, G., Vanherreweghe, E., Verstraelen, H., Monstrey, S., Van den Broecke, R., & Gerris, J. (2010c). Mammography and breast sonography in transsexual women. *European Journal of Radiology*, *74*(3), 508–513.

Wierckx, K., De Zaeytijd, J., Elaut, E., Heylens, G., & T’Sjoen, G. Wierckx, K., ... & T’Sjoen, G. (2014a). Bilateral non-arteritic ischemic optic neuropathy in a transsexual woman using excessive estrogen dosage. *Archives of Sexual Behavior*, *43*(2), 407–409.

Wierckx, K., Elaut, E., Declercq, E., Heylens, G., De Cuypere, G., Taes, Y., … T’Sjoen, G. (2013). Prevalence of cardiovascular disease and cancer during cross-sex hormone therapy in a large cohort of trans persons: A case-control study. *European Journal of Endocrinology*, *169*(4), 471–478.

Wierckx, K., Elaut, E., Van Caenegem, E., Van De Peer, F., Dedecker, D., Van Houdenhove, E., & T’Sjoen, G. (2011a). Sexual desire in female-to-male transsexual persons: Exploration of the role of testosterone administration. *European Journal of Endocrinology*, *165*(2), 331–337.

Wierckx, K., Elaut, E., Van Hoorde, B., Heylens, G., De Cuypere, G., Monstrey, S., … T’Sjoen, G. (2014b). Sexual desire in trans persons: Associations with sex reassignment treatment. *Journal of Sexual Medicine*, *11*(1), 107–118.

Wierckx, K., Gooren, L., & T’Sjoen, G. (2014c). Clinical review: Breast development in trans women receiving cross-sex hormones. *Journal of Sexual Medicine*, *11*(5), 1240–1247.

Wierckx, K., Mueller, S., Weyers, S., Van Caenegem, E., Roef, G., Heylens, G., & T’Sjoen, G. (2012a). Long-term evaluation of cross-sex hormone treatment in transsexual persons. *Journal of Sexual Medicine*, *9*(10), 2641–2651.

Wierckx, K., Stuyver, I., Weyers, S., Hamada, A., Agarwal, A., De Sutter, P., & T’Sjoen, G. (2012b). Sperm freezing in transsexual women. *Archives of Sexual Behavior*, *41*(5), 1069–1071.

Wierckx, K., Van Caenegem, E., Elaut, E., Dedecker, D., Van de Peer, F., Toye, K., ... & T'Sjoen, G. (2011b). Quality of life and sexual health after sex reassignment surgery in transsexual men. *Journal of Sexual Medicine*, *8*(12), 3379–3388.

Wierckx, K., Van Caenegem, E., Pennings, G., Elaut, E., Dedecker, D., Van De Peer, F., … T’Sjoen, G. (2012c). Reproductive wish in transsexual men. *Human Reproduction*, *27*(2), 483–487.

Wierckx, K., Van Caenegem, E., Schreiner, T., Haraldsen, I., Fisher, A., Toye, K., ... & T'Sjoen, G. (2014d). Cross-sex hormone therapy in trans persons is safe and effective at short-time follow-up: Results from the European network for the investigation of gender incongruence. *Journal of Sexual Medicine*, *11*(8), 1999–2011.

Wierckx, K., Van de Peer, F., Verhaeghe, E., Dedecker, D., Van Caenegem, E., Toye, K., … T’Sjoen, G. (2014e). Short- and long-term clinical skin effects of testosterone treatment in trans men. *Journal of Sexual Medicine*, *11*(1), 222–229.

Wight, J. (2014). Queer sweet home: Disorientation, tyranny, and silence in digital space. *Cultural Studies - Critical Methodologies*, *14*(2), 128–137.

Williams, A. R. (2012). Case discussions: Transsexualism, personality disorders, and spinal cord injury. *Journal of Gay & Lesbian Mental Health*, *16*(1), 56–65.

Williams, C. J, Weinberg, M. S., & Rosenberger, J. G. (2013). Trans men: Embodiments, identities, and sexualities. *Sociological Forum*, *28*(4), 719–741.

Wilson, D., Marais, A., De Villiers, A., Addinall, R., & Campbell, M. M. (2014). Transgender issues in South Africa, with particular reference to the Groote Schuur Hospital Transgender Unit. *South African Medical Journal*, *104*(6), 449-451.

Wilson, E., Pant, S. B., Comfort, M, & Ekstrand, M. (2011). Stigma and HIV risk among Metis in Nepal. *Culture, Health & Sexuality*, *13*(3), 253–266.

Wilson, E., Rapues, J., Jin, H., & Raymond, H. F. (2014). The use and correlates of illicit silicone or “fillers” in a population-based sample of transwomen, San Francisco, 2013. *Journal of Sexual Medicine*, *11*(7), 1717–1724.

Wilson, E. C, Arayasirikul, S., & Johnson, K. (2013). Access to HIV care and support services for African American transwomen living with HIV. *International Journal of Transgenderism*, *14*(4), 182–195.

Wilson, E. C., Garofalo, R., Harris, D. R., & Belzer, M. (2010a). Sexual risk taking among transgender male-to-female youths with different partner types. *American Journal of Public Health*, *100*(8), 1500–1505.

Wilson, E. C., Iverson, E., Garofalo, R., & Belzer, M. (2012). Parental support and condom use among transgender female youth. *JANAC: Journal of the Association of Nurses in AIDS Care*, *23*(4), 306–317.

Wilson, E. C., Santos, G. M., & Raymond, H. F. (2014b). Sexual mixing and the risk environment of sexually active transgender women: Data from a respondent-driven sampling study of HIV risk among transwomen in San Francisco, 2010. *BMC Infectious Diseases*, *14*(1), 430–438.

Winograd, W. (2014). The wish to be a boy: Gender dysphoria and identity confusion in a self-identified transgender adolescent. *Psychoanalytic Social Work*, *21*(1-2), 55–74.

Witten, T. M. (2014). End of life, chronic illness, and trans-identities. *Journal of Social Work in End-of-Life & Palliative Care*, *10*(1), 34–58.

Wood, E., & Halder, N. (2014). Gender disorders in learning disability- A systematic review. *Tizard Learning Disability Review*, *19*(4), 158–165.

Wood, H., Sasaki, S., Bradley, S. J., Singh, D., Fantus, S., Owen-Anderson, A., ... & Zucker, K. J. (2013). Patterns of referral to a gender identity service for children and adolescents (1976-2011): Age, sex ratio, and sexual orientation. *Journal of Sex & Marital Therapy*, *39*(1), 1–6.

Woods, J. B., Galvan, F. H., Bazargan, M., Herman, J. L., & Chen, Y. T. (2013). Latina transgender women’s interactions with law enforcement in Los Angeles County. *Policing: A Journal of Policy and Practice*, *7*(4), 379-391.

Xavier, J., Bradford, J., Hendricks, M., Safford, L., McKee, R., Martin, E., & Honnold, J. A. (2013). Transgender health care access in Virginia: A qualitative study. *International Journal of Transgenderism*, *14*(1), 3–17.

Yadegarfard, M. (2013). Influences on loneliness, depression, sexual-risk behaviour and suicidal ideation among Thai transgender youth. *Culture, Health & Sexuality*, *15*(6), 726–737.

Yarhouse, M. A., & Carrs, T. L. (2012). MTF transgender Christians’ experiences: A qualitative study. *Journal of LGBT Issues in Counseling*, *6*(1), 18–33.

Yavorsky, J. E., & Sayer, L. (2013). “Doing fear”: The influence of hetero-femininity on (trans)women’s fears of victimization. *Sociological Quarterly*, *54*(4), 511–533.

Yerke, A. F., & Mitchell, V. (2011). Am I man enough yet? A comparison of the body transition, self-labeling, and sexual orientation of two cohorts of female-to-male transsexuals. *International Journal of Transgenderism*, *13*(2), 64–76.

Zanghellini, A. (2010). Queer kinship practices in non-western contexts: French Polynesia’s gender-variant parents and the law of La Republique. *Journal of Law and Society*, *37*(4), 651–677.

Zhang, Y., Lu, L., Zhang, W., Jiang, H., & Zhu, X. (2010). A simple and effective method for phalloplasty in female-to-male transsexuals. *Plastic and Reconstructive Surgery*, *126*(5), 264e–265e.

Zhao, J. J., Marchaim, D., Palla, M. B., Bogan, C. W., Hayakawa, K., Tansek, R., … Kaye, K. S. (2014). Surgical site infections in genital reconstruction surgery for gender reassignment, Detroit: 1984-2008. *Surgical Infections*, *15*(2), 99–104.

Zito, E. (2013). Disciplinary crossings and methodological contaminations in gender research: A psycho-anthropological survey on Neapolitan femminielli. *International Journal of Multiple Research Approaches*, *7*(2), 204–217.

Zitz, C., Burns, J., & Tacconelli, E. (2014). Trans men and friendships: A Foucauldian discourse analysis. *Feminism & Psychology*, *24*(2), 216–237.

Zubiaurre‐Elorza, L., Junque, C., Gómez‐Gil, E., & Guillamon, A. (2014). Effects of cross-sex hormone treatment on cortical thickness in transsexual individuals. *Journal of Sexual Medicine*, *11*(5), 1248–1261.

Zubiaurre-Elorza, L., Junque, C., Gómez-Gil, E., Segovia, S., Carrillo, B., Rametti, G., & Guillamon, A. (2013). Cortical thickness in untreated transsexuals. *Cerebral Cortex*, *23*(12), 2855–2862.

Zucker, K. J., Bradley, S. J., Owen-Anderson, A., Kibblewhite, S. J., Wood, H., Singh, D., & Choi, K. (2012a). Demographics, behavior problems, and psychosexual characteristics of adolescents with gender identity disorder or transvestic fetishism. *Journal of Sex & Marital Therapy*, *38*(2), 151–189.

Zucker, K. J., Bradley, S. J., Owen-Anderson, A., Singh, D., Blanchard, R., & Bain, J. (2011). Puberty-blocking hormonal therapy for adolescents with gender identity disorder: A descriptive clinical study. *Journal of Gay & Lesbian Mental Health*, *15*(1), 58–82.

Zucker, K. J., Wood, H., Singh, D., & Bradley, S. J. (2012b). A developmental, biopsychosocial model for the treatment of children with gender identity disorder. *Journal of Homosexuality*, *59*(3), 369–397.
