# Supplementary material for: Documenting Research with Transgender, Nonbinary, and Other Gender Diverse (Trans) Individuals and Communities: Introducing the Global Trans Research Evidence Map
Source: Transgend Health. 2019 Mar 1;4(1):68–80. doi: 10.1089/trgh.2018.0020 (PMC6400230; doi:10.1089/trgh.2018.0020)
Supplement: Supplemental data [file Supp_Data3.docx]

**Supplementary Data S3: Summary Table of Trans-Focused Studies**

| **First Author and Year of Publication** | **Study Topic** | **Study Design** | **Data Collection Method/Data Source** |
| --- | --- | --- | --- |
| Abdullah (2012) | • Discrimination and marginalization • Sex work | • Qualitative - interviews or FGs | • Interviews • Focus Groups |
| Abelson (2014) | • Violence and trauma • Gender identity and expression | • Qualitative - interviews or FGs | • Interviews |
| Adams (2013) | • Physical health • Therapeutics and surgeries | • Case Report/Case Study/Case Series | • Clinical Records |
| Adenuga (2012) | • Therapeutics and surgeries | • Case Report/Case Study/Case Series | • Clinical Records |
| Agrawal (2013) | • Physical health • Sexual health, HIV, and STIs • Therapeutics and surgeries | • Case Report/Case Study/Case Series | • Clinical Records |
| Ahlin (2014) | • Therapeutics and surgeries | • Cohort studies | • Clinical Records |
| Ahmadzad-Asl (2011) | • Gender identity and expression | • Cross-sectional | • Clinical Records |
| Ahmed (2014) | • Discrimination and marginalization | • Cross-sectional | • Survey - in person |
| Ainsworth (2010) | • Resilience/well-being/QOL • Therapeutics and surgeries | • Cross-sectional | • Survey - online • Survey - in person |
| Akhtar (2012) | • Other • Sexual health, HIV, and STIs | • Cross-sectional | • Clinical Samples/Materials • Survey - in person |
| Alameddine (2011) | • Physical health • Therapeutics and surgeries | • Case Report/Case Study/Case Series | • Clinical Records |
| Algars (2012) | • Gender identity and expression • Mental health • Physical health | • Qualitative - interviews or FGs | • Interviews |
| Alhabshi (2011) | • Biology and physiology • Physical health | • Case Report/Case Study/Case Series | • Clinical Records |
| Altaf (2012) | • Sex work • Sexual health, HIV, and STIs | • Cross-sectional | • Clinical Samples/Materials • Survey - in person |
| Altman (2012) | • Therapeutics and surgeries | • Case Report/Case Study/Case Series | • Clinical Records |
| Altomare (2013) | • Therapeutics and surgeries | • Case Report/Case Study/Case Series | • Clinical Records |
| Amend (2013) | • Therapeutics and surgeries | • Case Report/Case Study/Case Series | • Clinical Records |
| Aminsharifi (2012) | • Therapeutics and surgeries | • Case Report/Case Study/Case Series | • Clinical Records |
| Amirian (2011) | • Therapeutics and surgeries | • Case Report/Case Study/Case Series | • Clinical Records |
| Anderson (2014) | • Therapeutics and surgeries | • Cohort studies | • Clinical Records |
| Andrasik (2014) | • Research methods • Sexual health, HIV, and STIs | • Qualitative - interviews or FGs | • Focus Groups |
| Andreazza (2014) | • Biology and physiology • Early life experiences | • Case Report/Case Study/Case Series | • Clinical interview • Interviews |
| Ansara (2012) | • Discrimination and marginalization • Mental health | • SR Desc and Qual | • Journal Articles - Review |
| Antoszewski (2012) | • Therapeutics and surgeries | • Case Report/Case Study/Case Series | • Clinical interview |
| Aramburu (2013) | • Gender identity and expression • Other • Social support, relationships, and families | • Qualitative - interviews or FGs | • Interviews |
| Arsenault (2012) | • Arts and creativity • Gender identity and expression • Religion and spirituality • Resistance and politicization | • Autoethnography | • Not Discussed |
| Asscheman (2011) | • Physical health • Therapeutics and surgeries | • Cohort studies | • Clinical Records • Other |
| Auer (2013a) | • Biology and physiology | • Cohort studies | • Clinical Records |
| Auer (2013b) | • Gender identity and expression • Mental health • Research methods | • Case-control | • Clinical interview |
| Auer (2014) | • Gender identity and expression • Sexuality | • Cohort studies | • Survey - in person |
| Ayanian (2013) | • Physical health • Therapeutics and surgeries | • Case Report/Case Study/Case Series | • Clinical Records |
| Baba (2011) | • Gender identity and expression • Physical health • Therapeutics and surgeries | • Cross-sectional | • Clinical interview |
| Bailey (2014) | • Health and mental health services • Mental health | • Cross-sectional | • Survey - online |
| Balgos (2012) | • Violence and trauma • Ethnicity, culture, race, and racialization • Resistance and politicization | • Ethnography or phenomenology | • Interviews • Observation |
| Bandini (2011) | • Violence and trauma • Early life experiences • Mental health | • Cross-sectional | • Clinical interview |
| Baradkar (2011) | • Physical health | • Case Report/Case Study/Case Series | • Clinical Records |
| Baral (2013) | • Sexual health, HIV, and STIs | • SR Desc and Qual | • Journal Articles - Review |
| Barišić (2014) | • Mental health | • Cross-sectional | • Clinical interview • Other |
| Barnes (2013) | • Physical health • Therapeutics and surgeries | • Case Report/Case Study/Case Series | • Clinical Records |
| Barnett (2013) | • Arts and creativity • Resistance and politicization • Space and place | • Qualitative - interviews or FGs | • Interviews |
| Bauer (2012) | • Indigeneity • Sexual health, HIV, and STIs | • Cross-sectional • CBR/PAR | • Survey - online • Survey - in person |
| Bauer (2013) | • Sexuality • Sexual health, HIV, and STIs | • Cross-sectional • CBR/PAR | • Survey - online • Survey - in person |
| Bauer (2014) | • Discrimination and marginalization • Health and mental health services | • Cross-sectional • CBR/PAR | • Survey - online • Survey - in person |
| Bazargan (2012) | • Discrimination and marginalization • Violence and trauma • Mental health | • Cross-sectional | • Survey - in person |
| Beagan (2012) | • Employment • Gender identity and expression | • Qualitative - interviews or FGs | • Interviews |
| Benotsch (2013) | • Mental health • Substance use (alcohol and drug use) | • Cross-sectional | • Survey - in person |
| Benson (2013) | • Gender identity and expression • Health and mental health services | • Ethnography or phenomenology | • Interviews |
| Bento (2012) | • Gender identity and expression • Sexuality | • Ethnography or phenomenology | • Interviews |
| Bentz (2010) | • Biology and physiology • Therapeutics and surgeries | • Cohort studies | • Clinical Samples/Materials |
| Berkowitz (2010) | • Arts and creativity • Discrimination and marginalization • Employment • Income • Resilience/well-being/QOL | • Ethnography or phenomenology | • Interviews • Observation |
| Berry (2012) | • Therapeutics and surgeries | • Cohort studies | • Clinical Records |
| Bethea (2013) | • Gender identity and expression • Social support, relationships, and families | • Qualitative - interviews or FGs | • Interviews |
| Bhatta (2014) | • Sexual health, HIV, and STIs | • Cross-sectional | • Survey - in person |
| Bianca (2012) | • Biology and physiology | • Basic Science | • Clinical Samples/Materials |
| Bith-Melander (2010) | • Ethnicity, culture, race, and racialization • Health and mental health services | • Ethnography or phenomenology | • Interviews • Focus Groups |
| Blackburn (2014) | • Arts and creativity • Education • Resistance and politicization • Sexuality | • Qualitative - interviews or FGs | • Interviews |
| Blanchard (2010) | • Gender identity and expression • Mental health • Sexual health, HIV, and STIs | • Cross-sectional | • Clinical interview |
| Blosnich (2013) | • Gender identity and expression • Mental health | • Cross-sectional | • Clinical Records |
| Blumer (2012) | • Gender identity and expression • Mental health | • SR Desc and Qual | • Journal Articles - Review |
| Bockting (2013) | • Discrimination and marginalization • Mental health • Resilience/well-being/QOL • Social support, relationships, and families | • Cross-sectional | • Survey - online |
| Bodoin (2014) | • Gender identity and expression • Health and mental health services | • Cross-sectional | • Survey - online |
| Bogliolo (2014) | • Therapeutics and surgeries | • Case Report/Case Study/Case Series | • Clinical Records |
| Bolger (2014) | • Gender identity and expression • Mental health | • Cross-sectional | • Survey - online • Online sources |
| Boqun (2013) | • Biology and physiology | • Basic Science | • Clinical Samples/Materials |
| Borg (2011) | • Violence and trauma • Mental health • Therapeutic process | • Case Report/Case Study/Case Series | • Clinical Records |
| Boske (2011) | • Discrimination and marginalization • Violence and trauma • Education | • Case Report/Case Study/Case Series | • Clinical Records |
| Boza (2014) | • Discrimination and marginalization • Violence and trauma • Mental health • Social support, relationships, and families | • Cross-sectional | • Survey - online |
| Bradford (2013) | • Discrimination and marginalization • Health and mental health services • Other | • Cross-sectional • CBR/PAR | • Survey - online • Survey - in person |
| Brennan (2012) | • Sexual health, HIV, and STIs | • Cross-sectional • CBR/PAR | • Interviews • Survey - in person |
| Brewster (2012) | • Employment • Gender identity and expression • Research methods | • Cross-sectional | • Survey - online |
| Brewster (2014) | • Employment • Gender identity and expression | • Qualitative - interviews or FGs • Ethnography or phenomenology | • Survey - online |
| Briones (2011) | • Employment • Resilience/well-being/QOL | • Qualitative - interviews or FGs | • Interviews |
| Brown (2010) | • Discrimination and marginalization • Gender identity and expression • Health and mental health services • Law and criminalization • Physical health • Therapeutics and surgeries | • Case Report/Case Study/Case Series | • Other |
| Brown (2012) | • Employment | • Qualitative - interviews or FGs | • Interviews |
| Brown (2013) | • Gender identity and expression | • Qualitative - interviews or FGs | • Interviews |
| Brown (2014) | • Discrimination and marginalization • Health and mental health services • Law and criminalization | • Other | • Other |
| Browne (2010) | • Discrimination and marginalization • Health and mental health services • Resistance and politicization • Space and place | • Cross-sectional • Qualitative - interviews or FGs • CBR/PAR | • Focus Groups • Survey - online • Survey - in person |
| Brunocilla (2012) | • Therapeutics and surgeries | • Cross-sectional | • Clinical Samples/Materials |
| Bucci (2014) | • Therapeutics and surgeries | • Case Report/Case Study/Case Series | • Clinical Records |
| Budge (2010) | • Employment • Gender identity and expression | • Qualitative - interviews or FGs | • Interviews |
| Budge (2013a) | • Mental health • Resilience/well-being/QOL | • Cross-sectional | • Survey - online |
| Budge (2013b) | • Discrimination and marginalization • Gender identity and expression • Resilience/well-being/QOL • Social support, relationships, and families | • Qualitative - interviews or FGs | • Interviews |
| Budge (2014) | • Mental health • Resilience/well-being/QOL • Social support, relationships, and families | • Cross-sectional | • Survey - online |
| Bui (2013) | • Biology and physiology • Therapeutics and surgeries | • Nonrandomized CT | • Clinical Samples/Materials |
| Burdge (2014) | • Gender identity and expression • Resilience/well-being/QOL | • Ethnography or phenomenology | • Interviews |
| Burke (2014a) | • Biology and physiology • Early life experiences | • Cross-sectional | • Clinical Samples/Materials • Clinical interview |
| Burke (2014b) | • Biology and physiology • Early life experiences • Gender identity and expression | • Case-control | • Clinical Samples/Materials |
| Busari (2013) | • Gender identity and expression • Resilience/well-being/QOL | • Nonrandomized CT | • Survey - in person |
| Caldarera (2011) | • Gender identity and expression • Therapeutics and surgeries | • Cross-sectional | • Clinical Records |
| Camp (2011) | • Therapeutics and surgeries | • Case Report/Case Study/Case Series | • Clinical Records |
| Can (2011) | • Gender identity and expression • Law and criminalization | • Case Report/Case Study/Case Series | • Archival records |
| Capitan (2014) | • Therapeutics and surgeries | • Case Report/Case Study/Case Series | • Clinical Records • Survey - in person |
| Carella (2013) | • Physical health • Therapeutics and surgeries | • Case Report/Case Study/Case Series | • Clinical Records |
| Carobene (2014) | • Biology and physiology • Sex work • Sexual health, HIV, and STIs | • Basic Science | • Clinical Samples/Materials |
| Carrillo (2010) | • Biology and physiology | • Cross-sectional | • Clinical Samples/Materials |
| Case (2012) | • Biology and physiology • Gender identity and expression | • Cross-sectional | • Survey - online |
| Caudwell (2014) | • Education • Gender identity and expression • Research methods • Sports/Physical activity | • Qualitative - interviews or FGs | • Interviews |
| Cebula (2010) | • Physical health • Therapeutics and surgeries | • Case Report/Case Study/Case Series | • Clinical Records |
| Cerezo (2014) | • Health and mental health services • Intersectionalities • Migrant and refugee experiences • Resilience/well-being/QOL • Social support, relationships, and families | • Qualitative - interviews or FGs | • Interviews |
| Cerwenka (2014a) | • Mental health • Sexuality • Sexual health, HIV, and STIs • Social support, relationships, and families | • Cross-sectional | • Clinical interview |
| Cerwenka (2014b) | • Sexuality • Sexual health, HIV, and STIs | • Cross-sectional | • Clinical interview |
| Chan (2013) | • Physical health • Therapeutics and surgeries | • Case Report/Case Study/Case Series | • Clinical Records |
| Chandra (2010) | • Biology and physiology • Therapeutics and surgeries | • Cohort studies | • Clinical Samples/Materials |
| Chang (2011) | • Mental health • Sexuality • Therapeutic process | • Case Report/Case Study/Case Series | • Clinical Records |
| Chekir (2012) | • Biology and physiology • Therapeutics and surgeries | • Cross-sectional | • Clinical Samples/Materials |
| Chen (2011) | • Sexual health, HIV, and STIs | • Cross-sectional | • Clinical Records |
| Cho (2012) | • Therapeutics and surgeries | • Case Report/Case Study/Case Series | • Clinical Records |
| Church (2014) | • Gender identity and expression • Parenting, reproduction, and assisted reproduction • Social support, relationships, and families | • Cross-sectional | • Clinical interview • Interviews |
| Cohen (2011) | • Physical health • Therapeutics and surgeries | • Case Report/Case Study/Case Series | • Clinical Records |
| Cohen-Kettenis (2011) | • Therapeutics and surgeries | • Case Report/Case Study/Case Series | • Clinical Records |
| Colebunders (2014) | • Physical health • Therapeutics and surgeries | • Case Report/Case Study/Case Series | • Clinical Records |
| Colizzi (2013) | • Biology and physiology • Mental health • Therapeutics and surgeries | • Cohort studies | • Clinical Samples/Materials • Clinical interview |
| Colizzi (2014) | • Mental health • Therapeutics and surgeries | • Cohort studies | • Clinical interview |
| Connell (2010) | • Employment • Gender identity and expression • Resistance and politicization | • Qualitative - interviews or FGs | • Interviews |
| Connell (2010) | • Gender identity and expression • Historical perspectives | • Qualitative - interviews or FGs | • Interviews |
| Conron (2012) | • Physical health • Research methods | • Cross-sectional | • Other |
| Costa (2014) | • Gender identity and expression • Health and mental health services • Therapeutics and surgeries | • Case Report/Case Study/Case Series | • Clinical Records |
| Costantino (2013) | • Biology and physiology • Resilience/well-being/QOL • Sexual health, HIV, and STIs • Therapeutics and surgeries | • Cohort studies | • Clinical Samples/Materials • Clinical interview • Interviews |
| Cosyns (2014) | • Biology and physiology • Therapeutics and surgeries | • Cross-sectional | • Clinical Samples/Materials • Survey - in person |
| Cotton (2014) | • Discrimination and marginalization • Early life experiences • Education • Social support, relationships, and families | • Case Report/Case Study/Case Series | • Clinical Records |
| Cousino (2014) | • Early life experiences • Gender identity and expression • Health and mental health services • Mental health | • Cross-sectional | • Clinical Records |
| Cregten-Escobar (2012) | • Therapeutics and surgeries | • Cohort studies | • Clinical Records |
| Cruz (2014) | • Discrimination and marginalization • Health and mental health services | • Cross-sectional | • Survey - online • Survey - in person |
| Cupisti (2010) | • Biology and physiology • Therapeutics and surgeries | • Cohort studies | • Clinical Samples/Materials • Clinical interview |
| d'Ythurbide (2012) | • Physical health • Therapeutics and surgeries | • Case Report/Case Study/Case Series | • Clinical Records |
| da Silva (2011) | • Biology and physiology | • Basic Science | • Clinical Samples/Materials |
| Daniolos (2013) | • Early life experiences • Gender identity and expression • Therapeutic process | • Case Report/Case Study/Case Series | • Clinical Records |
| Dargie (2014) | • Gender identity and expression • Mental health • Physical health • Sexuality • Social support, relationships, and families | • Cross-sectional | • Survey - online |
| Dasgupta (2012) | • Mental health | • Case Report/Case Study/Case Series | • Clinical interview |
| Davey (2014) | • Mental health • Resilience/well-being/QOL • Social support, relationships, and families | • Cross-sectional | • Survey - online • Surveys - mail |
| Davies (2013) | • Health and mental health services | • Cross-sectional | • Survey - in person • Surveys - mail |
| Davis (2014) | • Mental health • Sexuality • Therapeutics and surgeries | • Cross-sectional | • Survey - online • Survey - in person • Surveys - mail |
| de Lind van Wijngaarden (2013) | • Discrimination and marginalization • Violence and trauma • Sexual health, HIV, and STIs | • Qualitative - interviews or FGs | • Interviews |
| de Ronde (2011) | • Biology and physiology • Therapeutics and surgeries | • RCT | • Clinical Samples/Materials |
| De Santis (2010) | • Health and mental health services • Sexual health, HIV, and STIs | • Case Report/Case Study/Case Series | • Not Discussed |
| de Vries (2010) | • Disability • Early life experiences • Gender identity and expression | • Cross-sectional | • Clinical Records • Clinical interview |
| de Vries (2011a) | • Gender identity and expression • Mental health | • Cross-sectional | • Clinical interview |
| de Vries (2011b) | • Age and Aging • Mental health • Sexuality | • Cross-sectional | • Clinical interview |
| de Vries (2011c) | • Gender identity and expression • Mental health • Therapeutics and surgeries | • Cohort studies | • Clinical interview |
| de Vries (2012) | • Ethnicity, culture, race, and racialization • Gender identity and expression • Income • Intersectionalities • Sexuality | • Ethnography or phenomenology | • Interviews • Observation |
| de Vries (2014) | • Mental health • Resilience/well-being/QOL • Therapeutics and surgeries | • Cohort studies | • Clinical interview |
| Deipolyi (2010) | • Physical health • Therapeutics and surgeries | • Case Report/Case Study/Case Series | • Clinical Records |
| Deliktas (2014) | • Therapeutics and surgeries | • Case Report/Case Study/Case Series | • Clinical Records |
| Dempf (2010) | • Therapeutics and surgeries | • Case Report/Case Study/Case Series | • Clinical Records |
| Dessy (2014) | • Biology and physiology • Therapeutics and surgeries | • Nonrandomized CT | • Clinical Records |
| Devereaux (2010) | • Discrimination and marginalization • Historical perspectives | • Historical | • Archival records |
| Dhand (2010) | • Health and mental health services • Physical health • Therapeutics and surgeries | • Case Report/Case Study/Case Series | • Clinical Records • Interviews • Observation |
| Dhejne (2011) | • Law and criminalization • Mental health • Physical health • Therapeutics and surgeries | • Cohort studies | • Clinical Records |
| Dhejne (2014) | • Gender identity and expression • Health and mental health services • Law and criminalization • Therapeutics and surgeries | • Cross-sectional | • Clinical Records |
| Dhillon (2011) | • Ethics • Mental health • Therapeutics and surgeries | • Case Report/Case Study/Case Series | • Clinical Records |
| Di Ceglie (2014) | • Disability • Gender identity and expression | • Cross-sectional | • Clinical interview |
| Dickerson (2013) | • Violence and trauma • Mental health • Substance use (alcohol and drug use) | • Case Report/Case Study/Case Series | • Clinical Records |
| Dickey (2012) | • Gender identity and expression • Sexuality | • Qualitative - interviews or FGs | • Interviews • Survey - demographic only |
| Dietert (2013) | • Early life experiences • Education • Resistance and politicization • Social support, relationships, and families | • Qualitative - interviews or FGs | • Interviews |
| Dispenza (2012) | • Discrimination and marginalization • Employment | • Qualitative - interviews or FGs | • Interviews |
| Djordjevic (2013) | • Therapeutics and surgeries | • Case Report/Case Study/Case Series | • Clinical Records |
| Doan (2010) | • Discrimination and marginalization • Gender identity and expression • Resistance and politicization • Space and place | • Autoethnography | • Not Discussed |
| Doorduin (2014) | • Gender identity and expression • Sexuality | • Qualitative - interviews or FGs | • Interviews |
| dos Ramos Farias (2011) | • Biology and physiology • Sex work • Sexual health, HIV, and STIs | • Cross-sectional | • Clinical Samples/Materials • Survey - in person |
| Dowshen (2011) | • Religion and spirituality • Resilience/well-being/QOL • Sexual health, HIV, and STIs | • Cross-sectional | • Clinical interview |
| du Preez (2012) | • Gender identity and expression • Historical perspectives | • Historical | • Archival records |
| Dubois (2012) | • Biology and physiology • Discrimination and marginalization • Gender identity and expression • Therapeutics and surgeries | • Cross-sectional | • Clinical Samples/Materials • Clinical interview • Interviews |
| Dugan (2012) | • Discrimination and marginalization • Education • Social support, relationships, and families | • Cross-sectional | • Survey - online |
| Duisin (2014) | • Mental health | • Cross-sectional | • Clinical interview |
| Dziengel (2014) | • Gender identity and expression • Health and mental health services • Intersectionalities • Physical health • Resistance and politicization • Sexuality • Therapeutics and surgeries | • Autoethnography | • Not Discussed |
| Ecklund (2012) | • Discrimination and marginalization • Early life experiences • Ethnicity, culture, race, and racialization • Intersectionalities • Mental health • Social support, relationships, and families • Therapeutic process | • Case Report/Case Study/Case Series | • Clinical Records |
| Edelman (2011) | • Discrimination and marginalization • Violence and trauma • Ethnicity, culture, race, and racialization • Sex work • Law and criminalization • Resistance and politicization • Space and place | • Qualitative - interviews or FGs | • Interviews |
| Edelman (2014) | • Discrimination and marginalization • Gender identity and expression • Other • Sexuality • Sexual health, HIV, and STIs • Space and place | • Ethnography or phenomenology | • Interviews • Online sources |
| Edwards-Leeper (2012) | • Gender identity and expression • Health and mental health services | • Case Report/Case Study/Case Series | • Clinical Records |
| Effrig (2011) | • Discrimination and marginalization • Violence and trauma • Education • Mental health | • Cross-sectional | • Clinical interview |
| Ehrensaft (2010) | • Early life experiences • Gender identity and expression • Social support, relationships, and families • Therapeutic process | • Case Report/Case Study/Case Series | • Clinical Records |
| Ehrensaft (2013) | • Early life experiences • Education • Gender identity and expression • Mental health • Resilience/well-being/QOL • Social support, relationships, and families • Therapeutics and surgeries • Therapeutic process | • Case Report/Case Study/Case Series | • Clinical Records |
| Ehsanzadeh (2014) | • Mental health • Therapeutics and surgeries | • Case Report/Case Study/Case Series | • Clinical Records |
| Eisner (2012) | • Violence and trauma • Ethnicity, culture, race, and racialization • Resistance and politicization • Sexuality | • Autoethnography | • Observation |
| El Muayed (2010) | • Physical health • Therapeutics and surgeries | • Case Report/Case Study/Case Series | • Clinical Records |
| Elamin (2010) | • Physical health • Therapeutics and surgeries | • SR Desc and Qual | • Journal Articles - Review |
| Elaut (2010) | • Biology and physiology • Sexuality • Therapeutics and surgeries | • Cross-sectional | • Clinical Samples/Materials • Survey - in person |
| Ellis (2014) | • Discrimination and marginalization • Gender identity and expression • Space and place | • Cross-sectional | • Survey - online |
| Erich (2010a) | • Discrimination and marginalization • Ethnicity, culture, race, and racialization • Resilience/well-being/QOL • Social support, relationships, and families | • Cross-sectional | • Surveys - mail |
| Erich (2010b) | • Discrimination and marginalization • Ethnicity, culture, race, and racialization • Intersectionalities • Resilience/well-being/QOL | • Cross-sectional | • Surveys - mail |
| Ertemi (2011) | • Biology and physiology | • Basic Science | • Clinical Samples/Materials |
| Esteva de Antonio (2013) | • Health and mental health services • Therapeutics and surgeries | • Case Report/Case Study/Case Series | • Not Discussed |
| Ettner (2012) | • Discrimination and marginalization • Gender identity and expression • Physical health | • Cross-sectional | • Clinical Records |
| Ewan (2014) | • Gender identity and expression • Mental health • Physical health | • Case Report/Case Study/Case Series | • Clinical Records |
| Fabbre (2014) | • Age and Aging • Employment • Gender identity and expression • Social support, relationships, and families | • Qualitative - interviews or FGs | • Interviews • Observation |
| Fabbri (2014) | • Biology and physiology • Parenting, reproduction, and assisted reproduction | • Basic Science | • Clinical Samples/Materials |
| Faccini (2010a) | • Disability • Violence and trauma • Mental health • Therapeutic process | • Case Report/Case Study/Case Series | • Clinical Records |
| Faccini (2010b) | • Disability • Violence and trauma • Law and criminalization • Mental health • Sexual health, HIV, and STIs • Therapeutic process | • Case Report/Case Study/Case Series | • Clinical Records |
| Faccini (2012) | • Disability • Violence and trauma • Mental health • Sexual health, HIV, and STIs • Therapeutic process | • Case Report/Case Study/Case Series | • Clinical Records |
| Faccio (2013) | • Gender identity and expression • Parenting, reproduction, and assisted reproduction • Social support, relationships, and families | • Qualitative - interviews or FGs | • Interviews |
| Fallon (2012) | • Therapeutic process | • Case Report/Case Study/Case Series | • Clinical Records |
| Feldman (2014a) | • Sexual health, HIV, and STIs | • Cross-sectional | • Interviews • Survey - online |
| Feldman (2014b) | • Gender identity and expression • Mental health • Therapeutics and surgeries | • Case Report/Case Study/Case Series | • Clinical Records |
| Fernandes (2014) | • Physical health • Therapeutics and surgeries | • Case Report/Case Study/Case Series | • Clinical Records |
| Fernández (2014a) | • Biology and physiology | • Basic Science | • Clinical Samples/Materials |
| Fernández (2014b) | • Biology and physiology • Gender identity and expression | • Basic Science | • Clinical Samples/Materials |
| Ferron (2010) | • Age and Aging • Health and mental health services • Sexual health, HIV, and STIs | • Case Report/Case Study/Case Series | • Clinical Records |
| Fink (2014) | • Arts and creativity • Intersectionalities • Other • Resistance and politicization • Sexuality • Social support, relationships, and families • Space and place | • Autoethnography | • Online sources |
| Finkenauer (2012) | • Age and Aging • Discrimination and marginalization | • SR Desc and Qual | • Journal Articles - Review |
| Firth (2014) | • Violence and trauma • Early life experiences • Gender identity and expression • Mental health | • SR Desc and Qual • Other | • Clinical Records • Journal Articles - Review |
| Fischer (2011) | • Physical health • Therapeutics and surgeries | • Case Report/Case Study/Case Series | • Clinical Records |
| Fisher (2010) | • Gender identity and expression • Mental health | • Cross-sectional | • Clinical interview |
| Fisher (2013) | • Gender identity and expression | • Cross-sectional | • Clinical interview |
| Fisher (2014) | • Mental health • Therapeutics and surgeries | • Cross-sectional | • Clinical interview |
| Fletcher (2014) | • Housing • Sexual health, HIV, and STIs | • Cross-sectional | • Clinical interview |
| Flor-Henry (2010) | • Biology and physiology | • Cross-sectional | • Clinical Samples/Materials |
| Fontanari (2013) | • Biology and physiology • Violence and trauma | • Cross-sectional | • Clinical Samples/Materials |
| Francis (2014) | • Discrimination and marginalization • Education • Gender identity and expression • Resistance and politicization • Space and place | • Qualitative - interviews or FGs | • Interviews |
| Furuhashi (2011) | • Early life experiences • Gender identity and expression • Mental health | • Case Report/Case Study/Case Series | • Clinical interview |
| Gabrielli (2010) | • Sexual health, HIV, and STIs • Therapeutics and surgeries | • Case Report/Case Study/Case Series | • Clinical Records |
| Galupo (2014a) | • Social support, relationships, and families | • Cross-sectional | • Survey - online |
| Galupo (2014b) | • Discrimination and marginalization • Sexuality • Social support, relationships, and families | • Cross-sectional | • Survey - online |
| Galupo (2014c) | • Gender identity and expression • Other • Social support, relationships, and families | • Cross-sectional | • Survey - online |
| Ganor (2013) | • Biology and physiology • Sexual health, HIV, and STIs | • Basic Science | • Clinical Samples/Materials |
| Garaffa (2010a) | • Therapeutics and surgeries | • Case Report/Case Study/Case Series | • Clinical Records |
| Garaffa (2010b) | • Therapeutics and surgeries | • Case Report/Case Study/Case Series | • Clinical Records |
| Garcia (2011) | • Employment • Sex work • Income • Space and place | • Ethnography or phenomenology | • Observation |
| Garcia (2014) | • Sexual health, HIV, and STIs • Therapeutics and surgeries | • Cohort studies | • Clinical Records • Clinical Samples/Materials • Interviews |
| García-Malpartida (2010) | • Physical health • Therapeutics and surgeries | • Case Report/Case Study/Case Series | • Clinical Records |
| Garofalo (2012) | • Sexual health, HIV, and STIs | • Cohort studies | • Clinical interview |
| Gelfer (2013a) | • Therapeutics and surgeries | • Cohort studies | • Clinical Samples/Materials |
| Gelfer (2013b) | • Therapeutics and surgeries | • Cohort studies | • Clinical Samples/Materials |
| Gervasoni (2011) | • Physical health • Sexual health, HIV, and STIs • Therapeutics and surgeries | • Case Report/Case Study/Case Series | • Clinical Records |
| Giami (2011) | • Sexual health, HIV, and STIs | • SR Desc and Qual | • Journal Articles - Review |
| Giami (2014) | • Gender identity and expression • Therapeutics and surgeries | • Cross-sectional | • Surveys - mail |
| Godoy (2010) | • Physical health | • Case Report/Case Study/Case Series | • Clinical Records |
| Goldblum (2012) | • Discrimination and marginalization • Violence and trauma • Education • Mental health | • Cross-sectional | • Survey - online • Survey - in person |
| Golub (2010) | • Discrimination and marginalization • Religion and spirituality • Resilience/well-being/QOL • Sexual health, HIV, and STIs • Social support, relationships, and families | • Cross-sectional | • Clinical interview |
| Gómez-Gil (2010) | • Biology and physiology | • Case Report/Case Study/Case Series | • Clinical interview |
| Gomez-Gil (2011) | • Biology and physiology | • Cross-sectional | • Clinical Records |
| Gomez-Gil (2012a) | • Mental health • Research methods | • Cross-sectional | • Clinical interview |
| Gómez-Gil (2012b) | • Mental health • Therapeutics and surgeries | • Cross-sectional | • Clinical Records • Clinical interview |
| Gómez-Gil (2013) | • Other | • Cross-sectional | • Clinical Records • Clinical interview |
| Gómez-Gil (2014) | • Resilience/well-being/QOL | • Cross-sectional | • Clinical interview |
| Gonzalez (2012) | • Mental health • Resilience/well-being/QOL | • Cross-sectional | • Survey - online |
| Goodrich (2012) | • Education • Social support, relationships, and families • Space and place | • Qualitative - interviews or FGs | • Interviews |
| Gooren (2013a) | • Physical health • Resilience/well-being/QOL • Social support, relationships, and families • Therapeutics and surgeries | • Cross-sectional | • Clinical interview |
| Gooren (2013b) | • Physical health • Therapeutics and surgeries | • Cohort studies | • Clinical Records |
| Gooren (2014) | • Physical health | • Cohort studies | • Clinical Records |
| Gorin-Lazard (2012) | • Resilience/well-being/QOL • Therapeutics and surgeries | • Cross-sectional | • Clinical interview |
| Gorin-Lazard (2013) | • Mental health • Resilience/well-being/QOL • Therapeutics and surgeries | • Cross-sectional | • Clinical interview |
| Govier (2010) | • Biology and physiology | • Cross-sectional | • Clinical Samples/Materials • Clinical interview |
| Gower (2010) | • Discrimination and marginalization • Employment • Gender identity and expression | • Case Report/Case Study/Case Series | • Not Discussed |
| Goyal (2014) | • Gender identity and expression • Substance use (alcohol and drug use) | • Case Report/Case Study/Case Series | • Clinical Records |
| Graham (2014a) | • Discrimination and marginalization • Violence and trauma • Education • Ethnicity, culture, race, and racialization • Law and criminalization • Intersectionalities • Religion and spirituality • Resilience/well-being/QOL • Space and place | • Qualitative - interviews or FGs | • Interviews |
| Graham (2014b) | • Ethnicity, culture, race, and racialization • Gender identity and expression • Social support, relationships, and families | • Qualitative - interviews or FGs • CBR/PAR | • Interviews |
| Grant (2010) | • Physical health • Therapeutics and surgeries | • Case Report/Case Study/Case Series | • Clinical Records |
| Grossman (2011) | • Discrimination and marginalization • Mental health • Resilience/well-being/QOL • Social support, relationships, and families | • Cross-sectional | • Clinical interview • Interviews |
| Grynberg (2010) | • Biology and physiology • Therapeutics and surgeries | • Basic Science | • Clinical Records |
| Guadamuz (2011) | • Ethnicity, culture, race, and racialization • Sexual health, HIV, and STIs | • Cross-sectional | • Clinical Samples/Materials • Survey - in person |
| Gupta (2012) | • Mental health • Substance use (alcohol and drug use) | • Case Report/Case Study/Case Series | • Clinical Records |
| Guzman-Parra (2014) | • Discrimination and marginalization • Mental health • Substance use (alcohol and drug use) | • Cross-sectional | • Clinical interview |
| Hagen (2014) | • Discrimination and marginalization • Health and mental health services | • Qualitative - interviews or FGs | • Interviews |
| Hahn (2014) | • Biology and physiology | • Cross-sectional | • Clinical Samples/Materials |
| Haines (2014) | • Gender identity and expression • Intersectionalities • Parenting, reproduction, and assisted reproduction | • Cross-sectional | • Survey - online |
| Hakeem (2012) | • Gender identity and expression • Health and mental health services • Mental health • Therapeutic process | • Case Report/Case Study/Case Series | • Not Discussed |
| Hamdan (2012) | • Therapeutics and surgeries | • Case Report/Case Study/Case Series | • Clinical Records |
| Hancock (2012) | • Therapeutics and surgeries | • Case Report/Case Study/Case Series | • Clinical Samples/Materials • Clinical interview |
| Hancock (2013) | • Health and mental health services • Therapeutics and surgeries | • Case Report/Case Study/Case Series | • Clinical Records |
| Hansen-Reid (2011) | • Ethnicity, culture, race, and racialization • Law and criminalization • Therapeutic process | • Case Report/Case Study/Case Series | • Not Discussed |
| Hariri (2012) | • Physical health • Sexual health, HIV, and STIs • Therapeutics and surgeries | • Case Report/Case Study/Case Series | • Clinical Records |
| Hasegawa (2013) | • Therapeutics and surgeries | • Case Report/Case Study/Case Series | • Clinical Records |
| Hassan (2012) | • Biology and physiology • Therapeutics and surgeries | • Case Report/Case Study/Case Series | • Clinical Records |
| Hedjazi (2013) | • Gender identity and expression | • Cross-sectional | • Clinical interview |
| Hemiliamma (2012) | • Sex work • Sexual health, HIV, and STIs | • Case Report/Case Study/Case Series | • Clinical Samples/Materials |
| Hess (2014) | • Therapeutics and surgeries | • Cross-sectional | • Surveys - mail |
| Hewitt (2012) | • Early life experiences • Health and mental health services • Therapeutics and surgeries | • Cohort studies | • Clinical Records |
| Heylens (2014a) | • Mental health | • Cross-sectional | • Clinical interview • Survey - in person |
| Heylens (2014b) | • Mental health • Therapeutics and surgeries | • Cohort studies | • Surveys - mail |
| Hill (2011) | • Sex work • Sexual health, HIV, and STIs | • Case Report/Case Study/Case Series | • Clinical Records |
| Hiramatsu (2012) | • Biology and physiology • Therapeutics and surgeries | • Cross-sectional | • Clinical Samples/Materials |
| Hisasue (2012) | • Biology and physiology | • Cross-sectional | • Clinical Samples/Materials • Clinical interview |
| Hoebeke (2010) | • Therapeutics and surgeries | • Case Report/Case Study/Case Series | • Clinical Records |
| Hoenig (2011) | • Therapeutics and surgeries | • Case Report/Case Study/Case Series | • Clinical Records |
| Hoffman (2014) | • Mental health | • SR Desc and Qual | • Journal Articles - Review |
| Holmberg (2010) | • Biology and physiology | • Cross-sectional | • Clinical Samples/Materials • Survey - in person |
| Hongal (2014) | • Physical health | • Cross-sectional | • Interviews |
| Horvath (2014) | • Mental health • Sexual health, HIV, and STIs • Space and place • Substance use (alcohol and drug use) | • Cross-sectional | • Survey - online |
| Hoshiai (2010) | • Gender identity and expression • Mental health | • Cross-sectional | • Clinical Records • Clinical interview |
| Hotton (2013) | • Discrimination and marginalization • Resilience/well-being/QOL • Sexual health, HIV, and STIs • Substance use (alcohol and drug use) | • Cross-sectional | • Survey - in person |
| Humphries-Waa (2014) | • Ethnicity, culture, race, and racialization • Health and mental health services • Physical health • Therapeutics and surgeries | • SR Desc and Qual | • Journal Articles - Review |
| Hunt (2014) | • Health and mental health services | • Cross-sectional | • Interviews • Survey - online |
| Hwahng (2014) | • Violence and trauma • Ethnicity, culture, race, and racialization • Income • Intersectionalities • Sexual health, HIV, and STIs | • Cohort studies • Ethnography or phenomenology | • Clinical interview • Interviews |
| Iantaffi (2011) | • Gender identity and expression • Sexuality • Sexual health, HIV, and STIs • Social support, relationships, and families | • Cross-sectional | • Interviews • Survey - online |
| Ikeda (2013) | • Biology and physiology • Therapeutics and surgeries | • Case-control | • Clinical Samples/Materials • Clinical interview |
| Inoubli (2011) | • Biology and physiology | • Basic Science | • Clinical Records |
| Ishikawa (2014) | • Biology and physiology • Parenting, reproduction, and assisted reproduction | • Basic Science | • Clinical Samples/Materials |
| Jackowich (2014) | • Age and Aging • Gender identity and expression | • Case Report/Case Study/Case Series | • Clinical Records |
| James (2011) | • Discrimination and marginalization • Ethnicity, culture, race, and racialization • Health and mental health services • Space and place • Therapeutic process | • Case Report/Case Study/Case Series | • Clinical Records |
| Janssen (2013) | • Gender identity and expression • Mental health • Social support, relationships, and families • Therapeutic process | • Case Report/Case Study/Case Series | • Clinical Records |
| Jauk (2013) | • Discrimination and marginalization • Violence and trauma • Resilience/well-being/QOL • Resistance and politicization | • Ethnography or phenomenology | • Interviews • Observation • Other |
| Jefferson (2013) | • Discrimination and marginalization • Ethnicity, culture, race, and racialization • Intersectionalities • Mental health • Resilience/well-being/QOL | • Cross-sectional | • Clinical interview |
| Jenness (2014) | • Gender identity and expression • Law and criminalization | • Qualitative - interviews or FGs | • Clinical Records • Interviews |
| Johansson (2010) | • Gender identity and expression • Therapeutics and surgeries | • Cohort studies | • Clinical interview |
| Johnson (2014) | • Education • Gender identity and expression • Research methods • Sexuality | • Qualitative - interviews or FGs • CBR/PAR | • Focus Groups • Other |
| Jokic-Begic (2014) | • Mental health • Resilience/well-being/QOL • Therapeutics and surgeries | • Cross-sectional | • Survey - online |
| Judge (2014) | • Gender identity and expression • Health and mental health services • Therapeutics and surgeries | • Cross-sectional | • Clinical Records |
| Junger (2014) | • Biology and physiology | • Cross-sectional | • Clinical Samples/Materials |
| Kalra (2013) | • Ethnicity, culture, race, and racialization • Gender identity and expression • Mental health • Resilience/well-being/QOL • Sexual health, HIV, and STIs | • Cross-sectional | • Clinical interview |
| Kannan (2010) | • Discrimination and marginalization • Health and mental health services • Physical health • Therapeutics and surgeries | • Case Report/Case Study/Case Series | • Clinical Records |
| Kannangara (2012) | • Biology and physiology • Physical health | • Case Report/Case Study/Case Series | • Clinical Records |
| Karpel (2013) | • Mental health • Therapeutics and surgeries | • Case Report/Case Study/Case Series | • Clinical Records |
| Kaufmann (2010a) | • Gender identity and expression • Research methods | • Qualitative - interviews or FGs | • Interviews |
| Kaufmann (2010b) | • Discrimination and marginalization • Ethics • Gender identity and expression • Historical perspectives • Research methods • Resistance and politicization | • Qualitative - interviews or FGs | • Interviews |
| Kaufmann, J. (2014) | • Gender identity and expression • Research methods | • Qualitative - interviews or FGs • Other | • Interviews |
| Kaufmann, U. (2014) | • Biology and physiology • Therapeutics and surgeries | • RCT | • Clinical Samples/Materials |
| Kauth (2014) | • Gender identity and expression • Health and mental health services | • Cross-sectional | • Clinical Records |
| Kedia (2013) | • Biology and physiology | • Basic Science | • Clinical Samples/Materials |
| Kern (2014) | • Early life experiences • Health and mental health services • Social support, relationships, and families • Therapeutic process | • Case Report/Case Study/Case Series | • Clinical Records |
| Khan (2010) | • Physical health • Therapeutics and surgeries | • Case Report/Case Study/Case Series | • Clinical Records |
| Khatchadourian (2014) | • Gender identity and expression • Health and mental health services • Parenting, reproduction, and assisted reproduction • Therapeutics and surgeries | • Cohort studies | • Clinical Records |
| Khazal (2014) | • Disability • Ethics • Physical health • Therapeutics and surgeries | • Case Report/Case Study/Case Series | • Clinical Records |
| Khoosal (2011) | • Social support, relationships, and families • Therapeutics and surgeries | • Cross-sectional | • Clinical Records • Surveys - mail |
| Kim (2010) | • Therapeutics and surgeries | • Case Report/Case Study/Case Series | • Clinical Records |
| Kim (2012) | • Therapeutics and surgeries | • Case Report/Case Study/Case Series | • Clinical Records |
| King (2012) | • Gender identity and expression • Mental health • Therapeutic process | • Case Report/Case Study/Case Series | • Clinical Records |
| Király (2013) | • Biology and physiology | • Basic Science | • Clinical Samples/Materials |
| Kise (2011) | • Gender identity and expression • Mental health • Therapeutic process | • Case Report/Case Study/Case Series | • Clinical Records |
| Knight (2013) | • Physical health • Therapeutics and surgeries | • Case Report/Case Study/Case Series | • Clinical Records |
| Kosenko (2010) | • Discrimination and marginalization • Other • Sexual health, HIV, and STIs | • Qualitative - interviews or FGs | • Interviews |
| Kosenko (2011a) | • Sexual health, HIV, and STIs | • Qualitative - interviews or FGs | • Interviews |
| Kosenko (2011b) | • Sexual health, HIV, and STIs | • Qualitative - interviews or FGs | • Interviews |
| Kosenko (2013) | • Discrimination and marginalization • Health and mental health services | • Cross-sectional | • Survey - online • Surveys - mail |
| Kozee (2012) | • Gender identity and expression • Research methods | • Cross-sectional | • Survey - online |
| Kranz (2014a) | • Biology and physiology | • Cross-sectional | • Clinical Samples/Materials |
| Kranz (2014b) | • Biology and physiology | • Cross-sectional | • Clinical Samples/Materials |
| Krell (2013) | • Arts and creativity • Ethnicity, culture, race, and racialization • Intersectionalities • Resistance and politicization • Therapeutics and surgeries | • Ethnography or phenomenology | • Interviews • Observation • Online sources |
| Kreukels (2011) | • Therapeutics and surgeries | • SR Desc and Qual | • Journal Articles - Review |
| Kreukels (2012) | • Gender identity and expression • Health and mental health services | • Cross-sectional | • Clinical interview |
| Krishnan (2012) | • Physical health | • Case Report/Case Study/Case Series | • Clinical Records |
| Krum (2013) | • Education • Housing • Space and place | • Cross-sectional | • Survey - online |
| Ku (2013) | • Biology and physiology • Discrimination and marginalization | • Nonrandomized CT | • Clinical Records • Clinical Samples/Materials • Clinical interview |
| Kuhn (2011) | • Biology and physiology • Physical health • Therapeutics and surgeries | • Cross-sectional | • Clinical Samples/Materials • Clinical interview |
| Kumar (2012) | • Ethnicity, culture, race, and racialization • Gender identity and expression • Mental health • Resilience/well-being/QOL | • Case Report/Case Study/Case Series | • Clinical Records |
| Künzel (2011) | • Biology and physiology • Therapeutics and surgeries | • Cohort studies | • Clinical Samples/Materials |
| Kuper (2012) | • Gender identity and expression • Sexuality | • Cross-sectional | • Survey - online |
| Kuper (2014) | • Ethnicity, culture, race, and racialization • Gender identity and expression • Intersectionalities • Resilience/well-being/QOL • Sexuality | • Qualitative - interviews or FGs | • Interviews |
| Kurahashi (2013) | • Biology and physiology • Therapeutics and surgeries | • Nonrandomized CT | • Clinical Records • Clinical Samples/Materials |
| Kyoya (2014) | • Biology and physiology • Parenting, reproduction, and assisted reproduction | • Basic Science | • Clinical Samples/Materials |
| Laidlaw (2013) | • Ethics • Physical health • Therapeutics and surgeries | • Case Report/Case Study/Case Series | • Clinical Records • Interviews |
| Lauerma (2010) | • Violence and trauma • Law and criminalization | • Case Report/Case Study/Case Series | • Clinical Records |
| Law (2011) | • Employment • Gender identity and expression | • Cross-sectional | • Survey - online • Survey - in person |
| Lawrence (2010) | • Ethnicity, culture, race, and racialization • Gender identity and expression • Sexuality | • SR Desc and Qual | • Journal Articles - Review |
| Lee (2012) | • Physical health • Therapeutics and surgeries | • Case Report/Case Study/Case Series | • Clinical Records |
| Leibowitz (2011) | • Early life experiences • Health and mental health services | • Case Report/Case Study/Case Series | • Clinical Records |
| Leinung (2013) | • Health and mental health services • Therapeutics and surgeries | • Cross-sectional | • Clinical Records |
| Lemaire (2014) | • Disability • Gender identity and expression | • Case Report/Case Study/Case Series | • Clinical Records |
| Lemma (2012) | • Arts and creativity • Gender identity and expression | • Qualitative - interviews or FGs | • Interviews |
| Lemma (2013) | • Gender identity and expression • Therapeutic process | • Case Report/Case Study/Case Series | • Clinical Records |
| Levitt (2014a) | • Discrimination and marginalization • Gender identity and expression | • Qualitative - interviews or FGs | • Interviews |
| Levitt (2014b) | • Early life experiences • Gender identity and expression • Sexuality | • Qualitative - interviews or FGs | • Interviews |
| Levy (2013) | • Gender identity and expression • Religion and spirituality | • Qualitative - interviews or FGs | • Interviews |
| Lewis (2011) | • Discrimination and marginalization • Gender identity and expression • Resistance and politicization • Space and place | • Qualitative - interviews or FGs | • Interviews |
| Leyngold (2014) | • Physical health | • Case Report/Case Study/Case Series | • Clinical Records |
| Liedtke (2012) | • Physical health • Sexual health, HIV, and STIs | • Case Report/Case Study/Case Series | • Clinical Records |
| Light (2014) | • Parenting, reproduction, and assisted reproduction | • Cross-sectional | • Survey - online |
| Lin (2013) | • Physical health • Therapeutics and surgeries | • Case Report/Case Study/Case Series | • Clinical Records |
| Lin (2014) | • Biology and physiology • Gender identity and expression | • Nonrandomized CT | • Clinical Samples/Materials • Clinical interview |
| Lombardo (2013) | • Biology and physiology • Therapeutics and surgeries | • Basic Science | • Clinical Samples/Materials |
| Longfield (2011) | • Ethnicity, culture, race, and racialization • Sexual health, HIV, and STIs | • Cross-sectional | • Survey - in person |
| Lopez (2011) | • Physical health | • Case Report/Case Study/Case Series | • Clinical Records |
| Luders (2012) | • Biology and physiology | • Cross-sectional | • Clinical Samples/Materials |
| Luecke (2011) | • Early life experiences • Education | • Case Report/Case Study/Case Series | • Interviews • Archival records |
| Macdonald (2013) | • Ethics • Gender identity and expression • Intersectionalities • Research methods • Resistance and politicization | • Autoethnography | • Not Discussed |
| Macdonnell (2012) | • Employment • Gender identity and expression • Health and mental health services • Physical health • Resilience/well-being/QOL | • Qualitative - interviews or FGs | • Interviews |
| Maglione (2014) | • Physical health • Therapeutics and surgeries | • Case Report/Case Study/Case Series | • Clinical Records |
| Maguen (2010) | • Mental health | • Cross-sectional | • Survey - in person |
| Mahalingam (2014) | • Law and criminalization • Therapeutics and surgeries | • Other | • Other |
| Males (2010) | • Physical health • Sexual health, HIV, and STIs • Therapeutics and surgeries | • Case Report/Case Study/Case Series | • Clinical Records |
| Mandlis (2011) | • Discrimination and marginalization • Law and criminalization | • Ethnography or phenomenology | • Observation • Archival records |
| Manieri (2014) | • Biology and physiology • Health and mental health services • Resilience/well-being/QOL • Therapeutics and surgeries | • Cohort studies | • Clinical Samples/Materials • Clinical interview |
| Mann (2011) | • Arts and creativity • Ethnicity, culture, race, and racialization • Gender identity and expression | • Other | • Observation |
| Marciano (2014) | • Gender identity and expression • Other • Resilience/well-being/QOL • Social support, relationships, and families | • Ethnography or phenomenology | • Online sources |
| Marcus (2013) | • Gender identity and expression • Therapeutic process | • Case Report/Case Study/Case Series | • Clinical Records |
| Maree (2014) | • Discrimination and marginalization • Employment • Health and mental health services • Therapeutic process | • Qualitative - interviews or FGs | • Interviews |
| Martin (2011) | • Physical health | • Case Report/Case Study/Case Series | • Clinical Records |
| Martins (2013) | • Sexual health, HIV, and STIs | • Cross-sectional | • Clinical interview |
| Mastronikolis (2013) | • Biology and physiology • Therapeutics and surgeries | • Case Report/Case Study/Case Series | • Clinical Records |
| Masumori (2014) | • Physical health • Therapeutics and surgeries | • Case Report/Case Study/Case Series | • Clinical Records |
| Mathew (2013) | • Health and mental health services • Physical health • Therapeutics and surgeries | • Case Report/Case Study/Case Series | • Clinical Records |
| Maycock (2014) | • Other • Physical health • Therapeutics and surgeries | • SR Desc and Qual | • Journal Articles - Review |
| Mazumder (2013) | • Violence and trauma • Law and criminalization | • Case Report/Case Study/Case Series | • Clinical Records |
| McDuffie (2010) | • Gender identity and expression • Health and mental health services | • Cross-sectional | • Clinical Records |
| McMullin (2011) | • Arts and creativity • Ethnicity, culture, race, and racialization • Gender identity and expression • Migrant and refugee experiences | • Autoethnography | • Not Discussed |
| Meier (2011) | • Mental health • Resilience/well-being/QOL • Social support, relationships, and families • Therapeutics and surgeries | • Cross-sectional | • Survey - online |
| Meier (2013a) | • Gender identity and expression • Sexuality | • Cross-sectional | • Survey - online |
| Meier (2013b) | • Mental health • Social support, relationships, and families | • Cross-sectional | • Survey - online |
| Mendonca (2012) | • Physical health • Therapeutics and surgeries | • Case Report/Case Study/Case Series | • Clinical Records |
| Mepham (2014) | • Health and mental health services • Therapeutics and surgeries | • Cross-sectional | • Surveys - mail |
| Merryfeather (2014) | • Discrimination and marginalization • Health and mental health services | • SR Desc and Qual • Other | • Clinical Records • Other • Journal Articles - Review |
| Meybodi (2014a) | • Mental health | • Cross-sectional | • Clinical interview |
| Meybodi (2014b) | • Mental health | • Cross-sectional | • Clinical interview |
| Mihm (2010) | • Physical health • Sexual health, HIV, and STIs | • Case Report/Case Study/Case Series | • Clinical Records |
| Miller (2012) | • Discrimination and marginalization • Ethnicity, culture, race, and racialization • Sex work • Gender identity and expression • Intersectionalities • Sexuality • Sexual health, HIV, and STIs • Space and place | • Qualitative - interviews or FGs | • Interviews • Focus Groups |
| Miner (2012) | • Other • Research methods | • Cross-sectional | • Survey - online |
| Mishra (2012) | • Ethics • Parenting, reproduction, and assisted reproduction • Sexual health, HIV, and STIs | • Case Report/Case Study/Case Series | • Clinical Records |
| Miyajima (2012) | • Biology and physiology • Physical health • Therapeutics and surgeries | • Cohort studies | • Clinical Samples/Materials |
| Miyajima (2014) | • Mental health | • Cross-sectional | • Clinical interview |
| Mizock (2011) | • Mental health | • Case Report/Case Study/Case Series | • Clinical Records |
| Mizock (2014) | • Discrimination and marginalization • Employment • Mental health • Resilience/well-being/QOL | • Qualitative - interviews or FGs | • Interviews • Survey - in person |
| Mokonogho (2010) | • Housing • Mental health | • Case Report/Case Study/Case Series | • Clinical Records |
| Monsour (2014) | • Gender identity and expression • Social support, relationships, and families | • Qualitative - interviews or FGs | • Interviews |
| Monstrey (2011) | • Therapeutics and surgeries | • Case Report/Case Study/Case Series | • Clinical Records |
| Moody (2013) | • Indigeneity • Mental health • Resilience/well-being/QOL • Social support, relationships, and families | • Cross-sectional | • Survey - online • Surveys - mail |
| Moreman (2010) | • Arts and creativity • Ethnicity, culture, race, and racialization • Gender identity and expression • Intersectionalities • Sexuality • Space and place | • Ethnography or phenomenology | • Observation |
| Morgan (2012) | • Gender identity and expression | • Qualitative - interviews or FGs | • Interviews |
| Motmans (2012) | • Physical health • Resilience/well-being/QOL | • Cross-sectional | • Survey - online • Surveys - mail |
| Motmans (2014) | • Gender identity and expression | • Cross-sectional | • Clinical Records • Surveys - mail |
| Muccino (2014) | • Violence and trauma • Law and criminalization | • Case Report/Case Study/Case Series | • Clinical Records |
| Mueller (2010) | • Biology and physiology • Therapeutics and surgeries | • Cohort studies | • Clinical Samples/Materials |
| Mueller (2011) | • Biology and physiology • Therapeutics and surgeries | • Cohort studies | • Clinical Samples/Materials |
| Muhr (2013) | • Employment • Gender identity and expression | • Qualitative - interviews or FGs | • Interviews |
| Mullen (2013) | • Discrimination and marginalization • Gender identity and expression • Resilience/well-being/QOL • Social support, relationships, and families | • Qualitative - interviews or FGs | • Interviews |
| Murad (2010) | • Mental health • Resilience/well-being/QOL • Sexual health, HIV, and STIs • Therapeutics and surgeries | • SR Desc and Qual | • Journal Articles - Review |
| Murray (2013) | • Gender identity and expression • Mental health • Physical health • Therapeutic process | • Case Report/Case Study/Case Series | • Clinical Records |
| Murty (2010) | • Physical health • Therapeutics and surgeries | • Case Report/Case Study/Case Series | • Clinical Records |
| Nadal (2012) | • Discrimination and marginalization • Gender identity and expression | • Qualitative - interviews or FGs | • Focus Groups |
| Nadal (2014) | • Discrimination and marginalization • Resilience/well-being/QOL | • Qualitative - interviews or FGs | • Focus Groups |
| Nagoshi (2012) | • Gender identity and expression • Intersectionalities • Sexuality | • Qualitative - interviews or FGs | • Interviews |
| Nakamura (2013) | • Biology and physiology • Therapeutics and surgeries | • Cohort studies | • Clinical Samples/Materials • Clinical interview |
| Nash (2011) | • Gender identity and expression • Resistance and politicization • Sexuality • Space and place | • Qualitative - interviews or FGs | • Interviews |
| Nawata (2010) | • Biology and physiology • Gender identity and expression | • Cross-sectional | • Clinical Samples/Materials |
| Nemoto (2011) | • Discrimination and marginalization • Violence and trauma • Ethnicity, culture, race, and racialization • Sex work • Mental health • Social support, relationships, and families | • Cross-sectional | • Clinical interview |
| Nemoto (2012) | • Employment • Sex work • Sexual health, HIV, and STIs | • Cross-sectional | • Survey - in person |
| Nemoto (2014) | • Sexual health, HIV, and STIs | • Cross-sectional | • Clinical interview |
| Neto (2012) | • Therapeutics and surgeries | • Case Report/Case Study/Case Series | • Clinical Records |
| Nichols (2010) | • Violence and trauma • Sex work • Law and criminalization • Intersectionalities • Sexuality | • Qualitative - interviews or FGs | • Interviews • Focus Groups |
| Nichols (2013) | • Arts and creativity • Discrimination and marginalization • Education • Research methods | • Qualitative - interviews or FGs | • Interviews |
| Nieder (2011) | • Age and Aging • Gender identity and expression • Health and mental health services • Sexuality | • Cross-sectional | • Clinical interview |
| Nikolic (2012) | • Physical health • Therapeutics and surgeries | • Case Report/Case Study/Case Series | • Clinical Records |
| Nistal (2013) | • Biology and physiology | • Basic Science | • Clinical Records • Clinical Samples/Materials |
| Nordmarken (2014) | • Gender identity and expression • Resistance and politicization • Therapeutics and surgeries | • Autoethnography | • Not Discussed |
| Nuru (2014) | • Gender identity and expression • Intersectionalities • Other • Social support, relationships, and families | • Other | • Online sources |
| Nuttbrock (2010) | • Age and Aging • Violence and trauma • Mental health | • Cross-sectional • CBR/PAR | • Interviews |
| Nuttbrock (2011) | • Gender identity and expression • Sexuality | • Cross-sectional | • Interviews |
| Nuttbrock (2012) | • Gender identity and expression • Mental health • Research methods • Social support, relationships, and families | • Cross-sectional • CBR/PAR | • Interviews |
| Nuttbrock (2013) | • Violence and trauma • Mental health • Sexual health, HIV, and STIs | • Cohort studies • CBR/PAR | • Clinical Samples/Materials • Interviews |
| Nuttbrock (2014a) | • Violence and trauma • Mental health | • Cohort studies | • Interviews |
| Nuttbrock (2014b) | • Discrimination and marginalization • Violence and trauma • Mental health • Substance use (alcohol and drug use) | • Cohort studies | • Interviews |
| Ocha (2012) | • Ethnicity, culture, race, and racialization • Sex work • Gender identity and expression • Sexuality | • Ethnography or phenomenology | • Interviews |
| Offman (2014) | • Gender identity and expression • Parenting, reproduction, and assisted reproduction • Therapeutic process | • Case Report/Case Study/Case Series | • Clinical Records |
| Oh (2012) | • Biology and physiology • Therapeutics and surgeries | • Cross-sectional | • Clinical Samples/Materials • Clinical interview |
| Operario (2011) | • Sexual health, HIV, and STIs • Social support, relationships, and families | • Cross-sectional | • Survey - in person |
| Operario (2014) | • Discrimination and marginalization • Sexual health, HIV, and STIs • Substance use (alcohol and drug use) | • Cross-sectional | • Survey - in person |
| Oster (2010) | • Physical health • Therapeutics and surgeries | • Case Report/Case Study/Case Series | • Clinical Records |
| Ott (2010a) | • Biology and physiology • Physical health • Therapeutics and surgeries | • Cohort studies | • Clinical Records |
| Ott (2010b) | • Therapeutics and surgeries | • Cohort studies | • Clinical Records |
| Ott (2011) | • Biology and physiology • Therapeutics and surgeries | • Cohort studies | • Clinical Records |
| Paap (2010) | • Gender identity and expression • Therapeutics and surgeries | • Cohort studies | • Clinical interview |
| Paap (2011) | • Gender identity and expression • Mental health • Research methods | • Cross-sectional | • Clinical interview |
| Pacchiarotti (2013) | • Biology and physiology • Parenting, reproduction, and assisted reproduction | • Basic Science | • Clinical Samples/Materials |
| Page (2013) | • Gender identity and expression • Sexuality | • Qualitative - interviews or FGs | • Interviews |
| Palmer (2012) | • Biology and physiology • Gender identity and expression • Therapeutics and surgeries | • Cross-sectional | • Clinical Samples/Materials • Survey - in person |
| Park (2014) | • Physical health • Therapeutics and surgeries | • Case Report/Case Study/Case Series | • Clinical Records |
| Parkinson (2014) | • Disability • Gender identity and expression | • Case Report/Case Study/Case Series | • Clinical Records |
| Parola (2010) | • Resilience/well-being/QOL • Sexual health, HIV, and STIs • Therapeutics and surgeries | • Cross-sectional • Qualitative - interviews or FGs | • Clinical interview • Interviews |
| Pasterski (2014) | • Disability • Gender identity and expression | • Cross-sectional | • Clinical interview • Interviews |
| Pattison (2013) | • Physical health • Therapeutics and surgeries | • Case Report/Case Study/Case Series | • Clinical Records |
| Patton (2010) | • Ethnicity, culture, race, and racialization • Gender identity and expression • Mental health • Therapeutic process | • Case Report/Case Study/Case Series | • Clinical Records |
| Pauley (2014) | • Early life experiences • Gender identity and expression • Social support, relationships, and families • Therapeutic process | • Case Report/Case Study/Case Series | • Clinical Records |
| Pawa (2013) | • Sex work • Health and mental health services • Sexual health, HIV, and STIs | • Cohort studies | • Survey - in person |
| Pelusi (2014) | • Biology and physiology • Resilience/well-being/QOL • Therapeutics and surgeries | • RCT | • Clinical Samples/Materials • Clinical interview |
| Perrin (2010) | • Early life experiences • Gender identity and expression • Therapeutic process | • Case Report/Case Study/Case Series | • Clinical Records |
| Perrone (2010) | • Therapeutics and surgeries | • Case Report/Case Study/Case Series | • Clinical Records |
| Perry (2014) | • Violence and trauma • Discrimination and marginalization | • Qualitative - interviews or FGs | • Interviews |
| Perucchi (2014) | • Discrimination and marginalization • Health and mental health services | • Other | • Observation |
| Petricevic (2014a) | • Biology and physiology • Therapeutics and surgeries | • Basic Science | • Clinical Samples/Materials |
| Petricevic (2014b) | • Biology and physiology • Therapeutics and surgeries | • Basic Science | • Clinical Samples/Materials |
| Pieper (2012) | • Discrimination and marginalization • Historical perspectives • Law and criminalization • Sports/Physical activity | • Historical | • Archival records |
| Pimenoff (2011) | • Health and mental health services • Therapeutics and surgeries | • Cohort studies | • Clinical Records • Surveys - mail |
| Planchenault (2010) | • Gender identity and expression • Other • Social support, relationships, and families | • Other | • Online sources |
| Plemons (2013) | • Health and mental health services • Space and place • Therapeutics and surgeries | • Ethnography or phenomenology | • Observation |
| Pollock (2012) | • Early life experiences • Gender identity and expression | • Qualitative - interviews or FGs | • Interviews |
| Poompruek (2014) | • Gender identity and expression • Substance use (alcohol and drug use) • Therapeutics and surgeries | • Ethnography or phenomenology | • Interviews • Focus Groups • Observation |
| Porch (2014) | • Ethics • Health and mental health services • Mental health • Therapeutic process | • Case Report/Case Study/Case Series | • Clinical Records |
| Porter (2013) | • Age and Aging • Religion and spirituality | • Cross-sectional | • Survey - online |
| Prabawanti (2011) | • Sex work • Sexual health, HIV, and STIs | • Cross-sectional | • Clinical Samples/Materials • Survey - in person |
| Prabawanti (2014) | • Sexual health, HIV, and STIs | • Cross-sectional | • Survey - in person |
| Prinsloo (2011) | • Gender identity and expression • Other • Social support, relationships, and families | • Qualitative - interviews or FGs | • Interviews • Online sources |
| Prunas (2014) | • Mental health • Resilience/well-being/QOL | • Cross-sectional | • Clinical interview |
| Puri (2014) | • Mental health • Substance use (alcohol and drug use) | • Case Report/Case Study/Case Series | • Clinical Records |
| Rachlin (2010) | • Health and mental health services • Therapeutics and surgeries | • Cross-sectional | • Survey - online |
| Raigosa (2013) | • Therapeutics and surgeries | • Case Report/Case Study/Case Series | • Clinical Records |
| Rametti (2011a) | • Biology and physiology | • Cross-sectional | • Clinical Samples/Materials |
| Rametti (2011b) | • Biology and physiology | • Cross-sectional | • Clinical Samples/Materials |
| Rametti (2012) | • Biology and physiology • Therapeutics and surgeries | • Cohort studies | • Clinical Samples/Materials |
| Rankin (2012) | • Education • Gender identity and expression | • Cross-sectional | • Interviews • Survey - online |
| Rapues (2013) | • Research methods • Sexual health, HIV, and STIs | • Cross-sectional | • Clinical Samples/Materials • Survey - in person |
| Reback (2012) | • Sex work • Health and mental health services • Sexual health, HIV, and STIs | • Cohort studies | • Clinical interview |
| Reback (2014) | • Sexual health, HIV, and STIs • Substance use (alcohol and drug use) | • Cross-sectional | • Survey - in person |
| Reed (2011) | • Health and mental health services • Therapeutics and surgeries | • Case Report/Case Study/Case Series | • Clinical Records |
| Rehan (2011) | • Physical health | • Cross-sectional | • Clinical Samples/Materials |
| Reicherzer (2012a) | • Arts and creativity • Ethnicity, culture, race, and racialization • Gender identity and expression • Resistance and politicization | • Qualitative - interviews or FGs | • Interviews |
| Reicherzer (2012b) | • Arts and creativity • Employment • Ethnicity, culture, race, and racialization • Resilience/well-being/QOL | • Qualitative - interviews or FGs | • Interviews • Observation • Archival records |
| Reinsmith-Jones (2013) | • Gender identity and expression • Religion and spirituality | • Ethnography or phenomenology | • Interviews |
| Reisner (2010) | • Gender identity and expression • Health and mental health services • Sexuality • Sexual health, HIV, and STIs | • Cross-sectional • Qualitative - interviews or FGs | • Interviews • Survey - in person |
| Reisner (2013) | • Mental health • Physical health • Resilience/well-being/QOL  • Sports/physical activity SECONDARY | • Cross-sectional • Qualitative - interviews or FGs | • Focus Groups • Survey - in person |
| Reisner (2014a) | • Violence and trauma • Discrimination and marginalization • Ethnicity, culture, race, and racialization • Health and mental health services • Indigeneity • Law and criminalization • Intersectionalities • Physical health | • Cross-sectional | • Survey - online • Survey - in person |
| Reisner (2014b) | • Discrimination and marginalization • Social support, relationships, and families • Substance use (alcohol and drug use) | • Cross-sectional | • Survey - in person |
| Reisner (2014c) | • Research methods | • Case-control • Cross-sectional | • Survey - in person |
| Reisner (2014d) | • Other • Sexual health, HIV, and STIs | • Cross-sectional | • Clinical Records |
| Remacle (2011) | • Therapeutics and surgeries | • Case Report/Case Study/Case Series | • Clinical Records |
| Repessé (2013) | • Physical health • Sexual health, HIV, and STIs | • Case Report/Case Study/Case Series | • Clinical Records |
| Rezwan (2014) | • Physical health • Therapeutics and surgeries | • Case Report/Case Study/Case Series | • Clinical Records |
| Richards (2013) | • Gender identity and expression • Space and place | • Qualitative - interviews or FGs | • Interviews |
| Riggle (2011) | • Gender identity and expression • Resilience/well-being/QOL • Resistance and politicization | • Cross-sectional | • Survey - online |
| Riggs (2014) | • Discrimination and marginalization • Health and mental health services | • Cross-sectional | • Survey - online |
| Rijn (2013) | • Gender identity and expression • Mental health • Social support, relationships, and families | • Cross-sectional | • Clinical interview |
| Riley (2013) | • Early life experiences • Gender identity and expression • Social support, relationships, and families | • Qualitative - interviews or FGs | • Survey - online |
| Roberts (2014) | • Biology and physiology • Therapeutics and surgeries | • Basic Science | • Clinical Records |
| Roerink (2014) | • Physical health • Therapeutics and surgeries | • Case Report/Case Study/Case Series | • Clinical Records |
| Rolle (2014) | • Biology and physiology • Therapeutics and surgeries | • Case-control | • Clinical Samples/Materials |
| Rooke (2010) | • Arts and creativity • Gender identity and expression • Research methods • Resistance and politicization • Space and place | • Qualitative - interviews or FGs • Other • CBR/PAR | • Interviews • Focus Groups • Observation |
| Rosiek (2014) | • Discrimination and marginalization • Research methods | • Ethnography or phenomenology | • Interviews • Observation |
| Ross (2012) | • Violence and trauma • Sex work • Historical perspectives • Law and criminalization • Resistance and politicization • Space and place | • Historical | • Interviews • Archival records |
| Rotondi (2011a) | • Mental health | • Cross-sectional • CBR/PAR | • Survey - online • Survey - in person |
| Rotondi (2011b) | • Mental health | • Cross-sectional • CBR/PAR | • Survey - online • Survey - in person |
| Rotondi (2013) | • Therapeutics and surgeries | • Cross-sectional • CBR/PAR | • Survey - online • Survey - in person |
| Rowniak (2011) | • Gender identity and expression • Sexuality • Sexual health, HIV, and STIs | • Ethnography or phenomenology | • Interviews |
| Rowniak (2013) | • Gender identity and expression • Sexuality | • Ethnography or phenomenology | • Interviews |
| Rupp (2010) | • Arts and creativity • Gender identity and expression • Resistance and politicization | • Ethnography or phenomenology | • Interviews • Focus Groups • Observation • Archival records |
| Sahastrabuddhe (2012) | • Other • Religion SECONDARY • Sexual health, HIV, and STIs | • Cross-sectional | • Survey - in person |
| Saketopoulou (2011) | • Early life experiences • Ethnicity, culture, race, and racialization • Gender identity and expression • Health and mental health services • Income • Intersectionalities • Mental health • Therapeutic process | • Case Report/Case Study/Case Series | • Clinical Records |
| Saketopoulou (2014) | • Violence and trauma • Early life experiences • Gender identity and expression • Therapeutic process | • Case Report/Case Study/Case Series | • Clinical Records |
| Saltzburg (2010) | • Gender identity and expression • Resilience/well-being/QOL • Resistance and politicization • Sexuality | • Qualitative - interviews or FGs | • Focus Groups |
| Salvador (2012) | • Social support, relationships, and families • Therapeutics and surgeries | • Cohort studies | • Survey - in person |
| Samkhaniyani (2013) | • Mental health • Other | • Cross-sectional | • Clinical interview |
| Santarnecchi (2012) | • Biology and physiology | • Cross-sectional | • Clinical Samples/Materials |
| Santos (2014a) | • Sexual health, HIV, and STIs • Substance use (alcohol and drug use) | • Cross-sectional | • Clinical Samples/Materials • Survey - in person |
| Santos (2014b) | • Health and mental health services • Other • Sexual health, HIV, and STIs | • Cross-sectional | • Clinical Samples/Materials • Survey - in person |
| Saravanan (2014) | • Physical health | • Cross-sectional | • Clinical Samples/Materials • Survey - in person |
| Sarrau (2014) | • Physical health • Therapeutics and surgeries | • Case Report/Case Study/Case Series | • Clinical Records |
| Saunders (2011) | • Gender identity and expression • Health and mental health services • Mental health | • Case Report/Case Study/Case Series | • Clinical Records |
| Schagen (2012) | • Biology and physiology • Gender identity and expression | • Cross-sectional | • Clinical Records • Clinical interview |
| Schenck (2010) | • Physical health • Therapeutics and surgeries | • Case Report/Case Study/Case Series | • Clinical Records |
| Schilt (2014) | • Gender identity and expression • Sexuality • Sexual health, HIV, and STIs | • Qualitative - interviews or FGs | • Interviews • Observation |
| Schöning (2010) | • Biology and physiology • Therapeutics and surgeries | • Cross-sectional | • Clinical Samples/Materials • Survey - in person |
| Schor (2011) | • Arts and creativity • Religion and spirituality • Resistance and politicization | • Qualitative - interviews or FGs | • Interviews |
| Schwartz (2010) | • Arts and creativity • Age and Aging • Historical perspectives • Resistance and politicization | • Ethnography or phenomenology | • Not Discussed |
| Seal (2012) | • Therapeutics and surgeries | • Case-control | • Clinical Records |
| Seelman (2014a) | • Discrimination and marginalization • Violence and trauma • Education • Intersectionalities • Space and place | • Qualitative - interviews or FGs | • Interviews |
| Seelman (2014b) | • Discrimination and marginalization • Education • Gender identity and expression | • Cross-sectional | • Survey - online • Survey - in person |
| Seemanthini (2011) | • Mental health | • Cross-sectional | • Survey - in person |
| Sevelius (2010) | • Health and mental health services • Sexual health, HIV, and STIs | • Cross-sectional | • Survey - in person |
| Sevelius (2013) | • Discrimination and marginalization • Ethnicity, culture, race, and racialization • Health and mental health services • Intersectionalities • Sexual health, HIV, and STIs • Social support, relationships, and families | • Qualitative - interviews or FGs | • Interviews |
| Sevelius (2014a) | • Discrimination and marginalization • Health and mental health services • Intersectionalities • Sexual health, HIV, and STIs | • Qualitative - interviews or FGs | • Interviews • Focus Groups |
| Sevelius (2014b) | • Sexual health, HIV, and STIs | • Cross-sectional | • Survey - in person |
| Sexton (2010) | • Law and criminalization • Intersectionalities | • Cross-sectional | • Clinical Records • Interviews |
| Shao (2011) | • Physical health • Therapeutics and surgeries | • Case Report/Case Study/Case Series | • Clinical Records |
| Shepard (2013) | • Health and mental health services • Historical perspectives • Housing • Resistance and politicization • Social support, relationships, and families | • Qualitative - interviews or FGs | • Interviews • Observation • Archival records |
| Shipherd (2010) | • Discrimination and marginalization • Health and mental health services • Mental health | • Cross-sectional | • Survey - in person |
| Shipherd (2011) | • Discrimination and marginalization • Violence and trauma • Mental health • Physical health | • Cross-sectional | • Survey - in person |
| Shipherd (2012) | • Health and mental health services | • Cross-sectional | • Survey - in person |
| Shrestha (2011) | • Health and mental health services • Sexual health, HIV, and STIs | • Cross-sectional | • Clinical Records |
| Shvartsbeyn (2011) | • Physical health • Therapeutics and surgeries | • Case Report/Case Study/Case Series | • Clinical Records |
| Silva-Santisteban (2012) | • Sexual health, HIV, and STIs | • Cross-sectional | • Clinical Samples/Materials • Survey - in person |
| Simon (2011) | • Early life experiences • Gender identity and expression • Mental health • Social support, relationships, and families | • Cross-sectional | • Clinical interview |
| Simon (2013a) | • Biology and physiology | • Cross-sectional | • Clinical Samples/Materials • Clinical interview |
| Simons (2013b) | • Mental health • Resilience/well-being/QOL • Social support, relationships, and families | • Cross-sectional | • Survey - in person |
| Singh (2010) | • Early life experiences • Gender identity and expression • Research methods | • Cross-sectional | • Clinical interview • Survey - online • Survey - in person • Surveys - mail |
| Singh (2011a) | • Discrimination and marginalization • Resilience/well-being/QOL • Resistance and politicization | • Ethnography or phenomenology | • Interviews |
| Singh (2011b) | • Discrimination and marginalization • Violence and trauma • Early life experiences • Ethnicity, culture, race, and racialization • Intersectionalities • Resilience/well-being/QOL | • Ethnography or phenomenology | • Interviews |
| Singh (2013a) | • Discrimination and marginalization • Ethnicity, culture, race, and racialization • Intersectionalities • Other • Resilience/well-being/QOL | • Ethnography or phenomenology | • Interviews |
| Singh (2013b) | • Discrimination and marginalization • Education • Health and mental health services • Resilience/well-being/QOL • Social support, relationships, and families • Space and place | • Ethnography or phenomenology | • Interviews |
| Singh (2014) | • Discrimination and marginalization • Resilience/well-being/QOL | • Ethnography or phenomenology | • Interviews |
| Sitek (2012) | • Biology and physiology | • Cross-sectional | • Clinical Samples/Materials |
| Siverskog (2014) | • Age and Aging • Discrimination and marginalization • Health and mental health services • Intersectionalities | • Qualitative - interviews or FGs | • Interviews |
| Skagerberg (2013a) | • Mental health | • Cross-sectional | • Survey - in person • Surveys - mail |
| Skagerberg (2013b) | • Early life experiences • Mental health | • Cross-sectional | • Clinical Records |
| Skugarevsky (2011) | • Violence and trauma • Sexual health, HIV, and STIs • Substance use (alcohol and drug use) | • Case Report/Case Study/Case Series | • Clinical Records |
| Socias (2014a) | • Discrimination and marginalization • Health and mental health services • Other | • Cross-sectional | • Survey - in person |
| Socias (2014b) | • Gender identity and expression • Law and criminalization | • Cross-sectional | • Survey - in person |
| Soleman (2014) | • Biology and physiology • Therapeutics and surgeries | • Cross-sectional | • Clinical Samples/Materials • Clinical interview |
| Soley-Beltran (2011) | • Gender identity and expression • Historical perspectives • Resistance and politicization | • Qualitative - interviews or FGs | • Interviews • Focus Groups • Online sources |
| Song (2011) | • Therapeutics and surgeries | • Case Report/Case Study/Case Series | • Clinical Records |
| Spack (2012) | • Early life experiences • Gender identity and expression • Health and mental health services • Mental health • Therapeutics and surgeries | • Cross-sectional | • Clinical Records |
| Spack (2013) | • Gender identity and expression • Health and mental health services • Therapeutics and surgeries • Therapeutic process | • Case Report/Case Study/Case Series | • Clinical Records |
| Speer (2013) | • Discrimination and marginalization • Health and mental health services | • Qualitative - interviews or FGs | • Interviews |
| St Peter (2012) | • Health and mental health services • Physical health • Therapeutics and surgeries | • Case Report/Case Study/Case Series | • Clinical Records |
| Steensma (2011) | • Early life experiences • Gender identity and expression • Sexuality | • Qualitative - interviews or FGs | • Interviews |
| Steensma (2013) | • Early life experiences • Gender identity and expression • Mental health • Sexuality • Social support, relationships, and families | • Cohort studies | • Surveys - mail |
| Steensma (2014) | • Early life experiences • Education • Mental health • Social support, relationships, and families | • Cross-sectional | • Clinical interview • Other |
| Stephens (2011) | • Sexual health, HIV, and STIs | • Cross-sectional | • Clinical Samples/Materials • Clinical interview |
| Stotzer (2011) | • Ethnicity, culture, race, and racialization • Resilience/well-being/QOL • Social support, relationships, and families | • Cross-sectional | • Survey - in person |
| Stotzer (2014) | • Law and criminalization | • SR Desc and Qual | • Journal Articles - Review |
| Strain (2011) | • Gender identity and expression • Mental health • Resilience/well-being/QOL | • Cross-sectional | • Survey - in person |
| Suchet (2011) | • Gender identity and expression • Therapeutic process | • Case Report/Case Study/Case Series | • Clinical Records |
| Sultana (2012) | • Discrimination and marginalization • Ethnicity, culture, race, and racialization • Social support, relationships, and families | • Cross-sectional • Qualitative - interviews or FGs | • Interviews • Survey - in person |
| Summers (2014) | • Mental health • Therapeutics and surgeries | • Case Report/Case Study/Case Series | • Clinical Records |
| Syed (2013) | • Ethnicity, culture, race, and racialization • Gender identity and expression • Mental health • Religion and spirituality • Therapeutic process | • Case Report/Case Study/Case Series | • Clinical Records |
| Tagg (2012) | • Discrimination and marginalization • Ethics • Sports/Physical activity | • Qualitative - interviews or FGs | • Interviews |
| Tavakkoli (2014) | • Therapeutics and surgeries | • Cohort studies | • Clinical Samples/Materials • Clinical interview |
| Tayade (2011) | • Gender identity and expression • Physical health • Therapeutics and surgeries | • Case Report/Case Study/Case Series | • Clinical Records |
| Taylor (2011) | • Sexual health, HIV, and STIs | • Cohort studies | • Survey - in person |
| Taylor (2013) | • Ethics • Health and mental health services • Resistance and politicization | • Ethnography or phenomenology | • Interviews |
| Taziaux (2012) | • Biology and physiology | • Basic Science | • Clinical Samples/Materials |
| Tchang (2014) | • Therapeutics and surgeries | • Case Report/Case Study/Case Series | • Clinical Records |
| Ten Kulve (2011) | • Age and Aging • Biology and physiology • Therapeutics and surgeries | • Nonrandomized CT | • Clinical Samples/Materials |
| Terada (2011) | • Gender identity and expression • Mental health | • Cross-sectional | • Clinical Records • Clinical Samples/Materials • Clinical interview |
| Terada (2012a) | • Gender identity and expression • Mental health | • Cross-sectional | • Clinical interview |
| Terada (2012b) | • Early life experiences • Education • Gender identity and expression | • Cross-sectional | • Clinical interview |
| Testa (2012) | • Violence and trauma • Mental health • Substance use (alcohol and drug use) | • Cross-sectional | • Survey - online • Survey - in person • Surveys - mail |
| Testa (2014a) | • Discrimination and marginalization • Violence and trauma • Research methods • Resilience/well-being/QOL • Social support, relationships, and families | • Cross-sectional | • Survey - online |
| Testa (2014b) | • Gender identity and expression • Mental health • Resilience/well-being/QOL • Social support, relationships, and families | • Cross-sectional | • Survey - online |
| Thione (2014) | • Therapeutics and surgeries | • Case Report/Case Study/Case Series | • Clinical Records |
| Thomas (2013) | • Therapeutics and surgeries | • Cohort studies | • Clinical Records |
| Thornhill (2010) | • Health and mental health services • Sexual health, HIV, and STIs • Social support, relationships, and families | • Case Report/Case Study/Case Series | • Clinical Records |
| Tomada (2013) | • Biology and physiology • Therapeutics and surgeries | • Basic Science | • Clinical Samples/Materials |
| Torwane (2014) | • Physical health | • Cross-sectional | • Clinical Samples/Materials • Survey - in person |
| Tourbach (2011) | • Therapeutics and surgeries | • Case Report/Case Study/Case Series | • Clinical Records |
| Traish (2010) | • Therapeutics and surgeries | • SR Desc and Qual | • Journal Articles - Review |
| Travers (2011) | • Gender identity and expression • Resistance and politicization • Social support, relationships, and families • Sports/Physical activity • Space and place | • Qualitative - interviews or FGs | • Interviews • Observation |
| Trevor (2013) | • Gender identity and expression • Health and mental health services | • SR Desc and Qual | • Journal Articles - Review |
| Turo (2013) | • Physical health • Therapeutics and surgeries | • Case Report/Case Study/Case Series | • Clinical Records |
| Ung Loh (2014) | • Gender identity and expression • Religion and spirituality • Social support, relationships, and families | • Qualitative - interviews or FGs | • Interviews |
| Urban (2011) | • Health and mental health services • Physical health • Therapeutics and surgeries | • Case Report/Case Study/Case Series | • Clinical Records |
| Usmani (2012) | • Mental health • Sexual health, HIV, and STIs | • Case Report/Case Study/Case Series | • Clinical Records |
| Van Caenegem (2012) | • Biology and physiology • Therapeutics and surgeries | • Cross-sectional | • Clinical Samples/Materials • Clinical interview |
| Van Caenegem (2013a) | • Biology and physiology | • Cross-sectional | • Clinical Samples/Materials • Clinical interview |
| Van Caenegem (2013b) | • Biology and physiology • Therapeutics and surgeries | • Cross-sectional | • Clinical Samples/Materials • Clinical interview |
| Van Devanter (2012) | • Ethnicity, culture, race, and racialization • Sexual health, HIV, and STIs | • Qualitative - interviews or FGs | • Interviews • Survey - in person |
| Vanderlaan (2011) | • Biology and physiology • Ethnicity, culture, race, and racialization • Gender identity and expression • Sexuality | • Cross-sectional | • Survey - in person |
| VanderLaan (2013) | • Biology and physiology • Ethnicity, culture, race, and racialization • Gender identity and expression • Sexuality | • Cross-sectional | • Interviews • Observation |
| VanKim (2014) | • Education • Physical health • Sports/Physical activity | • Cross-sectional | • Survey - online |
| Vasey (2010) | • Biology and physiology • Sexuality • Social support, relationships, and families | • Cross-sectional | • Survey - in person |
| Veale (2012) | • Gender identity and expression • Resistance and politicization • Sexuality | • Cross-sectional | • Survey - online • Surveys - mail |
| Veale (2014) | • Gender identity and expression • Sexuality | • Cross-sectional | • Survey - online • Surveys - mail |
| Vegter (2013) | • Gender identity and expression | • Ethnography or phenomenology | • Interviews |
| Velayudhan (2014) | • Disability • Mental health • Sexual health, HIV, and STIs | • Case Report/Case Study/Case Series | • Clinical Records |
| Verma (2011) | • Discrimination and marginalization • Employment • Income | • Ethnography or phenomenology | • Observation |
| Victor (2014) | • Biology and physiology • Therapeutics and surgeries | • Cohort studies | • Clinical Samples/Materials |
| Vigneswaran (2013) | • Therapeutics and surgeries | • Case Report/Case Study/Case Series | • Clinical Records |
| Vinay (2010) | • Discrimination and marginalization • Mental health • Therapeutic process | • Case Report/Case Study/Case Series | • Clinical Records |
| Visnyei (2014) | • Physical health • Therapeutics and surgeries | • Case Report/Case Study/Case Series | • Clinical Records |
| Vitelli (2010) | • Violence and trauma • Mental health | • Cross-sectional | • Clinical interview |
| Vivek (2013) | • Discrimination and marginalization • Ethnicity, culture, race, and racialization • Gender identity and expression | • Cross-sectional | • Survey - in person |
| Vujović (2014) | • Biology and physiology • Early life experiences | • Cross-sectional | • Clinical Samples/Materials |
| Vukadinovic (2014) | • Therapeutics and surgeries | • Case Report/Case Study/Case Series | • Clinical Records • Interviews |
| Wagner (2010) | • Therapeutics and surgeries | • Cohort studies | • Clinical Records |
| Walinsky (2010) | • Gender identity and expression • Health and mental health services • Intersectionalities • Resilience/well-being/QOL • Space and place | • Qualitative - interviews or FGs | • Interviews • Focus Groups |
| Wallace (2010a) | • Research methods • Therapeutics and surgeries | • Qualitative - interviews or FGs | • Interviews |
| Wallace (2010b) | • Health and mental health services • Therapeutics and surgeries | • SR Desc and Qual • Cross-sectional | • Interviews • Survey - in person • Journal Articles - Review |
| Wallace (2014) | • Parenting, reproduction, and assisted reproduction • Therapeutics and surgeries | • Case Report/Case Study/Case Series | • Clinical Records |
| Wallien (2010) | • Violence and trauma • Discrimination and marginalization • Education • Gender identity and expression • Social support, relationships, and families | • Cross-sectional | • Survey - in person |
| Walsh (2014) | • Physical health • Therapeutics and surgeries | • Case Report/Case Study/Case Series | • Clinical Records |
| Weigert (2013) | • Physical health • Therapeutics and surgeries | • Cohort studies | • Clinical Records • Clinical interview |
| Weyers (2010c) | • Biology and physiology • Health and mental health services • Therapeutics and surgeries | • Cross-sectional | • Clinical Records • Clinical Samples/Materials • Clinical interview |
| Weyers (2010a) | • Biology and physiology • Health and mental health services • Therapeutics and surgeries | • Cross-sectional | • Clinical Records |
| Weyers (2010b) | • Biology and physiology • Sexuality • Sexual health, HIV, and STIs • Therapeutics and surgeries | • Cross-sectional | • Clinical Samples/Materials • Clinical interview |
| Wierckx (2011a) | • Biology and physiology • Sexual health, HIV, and STIs • Therapeutics and surgeries | • Cross-sectional | • Clinical Samples/Materials • Surveys - mail |
| Wierckx (2011b) | • Resilience/well-being/QOL • Sexual health, HIV, and STIs • Therapeutics and surgeries | • Cross-sectional | • Clinical Samples/Materials • Surveys - mail |
| Wierckx (2012a) | • Biology and physiology • Physical health • Therapeutics and surgeries | • Cross-sectional | • Clinical Samples/Materials • Survey - in person • Surveys - mail |
| Wierckx (2012b) | • Parenting, reproduction, and assisted reproduction | • Cross-sectional | • Clinical Records |
| Wierckx (2012c) | • Parenting, reproduction, and assisted reproduction | • Cross-sectional | • Clinical Samples/Materials • Surveys - mail |
| Wierckx (2013) | • Physical health • Therapeutics and surgeries | • Case-control | • Clinical Records • Survey - online • Survey - in person • Surveys - mail |
| Wierckx (2014a) | • Physical health • Therapeutics and surgeries | • Case Report/Case Study/Case Series | • Clinical Records |
| Wierckx (2014b) | • Sexuality • Sexual health, HIV, and STIs • Therapeutics and surgeries | • Cross-sectional | • Survey - online • Surveys - mail |
| Wierckx (2014c) | • Physical health • Therapeutics and surgeries | • SR Desc and Qual | • Journal Articles - Review |
| Wierckx (2014d) | • Biology and physiology • Therapeutics and surgeries | • Cohort studies | • Clinical Records • Clinical Samples/Materials • Survey - in person |
| Wierckx (2014e) | • Biology and physiology • Therapeutics and surgeries | • Cohort studies • Cross-sectional | • Clinical Samples/Materials • Survey - in person |
| Wight (2014) | • Discrimination and marginalization • Other • Social support, relationships, and families • Space and place | • Ethnography or phenomenology | • Online sources |
| Williams (2012) | • Violence and trauma • Discrimination and marginalization • Health and mental health services • Mental health • Physical health | • Case Report/Case Study/Case Series | • Clinical Records |
| Williams (2013) | • Gender identity and expression • Sexuality • Sexual health, HIV, and STIs • Space and place | • Qualitative - interviews or FGs | • Interviews |
| Wilson (2010) | • Sexual health, HIV, and STIs • Social support, relationships, and families | • Cross-sectional | • Survey - in person |
| Wilson (2011) | • Discrimination and marginalization • Ethnicity, culture, race, and racialization • Sexual health, HIV, and STIs | • Ethnography or phenomenology | • Interviews |
| Wilson (2012) | • Sexual health, HIV, and STIs • Social support, relationships, and families | • Ethnography or phenomenology | • Interviews |
| Wilson (2013) | • Discrimination and marginalization • Violence and trauma • Ethnicity, culture, race, and racialization • Health and mental health services • Sexual health, HIV, and STIs | • Qualitative - interviews or FGs | • Interviews |
| Wilson, D. (2014) | • Health and mental health services | • Cross-sectional | • Clinical Records |
| Wilson, E. (2014a) | • Gender identity and expression • Other • Therapeutics and surgeries | • Cross-sectional | • Survey - in person |
| Wilson, E. (2014b) | • Sexual health, HIV, and STIs | • Cross-sectional | • Clinical Samples/Materials • Survey - in person |
| Winograd (2014) | • Early life experiences • Gender identity and expression • Therapeutic process | • Case Report/Case Study/Case Series | • Clinical Records |
| Witten (2014) | • Age and Aging • Disability • Intersectionalities • Physical health | • Cross-sectional | • Survey - online |
| Wood (2013) | • Early life experiences • Gender identity and expression • Health and mental health services | • Cross-sectional | • Clinical Records |
| Wood (2014) | • Disability • Gender identity and expression | • SR Desc and Qual | • Journal Articles - Review |
| Woods (2013) | • Violence and trauma • Ethnicity, culture, race, and racialization • Law and criminalization | • Cross-sectional | • Survey - in person |
| Xavier (2013) | • Discrimination and marginalization • Violence and trauma • Health and mental health services • Sexual health, HIV, and STIs | • Qualitative - interviews or FGs | • Interviews • Focus Groups |
| Yadegarfard (2013) | • Discrimination and marginalization • Gender identity and expression • Mental health • Sexual health, HIV, and STIs | • Cross-sectional | • Survey - in person |
| Yarhouse (2012) | • Gender identity and expression • Religion and spirituality | • Cross-sectional • Ethnography or phenomenology | • Survey - online |
| Yavorsky (2013) | • Discrimination and marginalization • Violence and trauma • Gender identity and expression • Space and place | • Qualitative - interviews or FGs | • Interviews |
| Yerke (2011) | • Gender identity and expression • Historical perspectives • Sexuality • Therapeutics and surgeries | • Qualitative - interviews or FGs | • Interviews |
| Zanghellini (2010) | • Discrimination and marginalization • Ethnicity, culture, race, and racialization • Law and criminalization • Parenting, reproduction, and assisted reproduction | • Qualitative - interviews or FGs | • Interviews • Survey - in person |
| Zhang (2010) | • Therapeutics and surgeries | • Case Report/Case Study/Case Series | • Clinical Records |
| Zhao (2014) | • Biology and physiology • Therapeutics and surgeries | • Cohort studies | • Clinical Records |
| Zito (2013) | • Ethnicity, culture, race, and racialization • Gender identity and expression • Historical perspectives • Research methods | • Qualitative - interviews or FGs | • Interviews |
| Zitz (2014) | • Gender identity and expression • Social support, relationships, and families | • Qualitative - interviews or FGs | • Interviews |
| Zubiaurre-Elorza (2013) | • Biology and physiology | • Cross-sectional | • Clinical Samples/Materials |
| Zubiaurre-Elorza (2014) | • Biology and physiology • Therapeutics and surgeries | • Cohort studies | • Clinical Samples/Materials • Clinical interview |
| Zucker (2011) | • Early life experiences • Health and mental health services • Mental health • Therapeutics and surgeries | • Cross-sectional | • Clinical Records • Clinical interview |
| Zucker (2012a) | • Early life experiences • Gender identity and expression • Health and mental health services • Sexuality | • Cross-sectional | • Clinical interview |
| Zucker (2012b) | • Early life experiences • Gender identity and expression • Health and mental health services • Mental health • Therapeutic process | • Case Report/Case Study/Case Series | • Clinical Records |
